# The Metabolomic Profile of Microscopic Colitis Is Affected by Smoking but Not Histopathological Diagnosis, Clinical Course, Symptoms, or Treatment

**DOI:** 10.3390/metabo14060303

**Published:** 2024-05-27

**Authors:** Axel Ström, Hans Stenlund, Bodil Ohlsson

**Affiliations:** 1 Clinical Studies Sweden—Forum South, Skåne University Hospital, 22185 Lund, Sweden; axel.strom@skane.se; 2 Umeå Plant Science Centre (UPSC), Department of Plant Physiology, Umeå University, 90187 Umeå, Sweden; hans.stenlund01@umu.se; 3 Department of Clinical Scineces, Lund University, 22100 Lund, Sweden; 4 Department of Internal Medicine, Skane University Hospital, 20502 Malmö, Sweden

**Keywords:** collagenous colitis, lymphocytic colitis, metabolomics, microscopic colitis, smoking habits

## Abstract

Microscopic colitis (MC) is classified as collagenous colitis (CC) and lymphocytic colitis (LC). Genetic associations between CC and human leucocyte antigens (HLAs) have been found, with smoking being a predisposing external factor. Smoking has a great impact on metabolomics. The aim of this explorative study was to analyze global metabolomics in MC and to examine whether the metabolomic profile differed regarding the type and course of MC, the presence of IBS-like symptoms, treatment, and smoking habits. Of the 240 identified women with MC aged ≤73 years, 131 completed the study questionnaire; the Rome III questionnaire; and the Visual Analog Scale for Irritable Bowel Syndrome (VAS-IBS). Blood samples were analyzed by ultra-high-performance liquid chromatograph mass spectrometry (UHLC-MS/UHPLC-MSMS). The women, 63.1 (58.7–67.2) years old, were categorized based on CC (*n* = 76) and LC (*n* = 55); one episode or refractory MC; IBS-like symptoms or not; use of corticosteroids or not; and smoking habits. The only metabolomic differences found in the univariate model after adjustment for false discovery rate (FDR) were between smokers and non-smokers. Serotonin was markedly increased in smokers (*p* < 0.001). No clear patterns appeared when conducting a principal component analysis (PCA). No differences in the metabolomic profile were found depending on the type or clinical course of the disease, neither in the whole MC group nor in the subgroup analysis of CC.

## 1. Introduction

Microscopic colitis (MC) is an inflammatory disorder of the colonic mucosa that predominantly affects elderly women and causes chronic, non-bloody diarrhea with normal or close-to-normal endoscopic findings [1]. MC can be divided into collagenous colitis (CC) and lymphocytic colitis (LC) [2,3]. The histological criteria for CC are a thickened subepithelial collagen layer (>10 µm) in the extracellular matrix (ECM) of the mucosa, epithelial damage, and the presence of an inflammatory infiltrate in the lamina propria [2]. The criteria for LC are >20 intra-epithelial lymphocytes/100 enterocytes [3].

The etiology and pathophysiology of MC are unknown. However, the most well-documented finding is that former and present smoking show a clear association with MC in several studies, and the disease has a three- to fourfold increased prevalence in women compared with men [4,5]. Besides diarrhea, several patients also experience other symptoms such as abdominal pain and constipation [6]. Although MC is considered a chronic disease, almost half of patients have experienced one episode of the disease [7]. Genome-wide association studies (GWASs) have revealed associations between CC and human leucocyte antigens (HLAs) [8], associations which could not be found in LC [9]. Furthermore, a GWAS meta-analysis showed that HLAs have a predisposing role in the pathophysiology of CC but not in LC [10].

Biomarkers are characterized as objective indicators of normal or pathological biological processes or of pharmacological responses to a therapeutic intervention [11]. Metabolomic analyses may identify biomarkers that can define disease phenotypes, identify risks for developing diseases, and predict responses to therapy. Several human studies have identified metabolic differences between smokers and non-smokers [12,13,14,15,16,17]. In a mouse model, 60% of the metabolic differences exhibited reversible changes, while the remaining 40% were irreversible changes, suggesting sustained pathological/adaptive effects from chronic smoking [11].

No biomarker is available for diagnosing MC [1,5,18], and previous research in this cohort has shown normal levels of C-reactive protein (CRP), leucocytes, and albumin [19]. Therefore, histopathological examination of the bowel mucosa is necessary for diagnosis. To our knowledge, global metabolomics has never been examined in MC, although the disease is strongly associated with smoking [4,5]. Furthermore, metabolic changes have been found in other inflammatory bowel diseases (IBDs), such as ulcerative colitis [20]. Our hypothesis was that the identification of metabolic differences between subgroups could provide valuable insights into the mechanisms behind the disease’s development and the clinical course of MC. The aim of the present explorative study was to examine the metabolomic profile of MC to generate hypotheses and to compare the profile regarding the type and course of MC, the presence of IBS-like symptoms, treatment, and smoking.

## 2. Materials and Methods

### 2.1. Patients and Study Design

Women treated for MC at any outpatient clinic of the Departments of Gastroenterology in Skåne, Sweden, between 2002 and 2010 were identified by searching for the ICD-10 classification of the two forms CC and LC (K52.8) in medical records, as well as in the local register at the Department of Pathology, Skåne University Hospital, Malmö. About one-third of the identified patients were excluded due to being over 73, having many other concomitant diseases, or undergoing drug therapies. Of the patients recognized, 240 patients (63 (22–73) years) had their diagnosis verified by colonic biopsy and were <73 years old.

Between March and June 2011, invitations and information were sent to all. Questionnaires, including information about sociodemographic factors, lifestyle habits, medical data, and gastrointestinal symptoms, were dispatched by post to assess the status at the time of inclusion. They were invited to visit the outpatient clinics of the Departments of Gastroenterology, Skåne University Hospital, Malmö, or at the Central Hospital in Kristianstad, Sweden, to provide blood samples. A reminder letter was sent a month after the invitation letter to those who had not answered.

**Figure 1:**
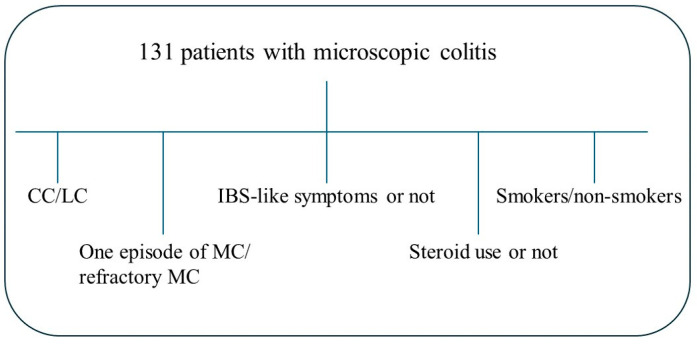
Scheme over the different groups: the cohort was divided into separate analyses: collagenous colitis (CC) (*n* = 76) and lymphocytic colitis (LC) (*n* = 55); one episode (*n* = 58) or refractory microscopic colitis (MC) (*n* = 73); the presence of irritable bowel syndrome (IBS)-like symptoms (*n* = 68) or not (*n* = 63); the use of corticosteroids (*n* = 36) or not (*n* = 95); and smoking (*n* = 44) or non-smoking (*n* = 87).

Of the 240 patients invited, 158 patients (63 (22–73) years) agreed to participate in the study and fulfilled the criteria. Among those, 131 (82.9%) also agreed to provide blood samples and were included in the present study. Questionnaires were completed 1–3 weeks before the blood samples were collected. Medical records were scrutinized, and age, gastrointestinal symptoms, examinations, and treatments were recorded. The patients were characterized as either having CC (*n* = 76) or LC (*n* = 55). The patients were further divided into those with refractory MC with at least two episodes of watery diarrhea; those with a dependence on long-term treatment with corticosteroids to maintain remission; and/or those with two pathological intestinal mucosa biopsies, in line with the criteria suggested for diagnosing IBD [21]. The other group included patients who had had only one episode of severe diarrhea (diarrhea that required examination by colonoscopy) or had had a normal biopsy after the initial pathological intestinal biopsy, in combination with a clinical remission. The patients were also divided into groups depending on (1) if concomitant IBS-like symptoms were present or not; (2) if they concomitantly used corticosteroids or not; and (3) their smoking habits (Figure 1).

Blood was collected from non-fasting patients in heparinized glass tubes and centrifuged at 3000 rcf for 10 min, after which plasma was immediately cooled and stored at −80 °C until later metabolomic analyses. Tests for kidney function and liver enzymes in plasma were analyzed by standard methods at the Department of Clinical Chemistry, Skåne University Hospital, Malmö, Sweden [22].

### 2.2. Questionnaires

#### 2.2.1. Study Questionnaire

A study questionnaire about marital status, education, employment, smoking habits, alcohol consumption, physical activity, medical conditions, and medication was completed by all the participants at home.

#### 2.2.2. Rome III Criteria

The patients completed a shortened version of the Rome III questionnaire, including only IBS symptoms [23]. Patients who fulfilled the criteria for Rome III were classified as also suffering from IBS-like symptoms since their diagnosis was MC, and thus, it cannot be called IBS [6].

#### 2.2.3. Visual Analogue Scale for Irritable Bowel Syndrome (VAS-IBS)

The VAS-IBS is a short, psychometrical test developed to assess gastrointestinal symptoms and psychological well-being during the previous two weeks [24]. The questionnaires include seven items, abdominal pain, diarrhea, constipation, bloating and flatulence, vomiting and nausea, perception of psychological well-being, and the intestinal symptoms’ influence on daily life, graded on a scale from 0 to 100 mm, with 100 mm representing the worst symptoms. The scale is inverted from the original version, and reference values for healthy women are available [24,25].

### 2.3. Metabolite Analyses

Heparin-stabilized plasma was examined at the Swedish Metabolomic Center, Umeå, Sweden. The ultra-high-performance liquid chromatograph mass spectrometry (UHPLC-MS) analysis was performed with an Infinity 1290 Agilent (Agilent Technologies, Santa Clara, CA, USA) coupled with tandem mass spectrometry (UHPLC-MSMS) as previously described in detail [26]. The pre-processing of the UHPLC-MS data has previously been described [27]. In total, 288 metabolites were found in the LC-MS, of which 190 were in a positive mode and 98 were in a negative mode. Forty-three selected oxylipins were pooled and used to make a calibration curve, of which thirty-seven could be detected at trustworthy levels, which led to 325 metabolites altogether. All metabolomics data were relative concentrations.

### 2.4. Statistical Analyses

Statistical analyses regarding sociodemography and comorbidity were performed using software SPSS^©^, version 28 for Windows (IBM, New York, NY, USA). Differences were calculated between CC and LC; one episode or refractory MC; the presence of IBS-like symptoms or not; the use of corticosteroids or not; and never smoking, former smoking, or present smoking. The background values of the variables were not normally distributed, which is why the Mann–Whitney U test or Kruskal–Wallis test was used. Dichotomous variables were calculated by Fisher’s exact test. Values are presented as the median and interquartile range, or the number and percentage. A *p*-value < 0.05 was considered statistically significant.

Regarding metabolites, there was no grouping of the LC-MS metabolites based on age or BMI, so no adjustments were needed for these parameters. The metabolites were first log2-transformed, and differences between groups were compared by Welch’s *t*-tests. The mean differences are presented together with crude *p*-values as well as the adjusted *p*-values for the false discovery rate (FDR) set at 5% according to the Benjamini–Hochberg method [28] to adjust for multiple comparisons and consider our main results. A principal component analysis (PCA) was conducted to visually assess similarities and differences between metabolites and determine whether metabolites can be grouped using R version 4.2.2. [29]. The PCA was used for overview purposes, such as outlier detection, and to deduce the general characteristics of the data, while the statistical tests were used to examine the potential association of each metabolite with the clinical data. Note that all *p*-values were calculated for exploratory purposes only. PCA and Welch’s tests were first performed for the whole group, then repeated only for the patients with CC (*n* = 76) since recent genetic data suggest CC to be a different disease compared with LC [10].

## 3. Results

### 3.1. Basal Characteristics

The median age of the participants was 63.1 (58.7–67.2) years, with a median body mass index (BMI) of 24.8 (22.7–28.7) kg/m^2^. The disease duration was 8 (4–14) years. Seventy-six patients (58.0%) were classified as having CC and 55 (42.0%) as having LC. Of these, 73 patients (55.7%) had refractory MC and 68 (51.9%) also suffered from IBS-like symptoms. The most common comorbidities were hypertension (*n* = 41), thyroid disease (*n* = 30), and rheumatoid arthritis (*n* = 26), and the most used drugs were corticosteroids (*n* = 36), proton pump inhibitors (PPIs) (*n* = 34), thyroid hormones (*n* = 28), selective serotonin reuptake inhibitors (SSRIs) (*n* = 26), and statins (*n* = 26).

Liver and kidney function were normal, assessed by creatinine (70 (62–79) µmol/L), bilirubin (8 (6–11) µmol/L), (ASAT) (0.36 (0.30–0.44) µkat/L; 20.4 (17.0–24.9) U/L), alanine transferase (ALAT) (0.34 (0.27–0.44) µkat/L; 19.3 (15.3-24.9) U/L), gamma-glutamyl transferase (GGT) (0.38 (0.31–0.58) µkat/L; 21.5 (17.6–32.9) U/L), alkaline phosphatase (ALP) (1.1 (1.0–1.3) µkat/L; 62.4 (56.7–73.7) U/L), and amylase (0.49 (0.38–0.61) µkat/L; 27.8 (21.5–34.6) U/L) analyses in plasma.

### 3.2. Differences in Basal Characteristics

#### 3.2.1. Collagenous Colitis/Lymphocytic Colitis

The basal characteristics and sociodemographic factors did not differ between CC and LC (Table 1).

Celiac disease was most common in LC, but there was no difference regarding symptoms. Corticosteroids were most often used in CC, whereas SSRIs were most often used in LC (Table 2).

#### 3.2.2. One Episode of MC/Refractory MC

Smoking was most common in refractory MC, as was the use of corticosteroids (Table 3 and Table 4).

Celiac disease was most common for one episode of MC, with the symptoms constipation and bloating and flatulence being most pronounced compared with refractory MC (Table 4).

#### 3.2.3. Presence of IBS-Like Symptoms/No IBS-Like Symptoms

Besides a slightly lower age in those with IBS-like symptoms, the basal characteristics and sociodemographic factors did not differ between the two groups (Table 5).

Patients with IBS-like symptoms more frequently had a history of rheumatoid arthritis, gastric ulcers, and malignancy, whereas a history of diabetes and thyroid disease was most common in those without IBS-like symptoms. With the exception of constipation, all gastrointestinal symptoms as well as impaired psychological well-being were most prominent in MC with IBS-like symptoms (Table 6).

#### 3.2.4. Corticosteroid Use/No Corticosteroid Use

Corticosteroid users had a higher BMI than non-users, and their use was associated with refractory CC (Table 7). The only symptom affected by the drug was bloating, which was more pronounced in users than in non-users (Table 8).

#### 3.2.5. Smokers/Non-Smokers

Smokers were younger and were more often employed, with longer disease duration, and they more often had a refractory disease in comparison to non-smokers (Table 3 and Table 9). Celiac disease was most common in former smokers. There was no difference in SSRI use between smokers (25.0%) and non-smokers (17.2%) (*p* = 0.355). For the smokers, intestinal symptoms had a more pronounced influence on their daily lives (Table 10).

### 3.3. Metabolomics

There were 44 metabolites that differed between CC and LC, of which 42 decreased and 2 increased in CC. All significant differences disappeared when we adjusted for the FDR. Several of these metabolites were different compounds of lysophospholipids (Supplementary Table S1). Those with one episode of MC had decreased concentrations of 27 metabolites and increased concentrations of 2 metabolites in the univariate analysis, which disappeared after the FDR adjustment (Supplementary Table S2). Only seven metabolites were affected by IBS-like symptoms in the crude, unadjusted calculation (Supplementary Table S3). Among the corticosteroid users, 28 metabolites increased and 10 decreased in the crude calculations, with lower values of cortisone (*p* = 0.004) and cortisol/hydrocortisone (*p* = 0.007) being the most prominent findings (Supplementary Table S4).

The only differences that remained after the FDR adjustment were those found in the smokers compared with the non-smokers. As many as 70 metabolites differed in the crude calculations, of which 55 showed decreased levels in smokers and the other 15 showed increased levels. Of these 70 metabolites, 15 persisted after the FDR adjustment. Serotonin was markedly increased in the smokers compared with the non-smokers, with a relative mean difference of 7.21 (*p* < 0.001). Quinic acid (*p* = 0.035), pyrocatechuic acid (*p* = 0.035), trigolline (*p* = 0.035), and proline (*p* = 0.046) were also found in higher concentrations in the smokers than in the non-smokers. On the contrary, several other compounds, most of them fatty acids, i.e., malic acid (*p* = 0.004), cinnamoylglycine (*p* = 0.005), cholic acid (*p* = 0.007), tetradecanedioic acid (*p* = 0.016), hydroxyoctanoic acid (*p* = 0.022), (S)-3-hydroxyisobutyric acid (*p* = 0.035), docosahexaenoic acid (*p* = 0.035), pimelic acid (*p* = 0.035), sebacic acid (*p* = 0.042), and octadecanedioic acid (*p* = 0.045), were found in lower concentrations in the smokers than in the non-smokers. No differences were observed in any of the oxylipins (Supplementary Table S5).

When calculating for only the CC group, six metabolites differed between those with one episode of the disease and those with refractory CC, fourteen metabolites differed between those with IBS-like symptoms or those without (only one was increased in IBS-like CC), and seventeen differed (nine decreased) between those with or without cortiscosteroid use. All these differences disappeared after the FDR adjustment (Supplementary Tables S6–S8). Twenty-eight metabolites differed between the smokers and the non-smokers, but only the difference in serotonin levels persisted after the FDR adjustment, with a mean difference of 6.97 (Supplementary Table S9).

**Figure 2:**
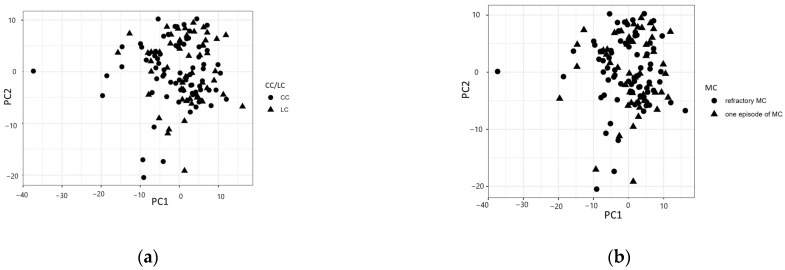

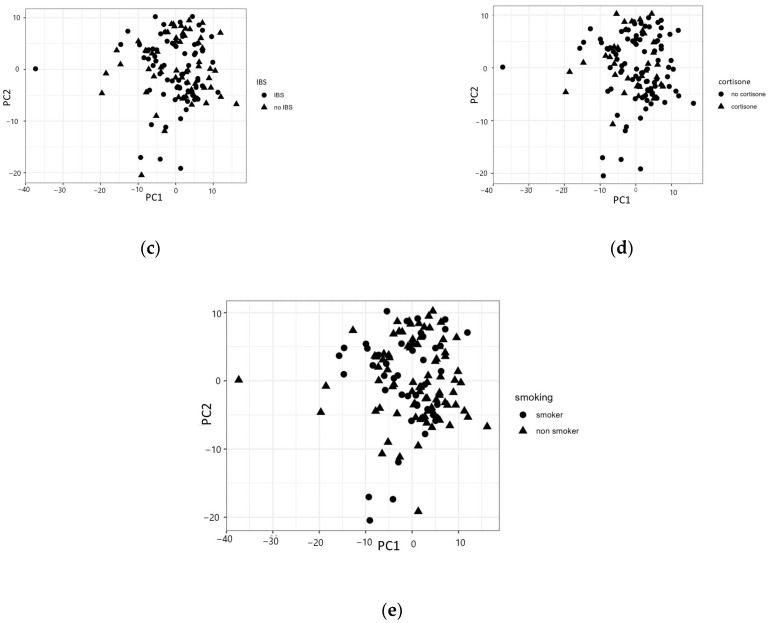
Principal component analysis (PCA) divided by (**a**) collagenous colitis (CC) or lymphocytic colitis (LC); (**b**) one episode of disease or refractory microscopic colitis (MC); (**c**) the presence of irritable bowel syndrome (IBS)-like symptoms or not; (**d**) the use of corticosteroids or not; or (**e**) smoking or non-smoking.

A three-component principal component analysis model explained around 35% of the total variation. Analogous to the univariate results, there were no clear patterns emerging in the components regarding CC/LC, the clinical course of MC, IBS, corticosteroid treatment, or smoking (Figure 2a–e). The same results were found when calculating for only the CC group (Supplementary Figures S1–S4).

## 4. Discussion

The main finding in the present study was that global metabolomics did not differ in MC depending on histopathological diagnosis, clinical course, the presence of IBS-like symptoms, or treatment. Differences in metabolomics after the FDR adjustment were found among smokers and were unrelated to the clinical course. More recent research suggests that CC and LC are different entities, with a close association between HLAs and CC and without any association between HLAs and LC [10]. If this is true, the connection between the two entities may obscure the research field of MC. Therefore, we also performed subgroup analyses in only the CC cohort. However, the same results were also found in the CC cohort, with no effects on the metabolic profile due to the clinical course, symptom profile, or treatment, but smoking habits affected the metabolic composition.

Metabolomics measure both exposure and effect at the same time. If we improve our understanding of metabolic dysregulation in different organs and tissues, we will be able to both understand the mechanisms behind the disease and target therapeutic goals that can be used in clinical practice [30]. The absence of changes in metabolites in the present study is in accordance with the local character of the disease, with histopathological alterations in the bowel mucosa with normal CRP levels [19], without elevated circulating inflammatory markers [5,18]. Further, when dividing the MC patients into groups according to their clinical course and symptoms, only a few differences were found. This contrasts with other IBD conditions, with systemic effects leading to elevated circulating inflammatory biomarkers [21]. Accordingly, the patients with ulcerative colitis had alterations in their metabolic composition depending on the clinical disease state [21]. The presence of IBS-like symptoms among the MC patients with a comorbidity may reflect both the presence of other diseases and the presence of several drug treatments for those patients [31]. The most significant clinical differences observed in the current MC cohort were related to different smoking habits, similarly to the differences in metabolites between the smokers and non-smokers.

The role of cigarette smoking on metabolomics is well established, as was also found in the present study. Cigarette smoking induces several metabolic and inflammatory changes in epithelial cells and tissues, mainly due to oxidative stress [30,32]. The influence of smoking on the gut microbiota is another factor influencing the metabolic profile [17]. These factors may theoretically be of importance for the finding of MC onset about 10 years earlier in smokers than in non-smokers [33,34], which explains the younger age, longer disease duration, and higher degree of working among the smokers.

A decrease in glycerophospholipids, a compound involved in inflammation and immune responses, could be identified after smoking in several studies [17,35], whereas glycerophospholipids were increased after smoking in a mouse model [11]. Sixty-two biomarkers for cigarette smoking could be determined in a small study of nine volunteers [36]. Similar results have been found in larger epidemiological studies with associations with xenobiotic metabolites, amino acids, lipids, vitamins, and cofactors [12]. Smoking cessation led to several changes in urinary levels of prostaglandins, thromboxanes, and leukotrienes when calculating differences over time without adjustment for confounders [14]. These changes, found in healthy male and mixed cohorts, were not the same as those found between the smokers and non-smokers in the present female MC cohort. Nevertheless, the obvious differences observed in the smokers in the present study confirm that the data are of high quality and trustworthy. Poor longitudinal stability has been described in long-term studies, illustrating how multiple endogenous and exogenous factors influence both oxidative stress and inflammation [15]. Further, studies examining the acute effect of smoking have shown that the type of cigarettes smoked, sex, and race also affect metabolic changes [17], which can explain the divergent findings from different studies. In the current study, only women were included, and we do not know the type of cigarette they used or when they smoked their last cigarette in relation to the blood sampling.

Although most cigarette smoke metabolites in plasma decreased after 2 months of smoking cessation in a mouse model, 40% of endogenous plasma metabolites remained affected [11]. This may explain the increased risk of MC in past smokers, although the risk is lower than in present smokers [3,5]. Current smoking appeared to be more strongly associated with CC than with LC in both cohort studies and in a meta-analysis [37,38]. In the present cohort, smoking was only associated with refractory MC. However, no metabolomic differences could be found between those with one episode of MC or refractory MC, although the smoking habits differed, suggesting that the effect of smoking in MC is exerted through local factors and not through systemic factors.

The use of SSRIs was similar among the smokers and the non-smokers. Still, the plasma levels of serotonin were markedly elevated in the smokers, as has also been found previously [39]. A meta-analysis described how smoking elevates serotonin and regional serotonin transporter (SERT) binding in some areas of the limbic system in adolescent rats, whereas human studies in the field are very sparse [40]. Human studies often show conflicting results due to different smoking habits and different study designs, but nicotine has been shown to both stimulate serotonin release from blood platelets and inhibit serotonin uptake by platelets [41]. Mixed evidence suggests that nicotine increases extracellular serotonin levels [41]. The regulation of serotonin levels by smoking may affect cognition, reward systems, and the development of an addiction [40]. However, the differences in serotonin levels were not reflected in different levels of psychological well-being, although gastrointestinal symptoms had more influence on the daily lives of the smokers. The lower levels of cortisone and cortisol/hydrocortisone in the corticosteroid users may be dependent on the downregulation of endogenous steroid synthesis [42].

There are several limitations to the present study. The cross-sectional design of the study makes it impossible to examine whether the differences in metabolites are initial changes or compensatory changes secondary to the other effects induced. Since we used only patients and no healthy controls, we cannot compare the differences related to smoking depending on health and disease. That only women and not men were analyzed is a limitation. However, the disease is much more common in women [5], and only a small cohort of men could be recruited for the study, which made it impossible to perform any statistical calculations for men. Many of the studies that have described metabolic changes due to smoking habits have used ANOVA and other univariate analyses [11], whereas a previous study with a multivariate analysis showed no differences in metabolites between different smoking habits [16]. The same results were found in the present study. When using the FDR, there is a risk of overadjusting the findings, and true differences may disappear.

## 5. Conclusions

In conclusion, in our univariate and multivariate models, we could not identify any metabolomic differences in MC related to histopathological classification, the clinical course of the disease, symptoms, or treatment. Thus, MC likely depends mostly on local factors, with no or minimally induced systemic effects that could modulate the character or course of the disease. As described in several previous studies, smoking has strong effects on the concentrations of metabolomics. The importance of these metabolites for the onset and clinical course of MC remains to be elucidated. Local factors should be of greater importance to study, with measurements of metabolomics in mucosa and epithelial cells from healthy and diseased patients. This may be combined with experimental studies on smoke exposure before sample collection.

## Figures and Tables

**Table 1:** Basal characteristics depending on histopathological classification.

	Collagenous Colitis *N* = 76	Lymphocytic Colitis *N* = 55	*p*-Value
Age (year)	63.7 (59.2–68.4)	62.6 (53.9–66.6)	0.099
BMI (kg/m^2^) Missing value	24.7 (21.8–29.2) 23	24.9 (23.0–27.4) 20	0.941
Refractory MC (*n*, %)	45 (59.2)	28 (50.9)	0.377
IBS (*n*, %)	39 (51.3)	29 (52.7)	1.00
Disease duration (year) Missing value	8 (3–13) 7	8 (4–16) 4	
University education (*n*, %) Missing value	23 (30.3) 3	21 (38.2) 1	0.584
Occupation (*n*, %)			1.00
Employed	34 (44.7)	24 (43.6)	
Retired	38 (50.0)	28 (50.9)	
Others	4 (5.3)	3 (5.5)	
Married/living together (*n*, %) Missing value	47 (61.8) 1	27 (49.1)	0.152
Smoking (*n*, %)			0.394
Never	20 (26.3)	16 (29.1)	
Stopped smoking	27 (35.5)	24 (43.6)	
Smoker	29 (38.2)	15 (27.3)	
Number of days drinking Missing value	5.5 (2.0–10.0) 24	4.0 (2.0–10.0) 13	0.663
Number of standard glasses each day of drinking Missing value	32	17	0.159
Never drinking	3 (3.9)	8 (14.5)	
1	11 (14.5)	4 (7.3)	
2	24 (31.6)	17 (30.9)	
3	5 (6.6)	5 (9.1)	
4	1 (1.3)	3 (5.5)	
≥5		1 (1.8)	

Values presented as a number (percent) or median (interquartile range). Fisher’s exact test or Mann–Whitney U test was used. A *p*-value < 0.05 was considered statistically significant.

**Table 2:** Comorbidity and symptoms for collagenous colitis (CC) or lymphocytic colitis (LC).

	CC *N* = 76	LC *N* = 55	*p*-Value
Comorbidity (*n*, %)			
Asthma and/or bronchitis Missing value	15 (19.7) 11	7 (12.7) 9	0.528
Diabetes Missing value	6 (7.9) 12	5 (9.1) 9	0.906
Cancer Missing value	8 (11.0) 14	3 (5.5) 9	0.584
Celiac disease Missing value	2 (2.6) 16	7 (12.7) 11	0.034
Gastric ulcer Missing value	11 (15.1) 13	10 (18.2) 7	0.923
Hypertension Missing value	27 (35.5) 10	14 (25.5) 4	0.185
Rheumatoid arthritis Missing value	13 (17.1) 12	13 (23.6) 7	0.710
Thyroid disease Missing value	14 (18.7) 10	16 (29.1) 5	0.382
Drug treatment (*n*, %)			
Corticosteroid treatment	28 (36.8)	8 (14.5)	0.005
Proton pump inhibitors	20 (26.3)	14 (25.5)	1.00
Selective serotonin reuptake inhibitors	10 (13.2)	16 (29.1)	0.028
Statins	16 (21.1)	10 (18.2)	0.825
Thyroid hormones	16 (21.1)	12 (21.8)	1.00
Gastrointestinal symptoms (mm)			
Abdominal pain 5 (1–15)	35 (0–52)	39 (12–55)	0.289
Diarrhea 3 (0–10)	38 (14–78)	50 (28–72)	0.306
Constipation 9 (1–22)	6 (2–18)	8 (4–28)	0.236
Bloating and flatulence 14 (1–29)	55 (21–77)	56 (27–71)	0.731
Vomiting and nausea 2 (0–3)	8 (0–23)	10 (2–34)	0.211
Intestinal symptom’s influence on daily life 2 (0–18)	42 (10–72)	41 (26–82)	0.185
Psychological well-being 4 (0–16)	24 (8–51)	23 (10–49)	0.858

Gastrointestinal symptoms were assessed by the Visual Analog Scale for Irritable Bowel Syndrome (VAS-IBS) on a scale of 0–100 mm, where 0 represents no symptoms and 100 represents maximal symptoms [24]. Reference values are for healthy controls [25]. Values were presented as a number (percent) or median (interquartile range). Fisher’s exact test or Mann–Whitney U test was used. A *p*-value < 0.05 was considered statistically significant.

**Table 3:** Basal characteristics depending on one episode of microscopic colitis (MC) or refractory MC.

	One Episode of MC *N* = 58	Refractory MC *N* = 73	*p*-Value
Age (year)	63.8 (59.5–68.0)	62.6 (57.1–67.2)	0.359
BMI (kg/m^2^) Missing value	25.4 (24.0–29.2) 20	24.1 (21.7–27.5) 23	0.063
CC/LC (*n*, %)	31 (53.4)/27 (46.6)	45 (61.6)/28 (38.4)	0.377
IBS (*n*, %)	30 (51.7)	38 (52.1)	1.00
Disease duration (year) Missing value	7 (4–12) 5	10 (4–16) 6	0.187
University education (*n*, %) Missing value	17 (29.3) 2	27 (37.0) 2	0.667
Occupation (*n*, %)			0.413
Employed	22 (37.9)	36 (49.3)	
Retired	32 (55.2)	34 (46.6)	
Others	4 (6.9)	3 (4.1)	
Married/living together (*n*, %) Missing value	36 (62.1) 1	38 (52.1)	0.217
Smoking (*n*, %)			0.003
Never	13 (22.4)	23 (31.5)	
Stopped smoking	32 (55.2)	19 (26.0)	
Smoker	13 (22.4)	31 (42.5)	
Number of days drinking Missing value	4.5 (2.8–10.5) 16	4.5 (1.2–9.5) 21	0.323
Number of standard glasses each day of drinking Missing value	22	27	0.508
Never drinking	4 (6.9)	6 (8.2)	
1	6 (10.3)	1 (1.4)	
2	16 (27.6)	9 (12.3)	
3	7 (12.1)	25 (34.2)	
4	2 (3.4)	3 (4.1)	
≥5	1 (1.7)	2 (2.7)	

Values presented as a number (percent) or median (interquartile range). Fisher’s exact test or Mann–Whitney U test was used. A *p*-value < 0.05 was considered statistically significant.

**Table 4:** Comorbidity and symptoms for one episode or refractory microscopic colitis (MC).

	One Episode of MC *N* = 58	Refractory MC *N* = 73	*p*-Value
Comorbidity (*n*, %)			
Asthma and/or bronchitis Missing value	10 (17.2) 8	12 (16.4) 12	1.00
Diabetes Missing value	4 (6.9) 9	7 (9.6) 12	0.906
Cancer Missing value	3 (5.4) 10	8 (11.1) 13	0.458
Celiac disease Missing value	8 (13.8) 13	1 (1.4) 14	0.010
Gastric ulcer Missing value	9 (15.8) 8	12 (16.9) 12	0.962
Hypertension Missing value	18 (31.0) 6	23 (31.5) 8	1.00
Rheumatoid arthritis Missing value	11 (19.0) 7	15 (20.5) 12	0.549
Thyroid disease Missing value	17 (29.3) 4	13 (18.1) 11	0.208
Drug treatment (*n*, %)			
Corticosteroid treatment	10 (17.2)	26 (35.6)	0.029
Proton pump inhibitors	11 (19.0)	23 (31.5)	0.114
Selective serotonin reuptake inhibitors	8 (13.8)	18 (24.7)	0.131
Statins	12 (20.7)	14 (19.2)	0.830
Thyroid hormones	16 (27.6)	12 (16.4)	0.138
Gastrointestinal symptoms (mm)			
Abdominal pain 5 (1–15)	45 (12–54)	31 (12–52)	0.206
Diarrhea 3 (0–10)	48 (19–71)	46 (18–74)	0.951
Constipation 9 (1–22)	13 (5–32)	6 (2–13)	0.004
Bloating and flatulence 14 (1–29)	60 (31–78)	46 (18–70)	0.035
Vomiting and nausea 2 (0–3)	10 (2–32)	6 (0–24)	0.165
Intestinal symptom’s influence on daily life 2 (0–18)	46 (18–83)	39 (17–74)	0.396
Psychological well-being 4 (0–16)	24 (8–49)	23 (10–51)	0.984

Gastrointestinal symptoms were assessed by the Visual Analog Scale for Irritable Bowel Syndrome (VAS-IBS) on a scale 0–100 mm, where 0 represents no symptoms and 100 represents maximal symptoms [24]. Reference values are from healthy controls [25]. Values are presented as a number (percent) or median (interquartile range). Fisher’s exact test or Mann–Whitney U test was used. A *p*-value < 0.05 was considered statistically significant.

**Table 5:** Basal characteristics depending on IBS-like symptoms or not.

	IBS *N* = 68	No IBS *N* = 63	*p*-Value
Age (year)	61.8 (56.6–66.7)	64.9 (59.6–68.4)	0.046
BMI (kg/m^2^) Missing value	25.1 (23.0–29.3) 17	24.6 (21.6–26.5) 26	0.065
Refractory MC (*n*, %)	38 (55.9)	35 (55.6)	1.00
CC/LC (*n*, %)	39 (57.4)/29 (42.6)	37 (58.7)/26 (41.3)	1.00
Disease duration (year) Missing value	9 (4–20) 3	7 (3–12) 8	0.115
University education (*n*, %) Missing value	25 (36.8) 3	19 (30.2) 1	0.421
Occupation (*n*, %)			0.305
Employed	34 (50.0)	24 (38.1)	
Retired	30 (44.1)	36 (57.1)	
Others	4 (5.9)	3 (4.8)	
Married/living together (*n*, %) Missing value	37 (55.2) 1	37 (58.7)	0.725
Smoking (*n*, %)			0.134
Never	18 (26.5)	18 (28.6)	
Stopped smoking	22 (32.4)	29 (46.0)	
Smoker	28 (41.2)	16 (25.4)	
Number of days drinking Missing value	4.5 (1.8–10.0) 18	4.5 (2.0–10.0) 19	0.749
Number of standard glasses each day of drinking Missing value	22	27	0.465
Never drinking	7 (10.3)	4 (6.4)	
1	10 (14.7)	5 (7.9)	
2	20 (29.4)	21 (33.3)	
3	7 (10.3)	3 (4.8)	
4	2 (2.9)	2 (3.2)	
≥5	0	1 (1.6)	

Values presented as a number (percent) or median (interquartile range). Fisher’s exact test or Mann–Whitney U test was used. A *p*-value < 0.05 was considered statistically significant.

**Table 6:** Comorbidity and symptoms depending on IBS-like symptoms or not.

	IBS *N* = 68	No IBS *N* = 63	*p*-Value
Comorbidity (*n*, %)			
Asthma and/or bronchitis Missing value	12 (17.6) 6	10 (15.9) 14	0.062
Diabetes Missing value	4 (5.9) 6	7 (11.1) 15	0.020
Cancer Missing value	6 (9.1) 7	5 (8.1) 16	0.033
Celiac disease Missing value	5 (7.4) 10	4 (6.3) 17	1.00
Gastric ulcer Missing value	14 (21.2) 6	7 (11.3) 14	0.024
Hypertension Missing value	22 (32.4) 4	19 (30.2) 10	0.190
Rheumatoid arthritis Missing value	20 (29.4) 5	6 (9.5) 14	0.001
Thyroid disease Missing value	15 (22.4) 4	15 (23.8) 11	0.043
Drug treatment (*n*, %)			
Corticosteroid treatment	22 (32.4)	14 (22.2)	0.241
Proton pump inhibitors	20 (29.4)	14 (22.2)	0.426
Selective serotonin reuptake inhibitors	14 (20.6)	12 (19.0)	1.00
Statins	9 (13.2)	17 (27.0)	0.078
Thyroid hormones	13 (19.1)	15 (23.8)	0.531
Gastrointestinal symptoms			
Abdominal pain 5 (1–15)	52 (33–66)	13 (4–36)	<0.001
Diarrhea 3 (0–10)	65 (34–82)	30 (10–51)	<0.001
Constipation 9 (1–22)	8 (3–30)	7 (2–18)	0.272
Bloating and flatulence 14 (1–29)	69 (34–78)	40 (14–59)	<0.001
Vomiting and nausea 2 (0–3)	11 (4–43)	4 (0–19)	0.010
Intestinal symptom’s influence on daily life 2 (0–18)	66 (36–84)	22 (7–49)	<0.001
Psychological well-being 4 (0–16)	30 (13–55)	17 (7–32)	0.004

Gastrointestinal symptoms were assessed by the Visual Analog Scale for Irritable Bowel Syndrome (VAS-IBS) on a scale of 0–100 mm, where 0 represents no symptoms and 100 represents maximal symptoms [24]. Reference values from healthy controls [25]. Values are presented as a number (percent) or median (interquartile range). Fisher’s exact test or Mann–Whitney U test was used. A *p*-value < 0.05 was considered statistically significant.

**Table 7:** Basal characteristics depending on corticosteroid use.

	Corticosteroids *N* = 36	No Corticosteroids *N* = 95	*p*-Value
Age (year)	62.4 (59.0–67.1)	63.8 (57.8–67.7)	0.717
BMI (kg/m^2^) Missing value	27.1 (22.5–29.7) 8	24.3 (22.8–26.4) 35	0.036
Refractory MC (*n*, %)	26 (72.2)	47 (49.5)	0.029
CC/LC (*n*, %)	28 (77.8)/8 (22.2)	48 (50.5)/47 (49.5)	0.005
IBS (*n*, %)	22 (61.1)	46 (48.4)	0.241
Disease duration (year) Missing value	10 (4–16) 5	7 (4–13) 6	0.405
University education (*n*, %) Missing value	13 (36.1) 1	31 (32.6) 3	0.930
Occupation (*n*, %)			1.00
Employed	16 (44.4)	42 (44.2)	
Retired	18 (50.0)	48 (50.5)	
Others	2 (5.6)	5 (5.3)	
Married/living together (*n*, %) Missing value	24 (66.7)	50 (52.6) 1	0.235
Smoking (*n*, %)			0.530
Never	9 (25.0)	27 (28.4)	
Stopped smoking	12 (33.3)	39 (41.1)	
Smoker	15 (41.7)	29 (30.5)	
Number of days drinking Missing value	4 (1–8) 9	5 (2–11) 28	0.191
Number of standard glasses each day of drinking Missing value	13	36	0.991
Never drinking	3 (8.3)	8 (8.5)	
1	4 (11.1)	11 (11.6)	
2	13 (36.1)	28 (29.5)	
3	2 (5.6)	8 (8.4)	
4	1 (2.8)	3 (3.2)	
≥5	0	1 (1.7)	

Values are presented as a number (percent) or median (interquartile range). Fisher’s exact test or Mann–Whitney U test was used. A *p*-value < 0.05 was considered statistically significant.

**Table 8:** Comorbidity and symptoms depending on corticosteroid use.

	Corticosteroids *N* = 36	No Corticosteroids *N* = 95	*p*-Value
Comorbidity (*n*, %)			
Asthma and/or bronchitis Missing value	10 (27.8) 4	12 (12.6) 15	0.147
Diabetes Missing value	3 (8.3) 4	8 (8.4) 15	0.886
Cancer Missing value	3 (8.3) 4	8 (8.7) 19	0.663
Celiac disease Missing value	2 (5.6) 5	7 (7.4) 22	0.721
Gastric ulcer Missing value	6 (16.7) 4	15 (16.3) 16	0.953
Hypertension Missing value	13 (36.1) 2	28 (29.5) 12	0.484
Rheumatoid arthritis Missing value	10 (27.8) 2	16 (16.8) 14	0.194
Thyroid disease Missing value	6 (16.7) 4	24 (25.5) 11	0.597
Drug treatment (*n*, %)			
Proton pump inhibitors	12 (33.3)	22 (23.2)	0.267
Selective serotonin reuptake inhibitors	10 (27.8)	16 (16.8)	0.219
Statins	6 (16.7)	20 (21.1)	0.633
Thyroid hormones	6 (16.7)	22 (23.2)	0.482
Gastrointestinal symptoms (mm)			
Abdominal pain 5 (1–15)	36 (19–55)	35 (8–53)	0.478
Diarrhea 3 (0–10)	52 (24–80)	43 (16–70)	0.264
Constipation 9 (1–22)	7 (3–19)	8 (2–28)	0.877
Bloating and flatulence 14 (1–29)	62 (44–79)	53 (19–71)	0.036
Vomiting and nausea 2 (0–3)	8 (2–40)	8 (1–23)	0.766
Intestinal symptom’s influence on daily life 2 (0–18)	45 (21–80)	40 (16–77)	0.437
Psychological well-being 4 (0–16)	30 (9–57)	23 (10–46)	0.208

Gastrointestinal symptoms were assessed by the Visual Analog Scale for Irritable Bowel Syndrome (VAS-IBS) on a scale of 0–100 mm, where 0 represents no symptoms and 100 represents maximal symptoms [24]. Reference values are from healthy controls [25]. Values are presented as a number (percent) or median (interquartile range). Fisher’s exact test or Mann–Whitney U test was used. A *p*-value < 0.05 was considered statistically significant.

**Table 9:** Basal characteristics depending on smoking habits.

	Never Smoked *N* = 36	Former Smoker *N* = 51	Present Smoker *N* = 44	*p*-Value
Age (year)	65.3 (56.2–70.1)	65.4 (61.7–68.2)	60.0 (55.0–64.2)	0.001
BMI (kg/m^2^) Missing value	25.8 (24.1–29.9) 16	24.8 (23.0–28.7) 17	24.1 (21.7–26.6) 10	0.164
Persistent MC (year)	23 (63.9)	19 (37.3)	31 (70.5)	0.003
CC/LC (year)	20 (55.6)/16 (44.4)	27 (52.9)/24 (47.1)	29 (65.9)/15 (34.1)	0.394
IBS (*n*, %)	18 (50)	22 (43.1)	28 (63.6)	0.134
Disease duration (year) Missing value	6 (3–11) 5	6 (3–12) 3	11 (6–21) 3	0.017
University education (*n*, %) Missing value	13 (36.1) 1	15 (29.4) 1	16 (36.4) 2	0.856
Occupation (*n*, %)				0.004
Employed	14 (38.9)	20 (39.2)	24 (54.5)	
Retired	21 (58.3)	31 (60.8)	14 (31.8)	
Others	1 (2.8)	0	6 (13.6)	
Married/living together (*n*, %) Missing value	24 (66.7)	31 (60.8)	19 (43.2) 1	0.111
Number of days drinking Missing value	4 (1–8) 11	5 (3–10) 11	4 (1–10) 15	0.335
Number of standard glasses each day of drinking Missing value	14	16	19	0.505
Never drinking	3 (8.4)	2 (3.9)	6 (13.6)	
1	6 (16.7)	5 (9.8)	4 (9.1)	
2	11 (30.6)	19 (37.3)	11 (25.0)	
3	1 (2.8)	6 (11.8)	3 (6.8)	
4	1 (2.8)	2 (3.9)	1 (2.3)	
≥5	0	1 (2.0)	0	

The Kruskal–Wallis test was used. A *p*-value < 0.05 was considered statistically significant.

**Table 10:** Comobidity and symptoms depending on smoking habits.

	Never *N* = 36	Former *N* = 51	Present *N* = 44	*p*-Value
Comorbidity (*n*, %)				
Asthma and/or bronchitis Missing value	6 (16.7) 5	7 (13.7) 12	9 (20.5) 2	0.114
Diabetes Missing value	5 (13.9) 4	4 (7.8) 12	2 (4.5) 3	0.075
Cancer Missing value	1 (2.8) 5	4 (8.2) 14	6 (14.0) 4	0.087
Celiac disease Missing value	3 (9.7) 5	6 (16.7) 15	0 7	0.019
Gastric ulcer Missing value	7 (20.0) 6	7 (14.3) 11	7 (15.9) 3	0.507
Hypertension Missing value	9 (25.0) 4	20 (39.2) 7	12 (27.3) 3	0.350
Rheumatoid arthritis Missing value	7 (19.4) 4	9 (17.6) 10	10 (22.7) 2	0.265
Thyroid disease Missing value	5 (13.9) 5	17 (34.0) 8	8 (18.2) 2	0.053
Drug treatment (*n*, %)				
Corticosteroid treatment	9 (25.0)	12 (23.5)	15 (34.1)	0.530
Proton pump inhibitors	11 (30.6)	12 (23.5)	11 (25.0)	0.721
Selective serotonin reuptake inhibitors	8 (22.2)	7 (13.7)	11 (25.0)	0.344
Statins	5 (13.9)	14 (27.5)	7 (15.9)	0.238
Thyroid hormones	6 (16.7)	14 (27.5)	8 (18.2)	0.424
Gastrointestinal symptoms (mm)				
Abdominal pain 5 (1–15)	36 (14–50)	31 (6–54)	36 (13–58)	0.488
Diarrhea 3 (0–10)	43 (16–59)	39 (14–73)	55 (29–80)	0.084
Constipation 9 (1–22)	7 (2–23)	8 (2–29)	7 (3–18)	0.968
Bloating and flatulence 14 (1–29)	51 (18–60)	50 (24–75)	63 (27–78)	0.166
Vomiting and nausea 2 (0–3)	6 (0–20)	8 (2–23)	10 (3–44)	0.329
Intestinal symptom’s influence on daily life 2 (0–18)	32 (15–70)	36 (13–71)	66 (29–88)	0.025
Psychological well-being 4 (0–16)	17 (5–33)	21 (10–38)	34 (10–61)	0.072

Gastrointestinal symptoms were assessed by the Visual Analog Scale for Irritable Bowel Syndrome (VAS-IBS) on a scale of 0–100 mm, where 0 represents no symptoms and 100 represents maximal symptoms [24]. Reference values are from healthy controls [25]. Values are presented as a number (percent) or median (interquartile range). Fisher’s exact test or Mann–Whitney U test was used. A *p*-value < 0.05 was considered statistically significant.

## Data Availability

The data presented in this study are available upon request from the corresponding author due to Swedish ethical rules.

## Supplementary Materials

The following supporting information can be downloaded at https://www.mdpi.com/article/10.3390/metabo14060303/s1: Table S1: The differences in relative metabolomic concentrations between collagenous colitis and lymphocytic colitis; Table S2: The differences in relative metabolomic concentrations between one episode of microscopic colitis or refractory microscopic colitis; Table S3: The differences in relative metabolomic concentrations between patients with IBS-like symptoms and those without; Table S4: The differences in relative metabolomic concentrations between users of corticosteroids and non-users; Table S5: The differences in relative metabolomic concentrations between smokers and non-smokers; Table S6: The differences in relative metabolomic concentrations between one episode of collagenous colitis or refractory collagenous colitis; Table S7: The differences in relative metabolomic concentrations between patients with IBS-like symptoms or those without in collagenous colitis; Table S8: The differences in relative metabolomic concentrations between users of corticosteroids or non-users in collagenous colitis; Table S9: The differences in relative metabolomic concentrations between smokers and non-smokers in collagenous colitis; Supplementary Figure S1: Principal component analysis divided by one episode of CC or refractory collagenous colitis (CC); Supplementary Figure S2: Principal component analysis divided by the presence of irritable bowel syndrome (IBS)-like symptoms or not; Supplementary Figure S3: Principal component analysis divided by the use of corticosteroids or not; Supplementary Figure S4: Principal component analysis divided by smoking or non-smoking.
