# Supplementary material for: The Metabolomic Profile of Microscopic Colitis Is Affected by Smoking but Not Histopathological Diagnosis, Clinical Course, Symptoms, or Treatment
Source: Metabolites. 2024 May 27;14(6):303. doi: 10.3390/metabo14060303 (PMC11205623; doi:10.3390/metabo14060303)
Supplement: Supplementary file 1 [file metabolites-14-00303-s001.zip › metabolites-3014461-supplementary.pdf]

Supplementary Table S1. The differences in relative metabolomic concentrations between collagenous colitis and lymphocytic colitis

variable	mean difference	p_value	adj_p_value
Indolelactic acid	-0.24	0.003	0.238
Indole-3-propionic acid	-0.74	0.003	0.238
LysoPE(22:5(4Z,7Z,10Z,13Z,16Z)/0:0)	-0.25	0.003	0.238
LysoPC(0:0/15:0)	-0.33	0.004	0.238
Tiglylcarnitine	-0.33	0.004	0.238
LysoPC(15:0/0:0)	-0.26	0.004	0.238
LysoPC(14:1(9Z)/0:0)	-0.36	0.006	0.291
Isovalerylcarnitine	-0.23	0.008	0.291
Phenylalanyltryptophan / Tryptophyl-Phenylalanine	-0.31	0.009	0.291
Uracil	-0.24	0.011	0.291
Uridine/Pseudouridine	-0.21	0.012	0.291
LysoPC(14:0/0:0)	-0.3	0.014	0.291
LysoPE(0:0/16:0)	-0.2	0.015	0.291
Indole-3-carboxaldehyde	-0.29	0.016	0.291
Prolylhydroxyproline / Pyroglutamylvaline	0.24	0.017	0.291
Propionylcarnitine	-0.2	0.019	0.291
L-Isoleucine	-0.13	0.019	0.291
LysoPC(0:0/14:0)	-0.3	0.019	0.291
LysoPE(18:3(9Z,12Z,15Z)/0:0)	-0.28	0.019	0.291
Cortisone	-0.31	0.021	0.291
LysoPE(18:2(9Z,12Z)/0:0)	-0.24	0.021	0.291
LysoPE(18:1(9Z)/0:0)	-0.23	0.022	0.291
3-Hydroxyisovaleric acid	-0.17	0.022	0.291
alpha-Ketoisovaleric acid	-0.14	0.022	0.291
Kynurenic acid	-0.27	0.023	0.291
LysoPE(0:0/18:1(9Z))	-0.23	0.024	0.291
Tetradecanedioic acid	-0.41	0.025	0.291
LysoPC(12:0/0:0)	-0.42	0.026	0.291
LysoPE(22:4(7Z,10Z,13Z,16Z)/0:0)	-0.17	0.026	0.291
N-Acetyl-L-alanine	-0.1	0.028	0.301
LysoPE(20:5(5Z,8Z,11Z,14Z,17Z)/0:0)	-0.25	0.03	0.308
LysoPE(16:0/0:0)	-0.2	0.03	0.308
3-Hydroxyisovalerylcarnitine	-0.22	0.032	0.317
2-Methoxybenzoic acid	-0.18	0.034	0.318
Valerylcarnitine	-0.24	0.034	0.318
Pimelic acid / 3-Methyladipic acid	-0.1	0.036	0.321
Phenylalanylproline	-0.23	0.037	0.321
Ketoleucine	-0.18	0.038	0.321
LysoPC(18:0(OH)/0:0)	-0.2	0.039	0.323
Niacinamide	-0.24	0.04	0.323
Pantothenic acid	-0.25	0.042	0.334
Glutarylcarnitine	-0.19	0.044	0.34

LysoPE(0:0/18:2(9Z,12Z))	-0.23	0.045	0.343
1-Methyladenosine	-0.14	0.047	0.35
Taurochenodesoxycholic acid	-0.22	0.053	0.369
LysoPE(0:0/18:3(6Z,9Z,12Z))	-0.21	0.053	0.369
L-Palmitoylcarnitine	-0.25	0.054	0.369
Sebacic acid	-0.17	0.054	0.369
LysoPC(18:3(9Z,12Z,15Z)/0:0)	-0.21	0.058	0.386
LysoPE(18:0/0:0)	-0.18	0.06	0.39
LysoPE(0:0/18:0)	-0.16	0.062	0.392
Indoleacetic acid	-0.24	0.065	0.392
LysoPC(0:0/18:0(OH))	-0.23	0.066	0.392
Isobutyryl-L-carnitine	-0.25	0.067	0.392
Indoleacetaldehyde	-0.1	0.067	0.392
Suberic acid	-0.1	0.068	0.392
Dodecanedioic acid	-0.16	0.08	0.443
Adenosine	0.35	0.08	0.443
L-Tryptophan	-0.08	0.081	0.443
3-Hydroxyoctanoic acid	-0.21	0.082	0.443
Salicylic acid	0.7	0.083	0.443
Chenodeoxycholic acid	0.46	0.086	0.45
LysoPC(17:0/0:0)	-0.21	0.089	0.453
LysoPE(0:0/20:5(5Z,8Z,11Z,14Z,17Z))	-0.16	0.089	0.453
PGD2 (pg/ml)	-0.42	0.093	0.464
LysoPC(20:0/0:0)	-0.2	0.096	0.464
TxB2 (pg/ml)	-0.8	0.096	0.464
Ursodeoxycholic acid	-0.31	0.099	0.464
Azelaic acid	-0.19	0.1	0.464
Adipic acid	-0.13	0.101	0.464
LysoPC(0:0/17:0)	-0.18	0.101	0.464
Taurocholic acid	-0.31	0.104	0.465
LysoPC(0:0/16:0)	-0.11	0.106	0.465
Cholic acid	0.39	0.107	0.465
Stearoylcarnitine	-0.22	0.111	0.465
Octadecanedioic acid	-0.16	0.112	0.465
LysoPC(19:0/0:0)	-0.24	0.114	0.465
N-Acetylserine	-0.13	0.114	0.465
(+-)9-HETE (pg/ml)	0.38	0.116	0.465
alpha-Chaconine	-0.53	0.116	0.465
LysoPC(0:0/18:3)	-0.18	0.118	0.465
LysoPC(18:1(9Z)/0:0)	-0.07	0.119	0.465
L-Phenylalanine	-0.04	0.12	0.465
(S)-3-Hydroxyisobutyric acid	-0.18	0.122	0.465
Oleamide	-0.2	0.123	0.465
LysoPC(18:2(9Z,12Z)/0:0)	-0.07	0.123	0.465
Isoleucyl-Isoleucine	-0.15	0.127	0.472
Tetradecanoylcarnitine	-0.22	0.128	0.472
LysoPC(18:4(6Z,9Z,12Z,15Z)/0:0)	-0.22	0.133	0.477
Deoxycholic acid glycine conjugate	-0.33	0.136	0.477

D-Phenyllactic acid	-0.2	0.136	0.477
Succinyladenosine	-0.12	0.136	0.477
N-Acetyl-L-methionine	-0.1	0.137	0.477
D-Glucoheptose	0.11	0.141	0.486
Cinnamoylglycine	-0.37	0.144	0.491
LysoPC(0:0/18:2(9Z,12Z))	-0.13	0.153	0.512
N-gamma-Glutamylglutamine	0.1	0.153	0.512
Linoleylcarnitine	-0.17	0.156	0.512
3-Hydroxytetradecanoic acid	-0.1	0.156	0.512
Paraxanthine	0.44	0.159	0.513
Isoleucyl-Leucine	-0.15	0.159	0.513
N2,N2-Dimethylguanosine	-0.13	0.164	0.514
Caffeine	0.41	0.165	0.514
LysoPC(0:0/18:0)	-0.16	0.167	0.514
2-Hydroxycaproic acid	-0.12	0.167	0.514
LysoPC(18:0/0:0)	-0.07	0.169	0.514
Bilirubin	-0.18	0.17	0.514
2-Hydroxydecanoate	-0.16	0.171	0.514
N-Formyl-L-methionine	-0.08	0.175	0.52
LysoPC(16:0/0:0)	-0.03	0.178	0.524
9(10)-EpOME (pg/ml)	0.26	0.184	0.524
6-trans-LTB4 (pg/ml)	0.44	0.184	0.524
11(12)-EpETrE (pg/ml)	0.18	0.185	0.524
LysoPE(0:0/20:4(5Z,8Z,11Z,14Z))	-0.1	0.186	0.524
LysoPC(22:5(4Z,7Z,10Z,13Z,16Z)/0:0)	-0.15	0.186	0.524
5-Hydroxyindoleacetic acid	-0.11	0.187	0.524
3-Methylglutarylcarnitine	-0.17	0.194	0.538
L-Kynurenine	-0.11	0.2	0.552
Cortisol / hydrocortisone	-0.22	0.202	0.552
2-Hydroxylauroylcarnitine	-0.19	0.209	0.56
gamma-Glutamyltryptophan	-0.07	0.209	0.56
2-Hydroxymyristoylcarnitine	-0.19	0.21	0.56
Dodecanoylcarnitine	-0.21	0.214	0.566
Succinic acid	-0.07	0.216	0.566
L-Leucine	-0.05	0.219	0.567
L-Octanoylcarnitine	-0.21	0.221	0.567
PGE2 (pg/ml)	-0.47	0.222	0.567
Decanoylcarnitine	-0.22	0.223	0.567
Oxoglutaric acid	-0.08	0.227	0.567
Pyroglutamic acid	0.06	0.231	0.567
LysoPE(O-18:0/0:0)	-0.15	0.233	0.567
Quinic acid	0.25	0.233	0.567
12-HETE (pg/ml)	0.25	0.234	0.567
N-(3-acetamidopropyl)pyrrolidin-2-one	-0.11	0.234	0.567
4-Pyridoxic acid	-0.26	0.236	0.567
Hydroxyoctanoic acid	-0.16	0.237	0.567
Methylguanosine	-0.07	0.244	0.576

Phenylalanylglutamic acid / gamma-Glutamylphenylalanine	-0.07	0.246	0.576
Theophylline	0.28	0.247	0.576
LysoPE(0:0/22:6(4Z,7Z,10Z,13Z,16Z,19Z))	-0.09	0.249	0.576
Betaine	-0.04	0.25	0.576
LysoPC(0:0/19:0)	-0.12	0.254	0.581
5-oxo-ETE (pg/ml)	0.23	0.257	0.583
Citrulline	0.09	0.261	0.588
Citric acid	0.04	0.271	0.588
Phenylalanylisoleucine	-0.09	0.271	0.588
12(13)-EpOME (pg/ml)	0.28	0.273	0.588
3-Methoxytyrosine	-0.07	0.274	0.588
Levonorgestrel	-0.16	0.274	0.588
12(S)-HEPE (pg/ml)	0.24	0.275	0.588
3beta,7alpha-Dihydroxy-5-cholestenoate	-0.12	0.277	0.588
Gamma-linolenyl arnitine	-0.13	0.278	0.588
12-Hydroxystearic acid	0.12	0.279	0.588
5'-Methylthioadenosine	-0.08	0.283	0.588
8 9-DiHETrE (pg/ml)	-0.11	0.283	0.588
gamma-Glutamylleucine	-0.12	0.284	0.588
LysoPE(20:4(5Z,8Z,11Z,14Z)/0:0)	-0.08	0.285	0.588
Butyrylcarnitine	-0.12	0.286	0.588
Hexadecanedioic acid	-0.12	0.29	0.59
gamma-Glutamylalanine	0.11	0.291	0.59
Lithocholic acid glycine conjugate	-0.13	0.292	0.59
2-Hydroxy-3-methylbutyric acid	-0.13	0.3	0.598
Docosapentaenoylcarnitine	-0.12	0.3	0.598
Serotonin	0.79	0.304	0.599
L,L-Cyclo(leucylprolyl)	0.24	0.304	0.599
Isocitric acid	0.05	0.31	0.608
Inosine	0.14	0.314	0.611
AICAR	-0.09	0.319	0.617
3-Hydroxybutyrylcarnitine	-0.35	0.322	0.617
Indoxyl sulfate	-0.14	0.324	0.617
5(s)6(R)-LXA4 (pg/ml)	0.21	0.325	0.617
LysoPC(20:2(11Z,14Z)/0:0)	-0.11	0.333	0.625
Lenticin	-0.38	0.334	0.625
LysoPE(O-16:0/0:0)	-0.13	0.335	0.625
3-Hydroxycapric acid	-0.12	0.344	0.634
Stearic acid	0.18	0.344	0.634
Capryloylglycine	-0.13	0.347	0.634
Pipecolic acid	-0.1	0.347	0.634
Phenylacetylglutamine	-0.19	0.353	0.639
2-Hydroxystearic acid	0.2	0.354	0.639
7-Methylguanine	-0.05	0.365	0.651
Thyroxine	-0.07	0.367	0.651
Undecanedioic acid	-0.06	0.368	0.651
Uric acid	-0.04	0.369	0.651

4-Hydroxyproline	0.06	0.374	0.657
Palmitic acid	0.14	0.381	0.666
Sphingosine 1-phosphate	-0.06	0.389	0.676
1-Aminocyclopropanecarboxylic acid	0.05	0.395	0.683
9 10-DiHOME (pg/ml)	0.19	0.398	0.683
LysoPC(0:0/20:5)	-0.12	0.399	0.683
Glyceric acid	-0.07	0.408	0.694
Deoxycholic acid	-0.19	0.411	0.695
LysoPC(0:0/18:1(9Z))	-0.04	0.413	0.696
9,12-Hexadecadienoylcarnitine	-0.13	0.417	0.697
L-Sorbose	-0.16	0.418	0.697
3-Hydroxyoctadecenoylcarnitine	-0.14	0.426	0.703
11 12-DiHETrE (pg/ml)	-0.09	0.426	0.703
2-Hydroxy-3-methylpentanoic acid	-0.09	0.436	0.716
12 13-DiHOME (pg/ml)	0.16	0.441	0.721
Phenylalanylphenylalanine	-0.07	0.446	0.725
9 10 13-TriHOME (pg/ml)	-0.14	0.454	0.726
LysoPC(16:0(OH)/0:0)	-0.08	0.457	0.726
LysoPE(22:6(4Z,7Z,10Z,13Z,16Z,19Z)/0:0)	-0.06	0.457	0.726
(R)-3-Hydroxy-hexadecanoic acid	0.08	0.459	0.726
8(9)-EpETrE (pg/ml)	0.12	0.459	0.726
LysoPC(0:0/22:4)	-0.06	0.468	0.726
3-(3-Hydroxyphenyl)-3-hydroxypropanoic acid / 4-Hydroxyphenyllactic acid	-0.06	0.473	0.726
Glycoursodeoxycholic acid	-0.16	0.474	0.726
2-Hydroxyadipic acid / 3-Hydroxyadipic acid / 3-Hydroxymethylglutaric acid	-0.03	0.475	0.726
Malic acid	-0.05	0.475	0.726
4-Coumaryl alcohol	-0.21	0.475	0.726
14 15-DiHETrE (pg/ml)	-0.06	0.476	0.726
3-Methylcrotonylglycine / Tiglylglycine	-0.1	0.479	0.726
3-Hydroxydodecanoic acid	-0.09	0.479	0.726
Oleoylcarnitine	-0.09	0.482	0.726
Hippuric acid	-0.15	0.483	0.726
LysoPC(16:1(9Z)/0:0)	-0.07	0.485	0.726
LTB4 (pg/ml)	0.29	0.489	0.728
LysoPE(P-18:0/0:0)	-0.1	0.491	0.729
LysoPC(20:1(11Z)/0:0)	-0.08	0.504	0.744
Xanthine	0.07	0.508	0.745
LysoPC(20:5(5Z,8Z,11Z,14Z,17Z)/0:0)	-0.08	0.509	0.745
L-Acetylcarnitine	-0.06	0.517	0.752
(5Z,8Z,11Z,14Z,17Z)-Icosa-5,8,11,14,17-pentaenoylcarnitine	-0.08	0.52	0.752
8(S)-HETE (pg/ml)	0.11	0.521	0.752
Icosa-8,11,14-trienoylcarnitine	-0.07	0.532	0.763
Glutaric acid	-0.05	0.533	0.763
15(S)-HETrE (pg/ml)	-0.12	0.537	0.765

Guanosine	0.09	0.545	0.767
LysoPC(0:0/20:2)	-0.04	0.549	0.767
cis-4-Decenoylcarnitine	-0.1	0.549	0.767
(2E)-3-(4-hydroxy-3-methoxyphenyl)prop-2-enal	-0.11	0.55	0.767
Hexanoylcarnitine	-0.09	0.553	0.767
8-iso-PGE2 (pg/ml)	-0.22	0.555	0.767
L-Proline	0.04	0.556	0.767
gamma-Glutamylisoleucine	-0.08	0.557	0.767
3, 5-Tetradecadiencarnitine	-0.1	0.559	0.767
2-Octenoylcarnitine	-0.09	0.562	0.767
Leucyl-Aspartate / gamma-Glutamylvaline	-0.08	0.579	0.787
15-oxo-ETE (pg/ml)	0.1	0.588	0.793
Cervonylcarnitine	-0.07	0.588	0.793
Oleic acid / Elaidic acid	0.1	0.59	0.793
MG(18:1(9Z)/0:0/0:0)	0.07	0.595	0.796
Arachidonoylcarnitine	-0.06	0.608	0.808
LysoPC(O-16:0/0:0)	-0.06	0.609	0.808
4-Acetamidobutanoic acid	-0.02	0.616	0.814
LysoPC(20:4(5Z,8Z,11Z,14Z)/0:0)	0.03	0.62	0.815
(+-)11-HETE (pg/ml)	0.1	0.622	0.815
Asymmetric dimethylarginine	0.04	0.629	0.817
L-Tyrosine	0.03	0.63	0.817
gamma-Glutamylmethionine	-0.05	0.631	0.817
Methylmalonylcarnitine	-0.03	0.633	0.817
Trigonelline	0.1	0.639	0.818
LysoPE(P-16:0/0:0)	-0.06	0.64	0.818
3-Hydroxybutyric acid	-0.11	0.641	0.818
Bis(2-ethylhexyl)phthalate	-0.02	0.65	0.819
20-HETE (pg/ml)	0.06	0.652	0.819
5-HETE (pg/ml)	0.13	0.653	0.819
Creatine	0.06	0.654	0.819
Hypoxanthine	0.06	0.659	0.819
LysoPC(P-18:0/0:0)	-0.06	0.66	0.819
Glycocholic acid	-0.1	0.66	0.819
Arachidonic acid	0.06	0.679	0.839
LysoPC(20:3(8Z,11Z,14Z)/0:0)	-0.03	0.686	0.839
LysoPC(0:0/22:6)	-0.05	0.689	0.839
LysoPC(0:0/20:4)	-0.03	0.693	0.839
2-Hydroxymyristic acid	-0.05	0.693	0.839
Myristoleoylcarnitine	-0.07	0.696	0.839
LysoPC(22:4(7Z,10Z,13Z,16Z)/0:0)	-0.03	0.698	0.839
S-Adenosylhomocysteine	0.05	0.698	0.839
MG(18:2(9Z,12Z)/0:0/0:0)	-0.04	0.701	0.839
9 12 13-TriHOME (pg/ml)	-0.11	0.702	0.839
2,3,4,5,6,7-Hexahydroxyheptanoic acid	-0.02	0.706	0.84
Creatinine	-0.02	0.72	0.854

1,11-Undecanedicarboxylic acid	-0.03	0.729	0.861
Eicosapentaenoic acid	-0.05	0.731	0.861
12-OxoETE (pg/ml)	-0.06	0.735	0.861
Homo-L-arginine	-0.03	0.737	0.861
9-Hexadecenoylcarnitine	-0.05	0.743	0.865
2,4-Dihydroxybenzoic acid / 2-Pyrocatechuic acid	-0.06	0.747	0.867
Trimethylamine N-oxide	-0.05	0.758	0.876
2-Hydroxyhexadecanoic acid	0.04	0.761	0.877
Citraconic acid	0.05	0.777	0.887
LysoPC(0:0/20:1)	-0.02	0.78	0.887
LysoPC(0:0/20:3)	-0.03	0.78	0.887
(11Z)-Eicoseneoylcarnitine	-0.04	0.781	0.887
gamma-Glutamyltyrosine	-0.02	0.81	0.917
13-HODE (pg/ml)	0.05	0.814	0.918
Epinephrine	0.02	0.82	0.921
5 6-DiHETrE (pg/ml)	-0.03	0.823	0.921
4-Hydroxybenzoic acid	0.05	0.824	0.921
5(6)-EpETrE (pg/ml)	0.03	0.834	0.928
(7Z,10Z,13Z,16Z)-Docosatetraenoylcarnitine	-0.02	0.838	0.93
Cytosine	-0.03	0.841	0.93
Linoleic acid	0.03	0.844	0.93
13-oxo-ODE (pg/ml)	0.04	0.854	0.933
Hexose	0.02	0.854	0.933
alpha-Dimorphecolic acid	-0.02	0.856	0.933
15-HETE (pg/ml)	0.03	0.86	0.934
Acetoacetic acid	0.03	0.869	0.938
6-keto-PGF1a (pg/ml)	-0.07	0.87	0.938
Acetaminophen	-0.11	0.872	0.938
(±)-2-Hydroxy-4-(methylthio)butanoic acid	0.01	0.888	0.952
Docosapentaenoic acid (22n-3)	0.03	0.893	0.954
Threonic acid	0.02	0.9	0.959
Theobromine	-0.02	0.917	0.974
Docosahexaenoic acid	0.01	0.925	0.979
LysoPC(0:0/16:1)	-0.01	0.935	0.985
Guanine	0.01	0.937	0.985
LysoPC(0:0/22:5)	-0.01	0.941	0.986
Chenodeoxycholic acid glycine conjugate	-0.01	0.951	0.988
4-Hydroxyhippuric acid	-0.01	0.959	0.988
gamma-Aminobutyric acid	0	0.959	0.988
D-Leucic acid	0	0.962	0.988
L-Methionine	0	0.963	0.988
17-HDOHE (pg/ml)	0.01	0.964	0.988
9(S)-HODE (pg/ml)	0.01	0.966	0.988
alpha-Linolenic acid	0.01	0.968	0.988
LysoPC(22:6(4Z,7Z,10Z,13Z,16Z,19Z)/0:0)	0	0.97	0.988

trans-2-Dodecenoylcarnitine	0	0.98	0.995
Itaconic acid	0	0.989	0.997
3-Carboxy-4-methyl-5-propyl-2-furanpropionic acid	0	0.989	0.997
2',4'-Dihydroxyacetophenone (Resorcinol monoacetate)	0	0.991	0.997
3a,7b,12a-Trihydroxyoxocholanyl-Glycine	0	0.997	0.998
L-Lysine	0	0.998	0.998

Supplementary Table S2. The differences in relative metabolomic concentrations between one episode of microscopic colitis or refractory microscopic colitis

variable	mean difference	p_value	adj_p_value
Deoxycholic acid	-0.63	0.008	0.543
Hydroxyoctanoic acid	-0.33	0.012	0.543
3-Hydroxybutyric acid	-0.58	0.013	0.543
Phenylacetylglutamine	-0.47	0.014	0.543
Theobromine	0.53	0.016	0.543
Isovalerylcarnitine	-0.21	0.017	0.543
N-Acetyl-L-alanine	-0.1	0.018	0.543
Succinic acid	-0.14	0.018	0.543
Arachidonoylcarnitine	-0.26	0.018	0.543
20-HETE (pg/ml)	-0.29	0.024	0.543
LysoPC(22:4(7Z,10Z,13Z,16Z)/0:0)	-0.18	0.027	0.543
3-Methoxytyrosine	-0.15	0.027	0.543
MG(18:1(9Z)/0:0/0:0)	0.29	0.027	0.543
Linoleylcarnitine	-0.26	0.027	0.543
1,11-Undecanedicarboxylic acid	0.18	0.031	0.543
(7Z,10Z,13Z,16Z)-Docosatetraenoylcarnitine	-0.23	0.031	0.543
Pimelic acid / 3-Methyladipic acid	-0.1	0.031	0.543
Gamma-linolenyl arnitine	-0.26	0.032	0.543
Icosa-8,11,14-trienoylcarnitine	-0.23	0.032	0.543
LysoPC(20:4(5Z,8Z,11Z,14Z)/0:0)	-0.13	0.034	0.547
Deoxycholic acid glycine conjugate	-0.49	0.04	0.56
4-Acetamidobutanoic acid	-0.09	0.044	0.56
3-Hydroxyoctanoic acid	-0.24	0.044	0.56
LysoPE(20:4(5Z,8Z,11Z,14Z)/0:0)	-0.14	0.045	0.56
cis-4-Decenoylcarnitine	-0.31	0.046	0.56
Tetradecanoylcarnitine	-0.29	0.047	0.56
L-Acetylcarnitine	-0.18	0.049	0.56
Ursodeoxycholic acid	-0.38	0.049	0.56
3-Methylglutarylcarnitine	-0.26	0.05	0.56
Undecanedioic acid	0.13	0.052	0.56
Propionylcarnitine	-0.16	0.056	0.586
Indole-3-propionic acid	-0.47	0.063	0.622
alpha-Linolenic acid	-0.36	0.063	0.622
12-Hydroxystearic acid	0.21	0.065	0.623
Bis(2-ethylhexyl)phthalate	-0.1	0.069	0.631
AICAR	-0.16	0.075	0.631
3, 5-Tetradecadiencarnitine	-0.29	0.08	0.631
(R)-3-Hydroxy-hexadecanoic acid	0.19	0.084	0.631
L-Palmitoylcarnitine	-0.22	0.087	0.631
Tetradecanedioic acid	-0.29	0.093	0.631
Stearic acid	0.3	0.094	0.631
13-HODE (pg/ml)	-0.32	0.094	0.631
alpha-Ketoisovaleric acid	-0.1	0.095	0.631
9-Hexadecenoylcarnitine	-0.24	0.097	0.631

trans-2-Dodecenoylcarnitine	-0.29	0.098	0.631
LysoPC(0:0/20:4)	-0.14	0.099	0.631
Uridine/Pseudouridine	-0.14	0.099	0.631
9,12-Hexadecadienoylcarnitine	-0.26	0.102	0.631
Paraxanthine	0.49	0.102	0.631
Dodecanoylcarnitine	-0.26	0.103	0.631
Malic acid	-0.1	0.104	0.631
Docosapentaenoylcarnitine	-0.18	0.104	0.631
Tiglylcarnitine	-0.18	0.106	0.631
2-Octenoylcarnitine	-0.25	0.112	0.631
Isobutyryl-L-carnitine	-0.22	0.113	0.631
(5Z,8Z,11Z,14Z,17Z)-Icosa-5,8,11,14,17-pentaenoylcarnitine	-0.2	0.114	0.631
LysoPC(0:0/22:4)	-0.13	0.115	0.631
LysoPE(0:0/22:6(4Z,7Z,10Z,13Z,16Z,19Z))	-0.12	0.117	0.631
4-Hydroxyhippuric acid	-0.22	0.121	0.631
3-(3-Hydroxyphenyl)-3-hydroxypropanoic acid / 4-Hydroxyphenyllactic acid	-0.12	0.122	0.631
Prolylhydroxyproline / Pyroglutamylvaline	0.16	0.124	0.631
Butyrylcarnitine	-0.18	0.124	0.631
Bilirubin	-0.2	0.124	0.631
Oleoylcarnitine	-0.2	0.127	0.631
(S)-3-Hydroxyisobutyric acid	-0.17	0.128	0.631
3-Hydroxycapric acid	-0.2	0.129	0.631
Uric acid	-0.07	0.131	0.631
11(12)-EpETrE (pg/ml)	-0.2	0.135	0.631
alpha-Dimorphecolic acid	-0.18	0.141	0.631
gamma-Aminobutyric acid	-0.09	0.142	0.631
L-Octanoylcarnitine	-0.25	0.142	0.631
Adipic acid	-0.11	0.145	0.631
Methylmalonylcarnitine	-0.1	0.145	0.631
Kynurenic acid	-0.16	0.147	0.631
Acetoacetic acid	-0.27	0.148	0.631
3-Hydroxyoctadecenoylcarnitine	-0.25	0.149	0.631
Theophylline	0.34	0.149	0.631
Decanoylcarnitine	-0.25	0.154	0.64
Cortisone	-0.19	0.16	0.645
2-Hydroxy-3-methylbutyric acid	-0.18	0.16	0.645
2-Hydroxymyristoylcarnitine	-0.21	0.163	0.645
Hippuric acid	-0.28	0.164	0.645
Cervonylcarnitine	-0.17	0.165	0.645
9(10)-EpOME (pg/ml)	-0.28	0.167	0.646
3a,7b,12a-Trihydroxyoxocholanyl-Glycine	0.2	0.17	0.651
Ketoleucine	-0.11	0.178	0.667
Myristoleoylcarnitine	-0.24	0.179	0.667
Serotonin	1.02	0.183	0.676
L-Sorbose	-0.24	0.185	0.676
2-Hydroxylauroylcarnitine	-0.19	0.189	0.68

9(S)-HODE (pg/ml)	-0.27	0.19	0.68
Phenylalanylproline	-0.14	0.196	0.692
11 12-DiHETrE (pg/ml)	-0.13	0.208	0.722
N-(3-acetamidopropyl)pyrrolidin-2-one	-0.12	0.209	0.722
LysoPC(0:0/22:6)	-0.15	0.213	0.729
Docosahexaenoic acid	-0.17	0.22	0.74
Cinnamoylglycine	-0.3	0.221	0.74
L-Isoleucine	-0.07	0.228	0.753
gamma-Glutamylalanine	0.13	0.23	0.753
2-Hydroxyadipic acid / 3-Hydroxyadipic acid / 3-Hydroxymethylglutaric acid	-0.05	0.232	0.753
5'-Methylthioadenosine	-0.09	0.238	0.766
Uracil	-0.11	0.241	0.768
N-Formyl-L-methionine	-0.06	0.245	0.773
L,L-Cyclo(leucylprolyl)	0.25	0.249	0.778
Capryloylglycine	-0.16	0.253	0.778
3-Hydroxyisovalerylcarnitine	-0.12	0.254	0.778
LysoPC(0:0/22:5)	-0.09	0.26	0.789
Octadecanedioic acid	-0.1	0.263	0.792
2-Hydroxystearic acid	0.24	0.267	0.794
Glycoursodeoxycholic acid	-0.23	0.271	0.794
D-Leucic acid	0.08	0.274	0.794
Levonorgestrel	-0.16	0.276	0.794
LysoPE(P-16:0/0:0)	-0.14	0.28	0.794
Isoleucyl-Isoleucine	-0.1	0.286	0.794
2-Hydroxydecanoate	-0.12	0.291	0.794
N-Acetyl-L-methionine	-0.08	0.292	0.794
Indoxyl sulfate	-0.15	0.294	0.794
LysoPC(0:0/18:1(9Z))	0.06	0.296	0.794
LysoPC(0:0/20:3)	-0.1	0.3	0.794
LysoPE(0:0/20:4(5Z,8Z,11Z,14Z))	-0.08	0.301	0.794
Caffeine	0.29	0.304	0.794
LysoPC(20:3(8Z,11Z,14Z)/0:0)	-0.08	0.309	0.794
Palmitic acid	0.16	0.309	0.794
Inosine	0.14	0.312	0.794
LysoPC(22:6(4Z,7Z,10Z,13Z,16Z,19Z)/0:0)	-0.08	0.312	0.794
LysoPE(22:4(7Z,10Z,13Z,16Z)/0:0)	-0.07	0.315	0.794
Acetaminophen	-0.69	0.319	0.794
LysoPE(O-16:0/0:0)	-0.13	0.319	0.794
Indoleacetic acid	-0.12	0.321	0.794
LysoPE(22:6(4Z,7Z,10Z,13Z,16Z,19Z)/0:0)	-0.08	0.321	0.794
8 9-DiHETrE (pg/ml)	-0.1	0.323	0.794
Glutarylcarnitine	-0.09	0.324	0.794
N-Acetylserine	-0.08	0.325	0.794
2,4-Dihydroxybenzoic acid / 2-Pyrocatechuic acid	-0.17	0.333	0.803
2',4'-Dihydroxyacetophenone (Resorcinol monoacetate)	-0.32	0.341	0.803

LysoPE(O-18:0/0:0)	-0.12	0.342	0.803
Glutaric acid	0.07	0.345	0.803
3-Carboxy-4-methyl-5-propyl-2-furanpropionic acid	0.18	0.346	0.803
L-Phenylalanine	-0.03	0.347	0.803
Trigonelline	0.18	0.348	0.803
Pipecolic acid	-0.1	0.353	0.803
Linoleic acid	-0.14	0.354	0.803
Suberic acid	-0.05	0.355	0.803
5(6)-EpETrE (pg/ml)	-0.11	0.357	0.803
Threonic acid	-0.14	0.36	0.803
LysoPC(16:0/0:0)	-0.02	0.362	0.803
LysoPE(P-18:0/0:0)	-0.13	0.367	0.803
LysoPC(22:5(4Z,7Z,10Z,13Z,16Z)/0:0)	-0.1	0.375	0.803
12-OxoETE (pg/ml)	-0.14	0.38	0.803
Niacinamide	-0.09	0.383	0.803
4-Coumaryl alcohol	-0.24	0.383	0.803
LysoPC(17:0/0:0)	-0.11	0.384	0.803
PGE2 (pg/ml)	0.36	0.384	0.803
12 13-DiHOME (pg/ml)	-0.17	0.384	0.803
1-Methyladenosine	-0.06	0.387	0.803
Oleic acid / Elaidic acid	-0.16	0.387	0.803
S-Adenosylhomocysteine	-0.11	0.395	0.803
Guanosine	0.12	0.399	0.803
LysoPC(0:0/20:2)	-0.06	0.402	0.803
3beta,7alpha-Dihydroxy-5-cholestenoate	-0.09	0.406	0.803
Cortisol / hydrocortisone	-0.14	0.409	0.803
12(13)-EpOME (pg/ml)	-0.2	0.412	0.803
LysoPC(0:0/19:0)	-0.08	0.412	0.803
LTB4 (pg/ml)	-0.33	0.417	0.803
Glyceric acid	0.07	0.419	0.803
9 10-DiHOME (pg/ml)	-0.18	0.424	0.803
Oxoglutaric acid	-0.05	0.426	0.803
gamma-Glutamyltyrosine	-0.06	0.426	0.803
(2E)-3-(4-hydroxy-3-methoxyphenyl)prop-2-enal	0.14	0.429	0.803
14 15-DiHETrE (pg/ml)	-0.07	0.43	0.803
(±)-2-Hydroxy-4-(methylthio)butanoic acid	-0.07	0.431	0.803
Creatinine	-0.05	0.433	0.803
8-iso-PGE2 (pg/ml)	0.29	0.435	0.803
Indolelactic acid	-0.06	0.439	0.803
LysoPC(20:1(11Z)/0:0)	-0.09	0.439	0.803
3-Hydroxydodecanoic acid	-0.1	0.441	0.803
LysoPC(18:0/0:0)	-0.04	0.446	0.803
Citric acid	0.03	0.449	0.803
5(s)6(R)-LXA4 (pg/ml)	0.15	0.45	0.803
6-keto-PGF1a (pg/ml)	0.31	0.45	0.803
Eicosapentaenoic acid	-0.11	0.451	0.803

Itaconic acid	-0.08	0.452	0.803
Thyroxine	-0.06	0.452	0.803
Betaine	-0.03	0.458	0.803
2-Hydroxycaproic acid	-0.06	0.459	0.803
Leucyl-Aspartate / gamma-Glutamylvaline	0.11	0.46	0.803
gamma-Glutamyltryptophan	-0.04	0.465	0.803
Valerylcarnitine	-0.08	0.466	0.803
LysoPC(0:0/17:0)	-0.08	0.467	0.803
Xanthine	0.08	0.472	0.803
Phenylalanyltryptophan / Tryptophyl-Phenylalanine	-0.09	0.473	0.803
MG(18:2(9Z,12Z)/0:0/0:0)	0.07	0.474	0.803
9 10 13-TriHOME (pg/ml)	-0.13	0.477	0.803
LysoPC(0:0/16:0)	-0.05	0.479	0.803
12(S)-HEPE (pg/ml)	0.15	0.489	0.815
Phenylalanylglutamic acid / gamma-Glutamylphenylalanine	-0.04	0.495	0.819
LysoPE(0:0/18:3(6Z,9Z,12Z))	-0.07	0.496	0.819
LysoPC(P-18:0/0:0)	-0.09	0.5	0.82
L-Proline	0.04	0.502	0.82
L-Kynurenine	-0.05	0.506	0.822
LysoPC(O-16:0/0:0)	-0.08	0.514	0.825
5-Hydroxyindoleacetic acid	-0.06	0.517	0.825
D-Glucoheptose	-0.05	0.519	0.825
L-Leucine	-0.03	0.519	0.825
Isoleucyl-Leucine	0.06	0.52	0.825
3-Hydroxybutyrylcarnitine	0.22	0.527	0.831
Docosapentaenoic acid (22n-3)	-0.12	0.536	0.84
LysoPC(0:0/18:0)	-0.07	0.538	0.84
4-Pyridoxic acid	0.13	0.54	0.84
Lenticin	0.24	0.543	0.84
Lithocholic acid glycine conjugate	-0.08	0.546	0.841
Pyroglutamic acid	-0.03	0.55	0.843
LysoPE(18:0/0:0)	-0.06	0.555	0.843
Stearoylcarnitine	-0.08	0.555	0.843
Indoleacetaldehyde	0.03	0.561	0.846
15(S)-HETrE (pg/ml)	-0.11	0.563	0.846
Glycocholic acid	0.13	0.565	0.846
LysoPC(0:0/20:1)	-0.04	0.57	0.846
L-Tyrosine	-0.03	0.57	0.846
Succinyladenosine	-0.04	0.58	0.851
2-Hydroxyhexadecanoic acid	0.07	0.581	0.851
LysoPE(0:0/18:0)	-0.05	0.583	0.851
Arachidonic acid	-0.08	0.584	0.851
Hexose	-0.05	0.593	0.856
7-Methylguanine	-0.03	0.594	0.856
Phenylalanylisoleucine	-0.04	0.599	0.856
N-gamma-Glutamylglutamine	0.04	0.602	0.856

gamma-Glutamylisoleucine	0.07	0.603	0.856
(+-)9-HETE (pg/ml)	-0.12	0.605	0.856
Guanine	-0.03	0.606	0.856
2-Hydroxy-3-methylpentanoic acid	-0.06	0.608	0.856
(11Z)-Eicoseneoylcarnitine	-0.06	0.62	0.869
9 12 13-TriHOME (pg/ml)	-0.13	0.626	0.873
Salicylic acid	0.19	0.646	0.891
Adenosine	0.09	0.648	0.891
L-Methionine	-0.03	0.651	0.891
Hexanoylcarnitine	-0.07	0.653	0.891
LysoPC(19:0/0:0)	-0.07	0.655	0.891
LysoPC(12:0/0:0)	0.08	0.656	0.891
4-Hydroxybenzoic acid	0.09	0.658	0.891
3-Hydroxyisovaleric acid	-0.03	0.665	0.897
12-HETE (pg/ml)	0.09	0.669	0.898
2-Hydroxymyristic acid	-0.05	0.675	0.903
Chenodeoxycholic acid glycine conjugate	0.07	0.678	0.903
LysoPC(20:2(11Z,14Z)/0:0)	-0.05	0.681	0.904
8(S)-HETE (pg/ml)	-0.07	0.694	0.914
Methylguanosine	0.02	0.695	0.914
LysoPC(20:5(5Z,8Z,11Z,14Z,17Z)/0:0)	-0.05	0.702	0.914
2-Methoxybenzoic acid	0.03	0.704	0.914
LysoPE(16:0/0:0)	-0.03	0.705	0.914
Indole-3-carboxaldehyde	0.04	0.706	0.914
3-Methylcrotonylglycine / Tiglylglycine	-0.05	0.712	0.916
Taurocholic acid	-0.07	0.713	0.916
6-trans-LTB4 (pg/ml)	-0.12	0.719	0.92
13-oxo-ODE (pg/ml)	0.07	0.725	0.921
LysoPC(16:1(9Z)/0:0)	-0.04	0.731	0.921
Dodecanedioic acid	-0.03	0.731	0.921
gamma-Glutamylmethionine	0.03	0.731	0.921
LysoPC(16:0(OH)/0:0)	-0.04	0.734	0.922
LysoPC(15:0/0:0)	0.03	0.741	0.923
alpha-Chaconine	-0.11	0.743	0.923
Hypoxanthine	-0.04	0.75	0.923
Quinic acid	0.06	0.76	0.923
LysoPE(18:2(9Z,12Z)/0:0)	-0.03	0.762	0.923
gamma-Glutamylleucine	0.03	0.763	0.923
LysoPC(0:0/20:5)	-0.04	0.766	0.923
LysoPE(18:3(9Z,12Z,15Z)/0:0)	-0.04	0.766	0.923
Homo-L-arginine	-0.03	0.77	0.923
LysoPC(14:1(9Z)/0:0)	0.04	0.773	0.923
Asymmetric dimethylarginine	-0.02	0.773	0.923
LysoPC(18:0(OH)/0:0)	-0.03	0.775	0.923
Cytosine	0.03	0.775	0.923
LysoPE(0:0/20:5(5Z,8Z,11Z,14Z,17Z))	0.03	0.776	0.923
LysoPC(18:4(6Z,9Z,12Z,15Z)/0:0)	-0.04	0.78	0.923
15-oxo-ETE (pg/ml)	-0.05	0.782	0.923

Chenodeoxycholic acid	0.08	0.784	0.923
4-Hydroxyproline	0.02	0.788	0.924
D-Phenyllactic acid	0.03	0.791	0.925
Taurochenodesoxycholic acid	-0.03	0.806	0.937
LysoPC(18:1(9Z)/0:0)	-0.01	0.807	0.937
Oleamide	0.03	0.812	0.939
LysoPC(18:2(9Z,12Z)/0:0)	0.01	0.824	0.947
Isocitric acid	0.01	0.825	0.947
Sebacic acid	-0.02	0.828	0.947
Creatine	0.03	0.831	0.947
N2,N2-Dimethylguanosine	0.02	0.837	0.947
Phenylalanylphenylalanine	-0.02	0.839	0.947
1-Aminocyclopropanecarboxylic acid	0.01	0.839	0.947
3-Hydroxytetradecanoic acid	0.01	0.843	0.947
LysoPC(0:0/18:0(OH))	-0.03	0.845	0.947
Pantothenic acid	-0.02	0.849	0.948
Azelaic acid	-0.02	0.857	0.952
LysoPC(18:3(9Z,12Z,15Z)/0:0)	-0.02	0.862	0.952
L-Lysine	-0.01	0.864	0.952
LysoPC(0:0/18:3)	-0.02	0.866	0.952
PGD2 (pg/ml)	0.04	0.867	0.952
15-HETE (pg/ml)	0.02	0.878	0.958
Cholic acid	-0.04	0.879	0.958
L-Tryptophan	0.01	0.881	0.958
LysoPE(22:5(4Z,7Z,10Z,13Z,16Z)/0:0)	-0.01	0.884	0.958
8(9)-EpETrE (pg/ml)	0.02	0.892	0.963
17-HDOHE (pg/ml)	0.02	0.895	0.963
LysoPE(0:0/16:0)	0.01	0.9	0.965
Sphingosine 1-phosphate	0.01	0.905	0.968
5-oxo-ETE (pg/ml)	-0.02	0.925	0.979
Citraconic acid	-0.02	0.927	0.979
LysoPE(0:0/18:2(9Z,12Z))	0.01	0.929	0.979
Trimethylamine N-oxide	-0.01	0.931	0.979
5 6-DiHETrE (pg/ml)	-0.01	0.933	0.979
LysoPC(0:0/15:0)	0.01	0.933	0.979
LysoPE(20:5(5Z,8Z,11Z,14Z,17Z)/0:0)	-0.01	0.951	0.991
Citrulline	0	0.952	0.991
LysoPC(0:0/16:1)	-0.01	0.957	0.993
LysoPC(0:0/14:0)	0.01	0.967	0.993
Epinephrine	0	0.967	0.993
LysoPC(20:0/0:0)	-0.01	0.968	0.993
Hexadecanedioic acid	0	0.969	0.993
LysoPE(0:0/18:1(9Z))	0	0.973	0.994
LysoPE(18:1(9Z)/0:0)	0	0.979	0.997
2,3,4,5,6,7-Hexahydroxyheptanoic acid	0	0.984	0.999
TxB2 (pg/ml)	0.01	0.991	1
(+-)11-HETE (pg/ml)	0	0.997	1
LysoPC(0:0/18:2(9Z,12Z))	0	0.998	1

LysoPC(14:0/0:0)	0	0.999	1
5-HETE (pg/ml)	0	1	1

Supplementary Table S3. The differences in relative metabolomic concentrations between patients with IBS-like symptoms or not

variable	mean difference	p_value	adj_p_value
4-Coumaryl alcohol	-0.73	0.008	0.999
Bis(2-ethylhexyl)phthalate	-0.13	0.018	0.999
4-Pyridoxic acid	0.47	0.026	0.999
Pantothenic acid	0.25	0.029	0.999
Tiglylcarnitine	-0.22	0.047	0.999
LysoPC(0:0/15:0)	-0.23	0.05	0.999
3-Hydroxyisovalerylcarnitine	-0.2	0.05	0.999
3-Hydroxyisovaleric acid	-0.14	0.053	0.999
LysoPC(15:0/0:0)	-0.18	0.055	0.999
MG(18:2(9Z,12Z)/0:0/0:0)	-0.16	0.07	0.999
LysoPE(O-18:0/0:0)	-0.22	0.071	0.999
LysoPC(0:0/14:0)	-0.22	0.079	0.999
LysoPC(17:0/0:0)	-0.22	0.08	0.999
LysoPE(P-18:0/0:0)	-0.26	0.083	0.999
LysoPC(14:0/0:0)	-0.21	0.083	0.999
4-Hydroxyproline	0.11	0.093	0.999
Isocitric acid	-0.07	0.104	0.999
LysoPC(14:1(9Z)/0:0)	-0.21	0.105	0.999
Phenylalanylphenylalanine	0.16	0.108	0.999
L-Proline	-0.1	0.108	0.999
Serotonin	1.22	0.109	0.999
Deoxycholic acid glycine conjugate	-0.37	0.112	0.999
LysoPC(0:0/17:0)	-0.17	0.113	0.999
LysoPC(0:0/18:0)	-0.18	0.115	0.999
LysoPE(O-16:0/0:0)	-0.2	0.116	0.999
LysoPC(0:0/19:0)	-0.16	0.117	0.999
1,11-Undecanedicarboxylic acid	0.13	0.121	0.999
Adenosine	-0.28	0.122	0.999
Octadecanedioic acid	-0.14	0.126	0.999
Cortisol / hydrocortisone	-0.26	0.132	0.999
Glycocholic acid	-0.33	0.134	0.999
Bilirubin	-0.19	0.134	0.999
Citric acid	-0.05	0.138	0.999
3a,7b,12a-Trihydroxyoxocholanyl-Glycine	-0.21	0.148	0.999
Cinnamoylglycine	-0.35	0.159	0.999
LysoPE(P-16:0/0:0)	-0.18	0.16	0.999
1-Aminocyclopropanecarboxylic acid	0.08	0.163	0.999
LysoPC(18:1(9Z)/0:0)	-0.06	0.163	0.999
Guanine	0.09	0.166	0.999
LysoPC(O-16:0/0:0)	-0.17	0.167	0.999
LysoPC(0:0/18:1(9Z))	-0.08	0.169	0.999
Chenodeoxycholic acid glycine conjugate	-0.2	0.175	0.999
Indolelactic acid	-0.11	0.176	0.999
LysoPC(0:0/16:0)	-0.1	0.184	0.999

LysoPC(19:0/0:0)	-0.2	0.186	0.999
Oxoglutaric acid	-0.08	0.187	0.999
3-Hydroxybutyrylcarnitine	0.45	0.187	0.999
gamma-Glutamylmethionine	0.13	0.189	0.999
Lenticin	0.51	0.191	0.999
Creatine	0.16	0.195	0.999
LysoPC(20:0/0:0)	-0.16	0.197	0.999
LysoPC(16:0/0:0)	-0.03	0.197	0.999
Undecanedioic acid	0.09	0.198	0.999
LysoPE(0:0/18:0)	-0.11	0.201	0.999
Valerylcarnitine	-0.14	0.205	0.999
2-Hydroxy-3-methylbutyric acid	-0.16	0.21	0.999
L-Methionine	0.08	0.211	0.999
Citrulline	-0.09	0.213	0.999
5 6-DiHETrE (pg/ml)	0.15	0.218	0.999
LysoPC(12:0/0:0)	-0.22	0.219	0.999
7-Methylguanine	0.07	0.221	0.999
Oleamide	0.15	0.225	0.999
Acetoacetic acid	-0.22	0.23	0.999
Dodecanedioic acid	0.11	0.231	0.999
Hexose	-0.1	0.236	0.999
gamma-Glutamyltyrosine	-0.09	0.236	0.999
N-(3-acetamidopropyl)pyrrolidin-2-one	-0.11	0.237	0.999
Isovalerylcarnitine	-0.11	0.239	0.999
Hydroxyoctanoic acid	0.15	0.242	0.999
3-Methylcrotonylglycine / Tiglylglycine	-0.16	0.251	0.999
LysoPC(18:0/0:0)	-0.06	0.26	0.999
Succinic acid	0.06	0.266	0.999
9 12 13-TriHOME (pg/ml)	-0.31	0.269	0.999
Niacinamide	0.12	0.273	0.999
Cytosine	-0.14	0.28	0.999
Isoleucyl-Leucine	0.11	0.282	0.999
Phenylalanyltryptophan / Tryptophyl-Phenylalanine	0.13	0.294	0.999
gamma-Glutamylalanine	0.11	0.3	0.999
LysoPC(P-18:0/0:0)	-0.13	0.315	0.999
(±)-2-Hydroxy-4-(methylthio)butanoic acid	-0.09	0.321	0.999
LysoPC(20:2(11Z,14Z)/0:0)	-0.12	0.322	0.999
12(S)-HEPE (pg/ml)	-0.21	0.328	0.999
Inosine	-0.12	0.337	0.999
2-Octenoylcarnitine	0.15	0.341	0.999
2-Hydroxy-3-methylpentanoic acid	-0.11	0.343	0.999
5(s)6(R)-LXA4 (pg/ml)	-0.19	0.344	0.999
Indole-3-carboxaldehyde	0.11	0.345	0.999
PGE2 (pg/ml)	0.38	0.346	0.999
LysoPC(20:1(11Z)/0:0)	-0.11	0.348	0.999
N-Acetylserine	0.08	0.349	0.999

3-(3-Hydroxyphenyl)-3-hydroxypropanoic acid / 4-Hydroxyphenyllactic acid	-0.07	0.352	0.999
LysoPE(22:4(7Z,10Z,13Z,16Z)/0:0)	-0.07	0.361	0.999
LysoPC(0:0/20:2)	-0.07	0.362	0.999
Chenodeoxycholic acid	0.24	0.363	0.999
LysoPE(18:0/0:0)	-0.09	0.364	0.999
12 13-DiHOME (pg/ml)	-0.17	0.376	0.999
Tetradecanedioic acid	-0.15	0.376	0.999
D-Glucoheptose	0.06	0.376	0.999
1-Methyladenosine	0.06	0.396	0.999
L-Lysine	0.05	0.397	0.999
AICAR	0.07	0.402	0.999
LysoPC(0:0/20:1)	-0.06	0.408	0.999
LysoPE(20:5(5Z,8Z,11Z,14Z,17Z)/0:0)	-0.1	0.41	0.999
L-Kynurenine	0.07	0.415	0.999
12-HETE (pg/ml)	-0.17	0.415	0.999
Ursodeoxycholic acid	0.16	0.417	0.999
Hippuric acid	0.16	0.423	0.999
Cortisone	-0.11	0.43	0.999
LTB4 (pg/ml)	-0.31	0.435	0.999
Butyrylcarnitine	0.09	0.436	0.999
6-keto-PGF1a (pg/ml)	0.32	0.437	0.999
3-Hydroxycapric acid	0.1	0.438	0.999
L-Tyrosine	-0.05	0.44	0.999
LysoPC(16:1(9Z)/0:0)	-0.08	0.449	0.999
3-Hydroxyoctanoic acid	0.09	0.454	0.999
L-Sorbose	0.13	0.455	0.999
LysoPC(18:2(9Z,12Z)/0:0)	-0.03	0.455	0.999
Asymmetric dimethylarginine	-0.06	0.455	0.999
Glutarylcarnitine	-0.07	0.464	0.999
S-Adenosylhomocysteine	-0.09	0.466	0.999
L-Isoleucine	0.04	0.469	0.999
LysoPC(20:3(8Z,11Z,14Z)/0:0)	-0.06	0.47	0.999
(2E)-3-(4-hydroxy-3-methoxyphenyl)prop-2-enal	-0.13	0.471	0.999
3-Methoxytyrosine	0.05	0.472	0.999
Arachidonic acid	0.1	0.48	0.999
(5Z,8Z,11Z,14Z,17Z)-Icosa-5,8,11,14,17-pentaenoylcarnitine	-0.09	0.483	0.999
LysoPE(0:0/20:5(5Z,8Z,11Z,14Z,17Z))	-0.07	0.483	0.999
Prolylhydroxyproline / Pyroglutamylvaline	0.08	0.484	0.999
Acetaminophen	0.48	0.486	0.999
LysoPC(0:0/22:6)	-0.08	0.491	0.999
Phenylacetylglutamine	-0.14	0.491	0.999
(S)-3-Hydroxyisobutyric acid	-0.07	0.494	0.999
5-Hydroxyindoleacetic acid	0.06	0.499	0.999
Salicylic acid	-0.27	0.504	0.999
2-Methoxybenzoic acid	-0.05	0.505	0.999

LysoPE(0:0/16:0)	-0.06	0.507	0.999
Xanthine	0.07	0.509	0.999
Docosapentaenoylcarnitine	-0.08	0.51	0.999
LysoPE(0:0/18:1(9Z))	-0.07	0.515	0.999
D-Leucic acid	0.05	0.515	0.999
Cholic acid	-0.16	0.516	0.999
Methylguanosine	0.04	0.516	0.999
Glutaric acid	-0.05	0.517	0.999
Threonic acid	-0.1	0.519	0.999
alpha-Linolenic acid	-0.12	0.528	0.999
Malic acid	-0.04	0.534	0.999
(R)-3-Hydroxy-hexadecanoic acid	0.07	0.534	0.999
gamma-Glutamylisoleucine	0.08	0.536	0.999
Pyroglutamic acid	0.03	0.541	0.999
3-Hydroxydodecanoic acid	0.08	0.543	0.999
LysoPC(0:0/20:3)	-0.06	0.548	0.999
12-OxoETE (pg/ml)	-0.1	0.55	0.999
N-Formyl-L-methionine	0.03	0.55	0.999
LysoPC(0:0/20:4)	-0.05	0.553	0.999
LysoPC(0:0/22:5)	-0.04	0.562	0.999
Pipecolic acid	0.06	0.564	0.999
Isobutyryl-L-carnitine	-0.08	0.566	0.999
Taurocholic acid	-0.1	0.572	0.999
LysoPC(0:0/18:2(9Z,12Z))	-0.05	0.576	0.999
4-Hydroxybenzoic acid	-0.11	0.576	0.999
LysoPC(22:6(4Z,7Z,10Z,13Z,16Z,19Z)/0:0)	-0.04	0.576	0.999
LysoPC(0:0/18:0(OH))	-0.07	0.576	0.999
Stearic acid	0.1	0.577	0.999
5(6)-EpETrE (pg/ml)	0.07	0.58	0.999
LysoPC(18:3(9Z,12Z,15Z)/0:0)	-0.06	0.58	0.999
LysoPC(22:5(4Z,7Z,10Z,13Z,16Z)/0:0)	-0.06	0.583	0.999
8(S)-HETE (pg/ml)	0.1	0.586	0.999
Oleic acid / Elaidic acid	-0.1	0.586	0.999
Palmitic acid	0.08	0.588	0.999
Guanosine	-0.07	0.59	0.999
Thyroxine	-0.04	0.591	0.999
3-Hydroxybutyric acid	-0.12	0.597	0.999
9 10-DiHOME (pg/ml)	-0.11	0.599	0.999
Tetradecanoylcarnitine	-0.08	0.6	0.999
Cervonylcarnitine	-0.06	0.602	0.999
N-Acetyl-L-methionine	0.04	0.603	0.999
LysoPE(16:0/0:0)	-0.05	0.606	0.999
LysoPC(0:0/20:5)	-0.07	0.614	0.999
LysoPC(20:5(5Z,8Z,11Z,14Z,17Z)/0:0)	-0.06	0.614	0.999
D-Phenyllactic acid	0.06	0.619	0.999
LysoPC(0:0/16:1)	-0.05	0.628	0.999
2,4-Dihydroxybenzoic acid / 2-Pyrocatechuic acid	-0.08	0.628	0.999

Itaconic acid	-0.05	0.629	0.999
2',4'-Dihydroxyacetophenone (Resorcinol monoacetate)	0.16	0.631	0.999
N-gamma-Glutamylglutamine	0.03	0.638	0.999
Epinephrine	-0.05	0.641	0.999
LysoPC(22:4(7Z,10Z,13Z,16Z)/0:0)	0.04	0.644	0.999
Hexanoylcarnitine	0.07	0.644	0.999
LysoPE(18:2(9Z,12Z)/0:0)	-0.05	0.644	0.999
alpha-Chaconine	0.15	0.645	0.999
8-iso-PGE2 (pg/ml)	-0.17	0.647	0.999
Uridine/Pseudouridine	0.04	0.647	0.999
LysoPE(18:1(9Z)/0:0)	-0.05	0.651	0.999
LysoPC(18:4(6Z,9Z,12Z,15Z)/0:0)	-0.07	0.652	0.999
9(S)-HODE (pg/ml)	-0.09	0.655	0.999
Indoleacetaldehyde	0.02	0.656	0.999
Indoleacetic acid	-0.05	0.658	0.999
Homo-L-arginine	0.04	0.66	0.999
LysoPE(0:0/18:2(9Z,12Z))	-0.05	0.665	0.999
Glycoursodeoxycholic acid	0.09	0.668	0.999
5-oxo-ETE (pg/ml)	0.09	0.672	0.999
20-HETE (pg/ml)	-0.05	0.673	0.999
LysoPC(0:0/22:4)	-0.03	0.676	0.999
N-Acetyl-L-alanine	0.02	0.677	0.999
3-Carboxy-4-methyl-5-propyl-2-furanpropionic acid	-0.08	0.678	0.999
14 15-DiHETrE (pg/ml)	0.04	0.679	0.999
gamma-Aminobutyric acid	-0.03	0.683	0.999
LysoPC(0:0/18:3)	-0.05	0.684	0.999
cis-4-Decenoylcarnitine	-0.06	0.686	0.999
Glyceric acid	-0.03	0.692	0.999
Betaine	-0.01	0.693	0.999
Propionylcarnitine	-0.03	0.693	0.999
3-Hydroxytetradecanoic acid	-0.03	0.694	0.999
N2,N2-Dimethylguanosine	-0.04	0.7	0.999
2,3,4,5,6,7-Hexahydroxyheptanoic acid	-0.01	0.707	0.999
gamma-Glutamyltryptophan	-0.02	0.708	0.999
12(13)-EpOME (pg/ml)	-0.09	0.709	0.999
LysoPE(22:5(4Z,7Z,10Z,13Z,16Z)/0:0)	-0.03	0.71	0.999
Trigonelline	0.07	0.711	0.999
Leucyl-Aspartate / gamma-Glutamylvaline	0.05	0.718	0.999
L-Phenylalanine	0.01	0.718	0.999
LysoPE(22:6(4Z,7Z,10Z,13Z,16Z,19Z)/0:0)	0.03	0.728	0.999
Uracil	0.03	0.729	0.999
Pimelic acid / 3-Methyladipic acid	-0.02	0.733	0.999
Docosahexaenoic acid	-0.05	0.737	0.999
LysoPE(0:0/18:3(6Z,9Z,12Z))	0.04	0.739	0.999
LysoPE(0:0/20:4(5Z,8Z,11Z,14Z))	-0.02	0.741	0.999
11(12)-EpETrE (pg/ml)	0.04	0.756	0.999

3-Methylglutarylcarnitine	0.04	0.757	0.999
2-Hydroxymyristic acid	0.03	0.76	0.999
Adipic acid	-0.02	0.763	0.999
Caffeine	-0.08	0.774	0.999
2-Hydroxystearic acid	0.06	0.774	0.999
5-HETE (pg/ml)	0.08	0.78	0.999
15-oxo-ETE (pg/ml)	-0.05	0.781	0.999
13-oxo-ODE (pg/ml)	0.06	0.783	0.999
2-Hydroxyadipic acid / 3-Hydroxyadipic acid / 3-Hydroxymethylglutaric acid	-0.01	0.784	0.999
Succinyladenosine	0.02	0.787	0.999
Linoleylcarnitine	0.03	0.788	0.999
Azelaic acid	0.03	0.789	0.999
Phenylalanylglutamic acid / gamma-Glutamylphenylalanine	-0.02	0.791	0.999
Capryloylglycine	0.04	0.793	0.999
11 12-DiHETrE (pg/ml)	-0.03	0.794	0.999
alpha-Dimorphecolic acid	0.03	0.794	0.999
LysoPC(18:0(OH)/0:0)	-0.03	0.799	0.999
Sebacic acid	-0.02	0.799	0.999
4-Hydroxyhippuric acid	0.04	0.803	0.999
Ketoleucine	0.02	0.806	0.999
12-Hydroxystearic acid	0.03	0.807	0.999
9 10 13-TriHOME (pg/ml)	0.04	0.808	0.999
17-HDOHE (pg/ml)	-0.05	0.808	0.999
Linoleic acid	-0.03	0.811	0.999
Taurochenodesoxycholic acid	-0.03	0.814	0.999
L-Tryptophan	0.01	0.816	0.999
Eicosapentaenoic acid	-0.03	0.816	0.999
Theobromine	-0.05	0.817	0.999
15-HETE (pg/ml)	-0.03	0.821	0.999
(7Z,10Z,13Z,16Z)-Docosatetraenoylcarnitine	0.02	0.825	0.999
Lithocholic acid glycine conjugate	-0.03	0.826	0.999
6-trans-LTB4 (pg/ml)	0.07	0.826	0.999
Creatinine	0.01	0.829	0.999
Hypoxanthine	-0.03	0.83	0.999
Paraxanthine	0.06	0.831	0.999
Stearoylcarnitine	-0.03	0.832	0.999
Icosa-8,11,14-trienoylcarnitine	-0.02	0.837	0.999
4-Acetamidobutanoic acid	-0.01	0.838	0.999
9(10)-EpOME (pg/ml)	0.04	0.84	0.999
2-Hydroxydecanoate	-0.02	0.844	0.999
gamma-Glutamylleucine	0.02	0.848	0.999
TxB2 (pg/ml)	-0.09	0.851	0.999
Indole-3-propionic acid	-0.05	0.854	0.999
2-Hydroxycaproic acid	0.01	0.855	0.999
15(S)-HETrE (pg/ml)	0.03	0.855	0.999
Dodecanoylcarnitine	-0.03	0.857	0.999

L,L-Cyclo(leucylprolyl)	0.04	0.86	0.999
2-Hydroxyhexadecanoic acid	-0.02	0.866	0.999
LysoPE(18:3(9Z,12Z,15Z)/0:0)	-0.02	0.868	0.999
Myristoleoylcarnitine	-0.03	0.874	0.999
8(9)-EpETrE (pg/ml)	-0.02	0.877	0.999
Suberic acid	-0.01	0.881	0.999
L-Octanoylcarnitine	0.02	0.886	0.999
trans-2-Dodecenoylcarnitine	0.03	0.889	0.999
Sphingosine 1-phosphate	0.01	0.894	0.999
Phenylalanylisoleucine	0.01	0.895	0.999
Isoleucyl-Isoleucine	0.01	0.896	0.999
Oleoylcarnitine	0.02	0.898	0.999
(+-)11-HETE (pg/ml)	0.02	0.901	0.999
3beta,7alpha-Dihydroxy-5-cholestenoate	-0.01	0.905	0.999
2-Hydroxylauroylcarnitine	-0.02	0.913	0.999
MG(18:1(9Z)/0:0/0:0)	-0.01	0.916	0.999
5'-Methylthioadenosine	-0.01	0.919	0.999
(+-)9-HETE (pg/ml)	0.02	0.932	0.999
Gamma-linolenyl arnitine	-0.01	0.934	0.999
8 9-DiHETrE (pg/ml)	-0.01	0.935	0.999
Arachidonoylcarnitine	0.01	0.936	0.999
Docosapentaenoic acid (22n-3)	0.01	0.952	0.999
Deoxycholic acid	0.01	0.953	0.999
L-Leucine	0	0.954	0.999
3, 5-Tetradecadiencarnitine	-0.01	0.955	0.999
LysoPE(20:4(5Z,8Z,11Z,14Z)/0:0)	0	0.955	0.999
LysoPE(0:0/22:6(4Z,7Z,10Z,13Z,16Z,19Z))	0	0.957	0.999
LysoPC(20:4(5Z,8Z,11Z,14Z)/0:0)	0	0.957	0.999
2-Hydroxymyristoylcarnitine	0.01	0.961	0.999
13-HODE (pg/ml)	0.01	0.965	0.999
alpha-Ketoisovaleric acid	0	0.965	0.999
Hexadecanedioic acid	0	0.966	0.999
Methylmalonylcarnitine	0	0.969	0.999
Uric acid	0	0.969	0.999
Theophylline	-0.01	0.97	0.999
Decanoylcarnitine	0.01	0.973	0.999
(11Z)-Eicoseneoylcarnitine	0	0.973	0.999
9,12-Hexadecadienoylcarnitine	-0.01	0.974	0.999
Indoxyl sulfate	0	0.974	0.999
Trimethylamine N-oxide	0	0.976	0.999
Citraconic acid	0	0.979	0.999
L-Acetylcarnitine	0	0.986	0.999
Quinic acid	0	0.986	0.999
PGD2 (pg/ml)	0	0.987	0.999
Kynurenic acid	0	0.991	0.999
LysoPC(16:0(OH)/0:0)	0	0.992	0.999
Levonorgestrel	0	0.994	0.999
Phenylalanylproline	0	0.995	0.999

3-Hydroxyoctadecenoylcarnitine	0	0.996	0.999
L-Palmitoylcarnitine	0	0.996	0.999
9-Hexadecenoylcarnitine	0	0.999	0.999

Supplementary Table S4. The differences in relative metabolomic concentrations between users of corticosteroids or not

variable	mean difference	p_value	adj_p_value
Cortisone	-0.55	0.004	0.357
Cortisol / hydrocortisone	-0.62	0.007	0.357
Dodecanoylcarnitine	-0.46	0.007	0.357
Tiglylcarnitine	-0.29	0.008	0.357
Icosa-8,11,14-trienoylcarnitine	-0.33	0.008	0.357
11 12-DiHETrE (pg/ml)	-0.28	0.009	0.357
Decanoylcarnitine	-0.48	0.009	0.357
1,11-Undecanedicarboxylic acid	0.25	0.009	0.357
Uridine/Pseudouridine	-0.21	0.011	0.357
Indolelactic acid	-0.19	0.012	0.357
gamma-Glutamylalanine	0.28	0.012	0.357
2-Hydroxylauroylcarnitine	-0.36	0.013	0.357
Tetradecanoylcarnitine	-0.37	0.016	0.395
3beta,7alpha-Dihydroxy-5-cholestenoate	-0.3	0.019	0.395
Paraxanthine	0.51	0.02	0.395
3-Hydroxyisovaleric acid	-0.18	0.021	0.395
Theophylline	0.43	0.022	0.395
Arachidonoylcarnitine	-0.29	0.023	0.395
Lithocholic acid glycine conjugate	-0.33	0.023	0.395
trans-2-Dodecenoylcarnitine	-0.42	0.031	0.429
L-Octanoylcarnitine	-0.39	0.033	0.429
L-Sorbose	0.4	0.034	0.429
3-Hydroxybutyric acid	-0.47	0.034	0.429
Indoleacetic acid	0.26	0.035	0.429
Hippuric acid	0.45	0.036	0.429
Sphingosine 1-phosphate	-0.15	0.036	0.429
14 15-DiHETrE (pg/ml)	-0.18	0.038	0.429
Myristoleoylcarnitine	-0.39	0.039	0.429
Glycocholic acid	0.54	0.041	0.429
Docosapentaenoylcarnitine	-0.24	0.041	0.429
gamma-Glutamylmethionine	0.25	0.044	0.429
Adipic acid	-0.12	0.045	0.429
Chenodeoxycholic acid glycine conjugate	0.34	0.045	0.429
(7Z,10Z,13Z,16Z)-Docosatetraenoylcarnitine	-0.26	0.046	0.429
3-Hydroxyisovalerylcarnitine	-0.2	0.047	0.429
3, 5-Tetradecadiencarnitine	-0.37	0.049	0.429
3-Hydroxydodecanoic acid	-0.24	0.049	0.429
cis-4-Decenoylcarnitine	-0.34	0.05	0.429
(2E)-3-(4-hydroxy-3-methoxyphenyl)prop-2-enal	0.34	0.054	0.442
L-Acetylcarnitine	-0.18	0.055	0.442
2-Hydroxymyristic acid	-0.22	0.056	0.442
Cervonylcarnitine	-0.24	0.059	0.45
6-trans-LTB4 (pg/ml)	-0.6	0.06	0.45

3-Hydroxytetradecanoic acid	-0.15	0.069	0.506
Caffeine	0.41	0.071	0.506
Acetaminophen	1.44	0.074	0.506
9,12-Hexadecadienoylcarnitine	-0.32	0.075	0.506
alpha-Dimorphecolic acid	-0.24	0.075	0.506
Leucyl-Aspartate / gamma-Glutamylvaline	0.32	0.078	0.516
2-Hydroxy-3-methylbutyric acid	-0.23	0.08	0.517
Succinic acid	-0.09	0.081	0.517
9-Hexadecenoylcarnitine	-0.28	0.083	0.517
Uracil	-0.18	0.084	0.517
gamma-Glutamylleucine	0.24	0.088	0.521
Oleoylcarnitine	-0.25	0.088	0.521
Linoleylcarnitine	-0.24	0.09	0.521
Undecanedioic acid	0.13	0.092	0.523
LysoPE(22:4(7Z,10Z,13Z,16Z)/0:0)	-0.12	0.093	0.523
gamma-Glutamylisoleucine	0.27	0.103	0.569
L-Palmitoylcarnitine	-0.23	0.109	0.59
Threonic acid	-0.26	0.111	0.594
Deoxycholic acid glycine conjugate	0.43	0.116	0.61
Glutarylcarnitine	-0.17	0.122	0.625
Gamma-linolenyl arnitine	-0.22	0.124	0.625
Cinnamoylglycine	0.41	0.125	0.625
Acetoacetic acid	-0.33	0.127	0.625
Guanine	0.12	0.131	0.636
Oleic acid / Elaidic acid	-0.29	0.133	0.636
8 9-DiHETrE (pg/ml)	-0.15	0.136	0.643
Pipecolic acid	0.15	0.141	0.646
L-Methionine	0.12	0.141	0.646
Homo-L-arginine	0.15	0.147	0.665
LysoPC(0:0/18:1(9Z))	-0.1	0.152	0.671
2-Hydroxyhexadecanoic acid	-0.22	0.155	0.671
9 10-DiHOME (pg/ml)	0.36	0.158	0.671
N-gamma-Glutamylglutamine	0.1	0.158	0.671
3-Hydroxyoctadecenoylcarnitine	-0.26	0.16	0.671
9 12 13-TriHOME (pg/ml)	0.43	0.161	0.671
Trimethylamine N-oxide	0.23	0.174	0.716
15(S)-HETrE (pg/ml)	-0.23	0.184	0.729
Indoxyl sulfate	0.21	0.186	0.729
(5Z,8Z,11Z,14Z,17Z)-Icosa-5,8,11,14,17-pentaenoylcarnitine	-0.17	0.188	0.729
Hexose	-0.11	0.189	0.729
(S)-3-Hydroxyisobutyric acid	-0.15	0.19	0.729
Linoleic acid	-0.23	0.192	0.729
Suberic acid	-0.07	0.193	0.729
Butyrylcarnitine	0.17	0.199	0.736
LysoPC(0:0/22:4)	-0.12	0.199	0.736
Theobromine	0.27	0.203	0.741
4-Pyridoxic acid	0.38	0.207	0.741

3a,7b,12a-Trihydroxyoxocholanyl-Glycine	0.2	0.207	0.741
(11Z)-Eicoseneoylcarnitine	-0.17	0.218	0.761
gamma-Glutamyltyrosine	0.1	0.218	0.761
LysoPE(0:0/18:1(9Z))	-0.14	0.221	0.763
L-Lysine	0.08	0.228	0.771
Capryloylglycine	-0.17	0.229	0.771
3-Hydroxycapric acid	-0.14	0.232	0.771
Docosahexaenoic acid	-0.19	0.232	0.771
LysoPE(18:1(9Z)/0:0)	-0.14	0.237	0.778
Bilirubin	-0.16	0.24	0.778
3-Methylglutarylcarnitine	-0.16	0.244	0.784
Ursodeoxycholic acid	0.23	0.25	0.797
Hexanoylcarnitine	-0.19	0.258	0.813
LysoPC(22:4(7Z,10Z,13Z,16Z)/0:0)	-0.1	0.263	0.82
N2,N2-Dimethylguanosine	-0.1	0.265	0.822
12-OxoETE (pg/ml)	-0.18	0.268	0.823
2-Hydroxymyristoylcarnitine	-0.17	0.273	0.829
Methylguanosine	-0.06	0.279	0.829
Phenylacetylglutamine	0.21	0.281	0.829
LTB4 (pg/ml)	-0.47	0.282	0.829
PGE2 (pg/ml)	0.47	0.283	0.829
L,L-Cyclo(leucylprolyl)	0.21	0.286	0.829
Uric acid	0.05	0.293	0.829
Stearoylcarnitine	-0.15	0.295	0.829
alpha-Linolenic acid	-0.24	0.295	0.829
L-Tyrosine	0.08	0.298	0.829
LysoPC(20:1(11Z)/0:0)	-0.14	0.299	0.829
Betaine	0.04	0.306	0.839
12 13-DiHOME (pg/ml)	0.23	0.309	0.839
LysoPC(18:1(9Z)/0:0)	-0.05	0.31	0.839
2-Octenoylcarnitine	0.18	0.317	0.845
Docosapentaenoic acid (22n-3)	-0.23	0.32	0.845
Taurocholic acid	-0.17	0.32	0.845
MG(18:1(9Z)/0:0/0:0)	0.14	0.325	0.845
4-Hydroxyhippuric acid	0.14	0.33	0.845
Levonorgestrel	-0.15	0.331	0.845
15-HETE (pg/ml)	-0.13	0.333	0.845
LysoPC(12:0/0:0)	-0.18	0.333	0.845
Citraconic acid	-0.17	0.336	0.846
S-Adenosylhomocysteine	0.14	0.346	0.855
Quinic acid	0.17	0.348	0.855
3-Carboxy-4-methyl-5-propyl-2-furanpropionic acid	0.17	0.352	0.855
Isoleucyl-Isoleucine	-0.1	0.353	0.855
15-oxo-ETE (pg/ml)	-0.16	0.356	0.855
Isovalerylcarnitine	-0.09	0.358	0.855
N-Acetyl-L-alanine	-0.04	0.36	0.855
Chenodeoxycholic acid	0.28	0.36	0.855

Indole-3-propionic acid	0.27	0.364	0.857
Asymmetric dimethylarginine	-0.08	0.369	0.864
Malic acid	-0.05	0.374	0.867
D-Leucic acid	-0.06	0.38	0.871
Inosine	0.12	0.38	0.871
20-HETE (pg/ml)	-0.11	0.385	0.872
4-Acetamidobutanoic acid	-0.04	0.387	0.872
Deoxycholic acid	0.24	0.392	0.878
8(S)-HETE (pg/ml)	-0.13	0.403	0.892
1-Aminocyclopropanecarboxylic acid	0.05	0.404	0.892
Phenylalanylproline	0.09	0.411	0.892
Valerylcarnitine	0.1	0.418	0.892
LysoPC(0:0/20:1)	-0.07	0.424	0.892
LysoPE(18:3(9Z,12Z,15Z)/0:0)	-0.1	0.426	0.892
LysoPC(0:0/20:4)	-0.07	0.427	0.892
2,4-Dihydroxybenzoic acid / 2-Pyrocatechuic acid	-0.14	0.435	0.892
Taurochenodesoxycholic acid	-0.1	0.443	0.892
Pimelic acid / 3-Methyladipic acid	-0.04	0.444	0.892
3-Methoxytyrosine	-0.06	0.444	0.892
Xanthine	0.12	0.446	0.892
L-Leucine	0.03	0.449	0.892
LysoPE(O-16:0/0:0)	-0.1	0.453	0.892
Itaconic acid	-0.08	0.455	0.892
LysoPC(16:0(OH)/0:0)	0.08	0.455	0.892
Guanosine	0.1	0.455	0.892
Phenylalanylglutamic acid / gamma-Glutamylphenylalanine	0.05	0.456	0.892
9 10 13-TriHOME (pg/ml)	0.14	0.456	0.892
Arachidonic acid	-0.12	0.457	0.892
Hydroxyoctanoic acid	-0.1	0.458	0.892
N-Acetylserine	0.07	0.458	0.892
Dodecanedioic acid	0.07	0.464	0.898
LysoPC(0:0/16:0)	-0.06	0.473	0.907
Indoleacetaldehyde	0.04	0.475	0.907
2-Methoxybenzoic acid	-0.06	0.484	0.91
LysoPC(18:3(9Z,12Z,15Z)/0:0)	-0.09	0.485	0.91
LysoPE(O-18:0/0:0)	-0.1	0.486	0.91
LysoPE(18:2(9Z,12Z)/0:0)	-0.08	0.487	0.91
AICAR	0.06	0.492	0.91
LysoPC(0:0/22:5)	-0.05	0.493	0.91
Indole-3-carboxaldehyde	-0.09	0.5	0.911
Phenylalanyltryptophan / Tryptophyl-Phenylalanine	-0.1	0.502	0.911
4-Coumaryl alcohol	0.21	0.502	0.911
LysoPC(14:1(9Z)/0:0)	-0.1	0.508	0.913
LysoPC(0:0/22:6)	-0.09	0.51	0.913
LysoPC(22:5(4Z,7Z,10Z,13Z,16Z)/0:0)	-0.08	0.514	0.913

2-Hydroxystearic acid	-0.17	0.517	0.913
Pyroglutamic acid	0.04	0.52	0.913
LysoPC(20:2(11Z,14Z)/0:0)	-0.09	0.52	0.913
3-Methylcrotonylglycine / Tiglylglycine	0.1	0.526	0.916
LysoPC(22:6(4Z,7Z,10Z,13Z,16Z,19Z)/0:0)	-0.05	0.535	0.916
LysoPE(0:0/18:3(6Z,9Z,12Z))	-0.07	0.535	0.916
LysoPC(0:0/20:2)	-0.05	0.537	0.916
L-Tryptophan	0.03	0.537	0.916
N-Formyl-L-methionine	-0.03	0.539	0.916
Glycoursodeoxycholic acid	0.13	0.541	0.916
Phenylalanylisoleucine	-0.05	0.554	0.928
LysoPC(P-18:0/0:0)	-0.09	0.559	0.928
Thyroxine	-0.05	0.563	0.928
MG(18:2(9Z,12Z)/0:0/0:0)	0.05	0.571	0.928
Isobutyryl-L-carnitine	0.08	0.573	0.928
Hypoxanthine	-0.07	0.576	0.928
5 6-DiHETrE (pg/ml)	0.08	0.578	0.928
Phenylalanylphenylalanine	-0.05	0.58	0.928
Kynurenic acid	-0.06	0.58	0.928
Niacinamide	0.09	0.581	0.928
Lenticin	0.22	0.583	0.928
12(S)-HEPE (pg/ml)	0.12	0.584	0.928
N-(3-acetamidopropyl)pyrrolidin-2-one	-0.06	0.585	0.928
LysoPC(18:0(OH)/0:0)	-0.06	0.59	0.931
5'-Methylthioadenosine	0.04	0.593	0.931
LysoPC(0:0/18:0)	-0.07	0.597	0.934
LysoPC(20:4(5Z,8Z,11Z,14Z)/0:0)	-0.04	0.602	0.936
LysoPC(0:0/19:0)	-0.06	0.61	0.937
LysoPC(0:0/18:3)	-0.07	0.617	0.937
LysoPC(20:5(5Z,8Z,11Z,14Z,17Z)/0:0)	0.06	0.617	0.937
LysoPE(22:5(4Z,7Z,10Z,13Z,16Z)/0:0)	-0.04	0.618	0.937
LysoPC(18:4(6Z,9Z,12Z,15Z)/0:0)	-0.07	0.619	0.937
Succinyladenosine	-0.04	0.62	0.937
Octadecanedioic acid	-0.04	0.625	0.94
LysoPC(0:0/16:1)	0.06	0.633	0.945
LysoPE(0:0/18:2(9Z,12Z))	-0.06	0.634	0.945
alpha-Ketoisovaleric acid	-0.03	0.644	0.947
LysoPC(0:0/18:0(OH))	-0.07	0.645	0.947
3-Hydroxybutyrylcarnitine	0.16	0.651	0.947
LysoPC(16:0/0:0)	-0.01	0.652	0.947
7-Methylguanine	-0.03	0.653	0.947
LysoPC(18:2(9Z,12Z)/0:0)	-0.02	0.661	0.947
8-iso-PGE2 (pg/ml)	0.19	0.664	0.947
TxB2 (pg/ml)	0.23	0.667	0.947
Creatine	-0.06	0.68	0.947
LysoPC(O-16:0/0:0)	-0.05	0.681	0.947
Cholic acid	0.12	0.682	0.947
LysoPC(20:3(8Z,11Z,14Z)/0:0)	-0.03	0.684	0.947

L-Isoleucine	0.02	0.685	0.947
LysoPC(0:0/20:3)	-0.04	0.685	0.947
LysoPC(0:0/17:0)	-0.05	0.687	0.947
11(12)-EpETrE (pg/ml)	-0.06	0.696	0.947
Citric acid	0.01	0.697	0.947
Serotonin	-0.33	0.7	0.947
5(s)6(R)-LXA4 (pg/ml)	0.08	0.703	0.947
LysoPC(18:0/0:0)	-0.02	0.703	0.947
Bis(2-ethylhexyl)phthalate	-0.02	0.706	0.947
3-Hydroxyoctanoic acid	-0.05	0.709	0.947
Azelaic acid	0.04	0.713	0.947
LysoPE(20:4(5Z,8Z,11Z,14Z)/0:0)	-0.03	0.72	0.947
(+-)9-HETE (pg/ml)	-0.08	0.723	0.947
2-Hydroxycaproic acid	0.03	0.732	0.947
LysoPC(0:0/15:0)	-0.04	0.732	0.947
LysoPE(P-16:0/0:0)	-0.05	0.737	0.947
1-Methyladenosine	-0.03	0.741	0.947
LysoPE(0:0/20:5(5Z,8Z,11Z,14Z,17Z))	0.03	0.744	0.947
LysoPC(0:0/18:2(9Z,12Z))	-0.03	0.746	0.947
LysoPC(15:0/0:0)	0.03	0.753	0.947
LysoPE(0:0/22:6(4Z,7Z,10Z,13Z,16Z,19Z))	-0.03	0.753	0.947
LysoPC(17:0/0:0)	-0.04	0.755	0.947
Oxoglutaric acid	-0.02	0.757	0.947
2,3,4,5,6,7-Hexahydroxyheptanoic acid	0.01	0.759	0.947
LysoPC(19:0/0:0)	-0.05	0.759	0.947
Glutaric acid	-0.03	0.761	0.947
Stearic acid	0.06	0.762	0.947
Eicosapentaenoic acid	-0.05	0.763	0.947
Ketoleucine	0.03	0.763	0.947
Cytosine	0.04	0.768	0.947
LysoPC(20:0/0:0)	-0.04	0.775	0.947
LysoPE(0:0/16:0)	-0.02	0.776	0.947
Citrulline	-0.02	0.778	0.947
D-Glucoheptose	0.02	0.78	0.947
Palmitic acid	-0.05	0.78	0.947
L-Proline	0.02	0.786	0.947
Epinephrine	0.03	0.792	0.947
LysoPC(16:1(9Z)/0:0)	0.03	0.794	0.947
LysoPC(0:0/20:5)	0.03	0.796	0.947
D-Phenyllactic acid	0.04	0.796	0.947
Prolylhydroxyproline / Pyroglutamylvaline	-0.03	0.8	0.947
5-HETE (pg/ml)	-0.07	0.802	0.947
gamma-Aminobutyric acid	-0.02	0.803	0.947
5(6)-EpETrE (pg/ml)	-0.03	0.806	0.947
13-oxo-ODE (pg/ml)	0.05	0.808	0.947
LysoPE(P-18:0/0:0)	-0.04	0.81	0.947
9(S)-HODE (pg/ml)	0.05	0.811	0.947
8(9)-EpETrE (pg/ml)	-0.04	0.815	0.947

2-Hydroxy-3-methylpentanoic acid	-0.03	0.819	0.947
Sebacic acid	-0.02	0.82	0.947
12-HETE (pg/ml)	-0.05	0.821	0.947
Pantothenic acid	0.03	0.823	0.947
Isoleucyl-Leucine	-0.03	0.826	0.947
13-HODE (pg/ml)	0.04	0.833	0.947
(+-)11-HETE (pg/ml)	0.04	0.834	0.947
6-keto-PGF1a (pg/ml)	0.1	0.839	0.947
2-Hydroxydecanoate	-0.02	0.839	0.947
(R)-3-Hydroxy-hexadecanoic acid	0.03	0.839	0.947
12(13)-EpOME (pg/ml)	0.05	0.843	0.948
4-Hydroxybenzoic acid	0.04	0.851	0.951
17-HDOHE (pg/ml)	-0.03	0.851	0.951
2',4'-Dihydroxyacetophenone (Resorcinol monoacetate)	-0.06	0.86	0.952
9(10)-EpOME (pg/ml)	-0.04	0.861	0.952
LysoPC(0:0/14:0)	-0.02	0.862	0.952
12-Hydroxystearic acid	-0.02	0.865	0.952
Trigonelline	0.03	0.879	0.963
Isocitric acid	-0.01	0.88	0.963
LysoPE(18:0/0:0)	0.01	0.888	0.968
alpha-Chaconine	0.05	0.891	0.968
4-Hydroxyproline	-0.01	0.893	0.968
5-Hydroxyindoleacetic acid	-0.01	0.906	0.973
Methylmalonylcarnitine	0.01	0.907	0.973
PGD2 (pg/ml)	-0.04	0.907	0.973
gamma-Glutamyltryptophan	0.01	0.914	0.974
5-oxo-ETE (pg/ml)	0.02	0.92	0.974
Propionylcarnitine	0.01	0.923	0.974
N-Acetyl-L-methionine	0.01	0.924	0.974
Salicylic acid	-0.04	0.926	0.974
Adenosine	-0.02	0.926	0.974
LysoPE(0:0/20:4(5Z,8Z,11Z,14Z))	-0.01	0.931	0.976
LysoPE(20:5(5Z,8Z,11Z,14Z,17Z)/0:0)	0.01	0.946	0.981
3-(3-Hydroxyphenyl)-3-hydroxypropanoic acid / 4-Hydroxyphenyllactic acid	-0.01	0.947	0.981
(±)-2-Hydroxy-4-(methylthio)butanoic acid	-0.01	0.947	0.981
L-Phenylalanine	0	0.948	0.981
LysoPE(16:0/0:0)	0	0.958	0.983
L-Kynurenine	0	0.961	0.983
2-Hydroxyadipic acid / 3-Hydroxyadipic acid / 3-Hydroxymethylglutaric acid	0	0.965	0.983
Creatinine	0	0.966	0.983
Tetradecanedioic acid	0.01	0.968	0.983
Glyceric acid	0	0.972	0.983
Hexadecanedioic acid	0	0.973	0.983
LysoPE(22:6(4Z,7Z,10Z,13Z,16Z,19Z)/0:0)	0	0.974	0.983
LysoPC(14:0/0:0)	0	0.977	0.983

Oleamide	0	0.998	1
LysoPE(0:0/18:0)	0	1	1

Supplementary Table S5. The differences in relative metabolomic concentrations between smokers and non-smokers

variable	mean difference	p_value	adj_p_value
Serotonin	7.21	<0.001	<0.001
Malic acid	-0.26	<0.001	0.004
Cinnamoylglycine	-1.06	<0.001	0.005
Cholic acid	-0.86	<0.001	0.007
Tetradecanedioic acid	-0.64	<0.001	0.016
Hydroxyoctanoic acid	-0.45	<0.001	0.022
Quinic acid	0.63	0.001	0.035
(S)-3-Hydroxyisobutyric acid	-0.37	0.001	0.035
2,4-Dihydroxybenzoic acid / 2-Pyrocatechuic acid	0.59	0.001	0.035
Docosahexaenoic acid	-0.49	0.001	0.035
Trigonelline	0.63	0.001	0.035
Pimelic acid / 3-Methyladipic acid	-0.14	0.001	0.035
Sebacic acid	-0.25	0.002	0.042
Octadecanedioic acid	-0.27	0.002	0.045
L-Proline	0.19	0.002	0.046
Oxoglutaric acid	-0.19	0.003	0.052
AICAR	-0.28	0.003	0.052
Hexadecanedioic acid	-0.31	0.003	0.052
Tiglylcarnitine	-0.34	0.003	0.053
Hexose	0.27	0.003	0.055
Paraxanthine	0.65	0.004	0.055
Phenylacetylglutamine	-0.66	0.004	0.057
LysoPE(0:0/22:6(4Z,7Z,10Z,13Z,16Z,19Z))	-0.22	0.004	0.057
gamma-Glutamylalanine	0.32	0.004	0.057
L,L-Cyclo(leucylprolyl)	0.59	0.005	0.065
2-Hydroxyadipic acid / 3-Hydroxyadipic acid / 3-Hydroxymethylglutaric acid	-0.12	0.007	0.081
3-Methylcrotonylglycine / Tiglylglycine	-0.4	0.007	0.081
3-Hydroxycapric acid	-0.35	0.007	0.085
Citraconic acid	0.47	0.009	0.097
Indoleacetic acid	-0.35	0.01	0.103
alpha-Ketoisovaleric acid	-0.17	0.01	0.105
Cytosine	-0.32	0.012	0.123
13-HODE (pg/ml)	-0.52	0.013	0.123
9 10-DiHOME (pg/ml)	-0.55	0.014	0.134
Succinic acid	-0.13	0.015	0.135
Glyceric acid	-0.22	0.015	0.135
Ketoleucine	-0.23	0.016	0.138
gamma-Aminobutyric acid	-0.16	0.016	0.138
Caffeine	0.51	0.019	0.155
2-Hydroxydecanoate	-0.28	0.019	0.157
12 13-DiHOME (pg/ml)	-0.48	0.02	0.158
Tetradecanoylcarnitine	-0.36	0.02	0.158

2-Hydroxymyristoylcarnitine	-0.37	0.023	0.165
Dodecanoylcarnitine	-0.38	0.023	0.165
2-Hydroxycaproic acid	-0.19	0.023	0.165
14 15-DiHETrE (pg/ml)	0.19	0.024	0.168
LysoPE(22:6(4Z,7Z,10Z,13Z,16Z,19Z)/0:0)	-0.2	0.024	0.168
Uric acid	-0.13	0.025	0.169
9,12-Hexadecadienoylcarnitine	-0.34	0.027	0.172
4-Coumaryl alcohol	-0.66	0.027	0.172
3-Hydroxytetradecanoic acid	-0.17	0.028	0.172
L-Isoleucine	-0.13	0.029	0.172
Citrulline	0.17	0.029	0.172
9 10 13-TriHOME (pg/ml)	-0.38	0.029	0.172
4-Pyridoxic acid	-0.44	0.029	0.172
Epinephrine	0.2	0.03	0.172
Bilirubin	-0.3	0.032	0.183
cis-4-Decenoylcarnitine	-0.32	0.033	0.185
L-Acetylcarnitine	-0.21	0.034	0.186
Cervonylcarnitine	-0.26	0.036	0.195
3, 5-Tetradecadiencarnitine	-0.35	0.037	0.195
N-Acetyl-L-alanine	-0.09	0.038	0.198
Glutarylcarnitine	-0.2	0.038	0.198
alpha-Dimorphecolic acid	-0.26	0.039	0.198
alpha-Linolenic acid	-0.45	0.042	0.209
L-Palmitoylcarnitine	-0.27	0.045	0.218
trans-2-Dodecenoylcarnitine	-0.38	0.046	0.218
N-gamma-Glutamylglutamine	0.15	0.046	0.218
Eicosapentaenoic acid	-0.32	0.047	0.223
Decanoylcarnitine	-0.34	0.05	0.233
Indole-3-propionic acid	-0.63	0.054	0.248
Azelaic acid	-0.25	0.056	0.252
5'-Methylthioadenosine	-0.14	0.057	0.254
Docosapentaenoic acid (22n-3)	-0.4	0.059	0.257
Chenodeoxycholic acid	-0.48	0.06	0.257
9 12 13-TriHOME (pg/ml)	-0.52	0.06	0.257
L-Octanoylcarnitine	-0.3	0.062	0.261
3-Hydroxyisovalerylcarnitine	-0.2	0.064	0.265
Salicylic acid	0.79	0.065	0.268
Itaconic acid	0.19	0.068	0.276
9(10)-EpOME (pg/ml)	-0.35	0.07	0.276
LysoPC(0:0/22:5)	-0.14	0.071	0.276
(+-)11-HETE (pg/ml)	0.42	0.071	0.276
S-Adenosylhomocysteine	-0.25	0.072	0.276
LysoPC(12:0/0:0)	0.37	0.073	0.276
Succinyladenosine	0.15	0.074	0.276
Isovalerylcarnitine	-0.17	0.076	0.276
9(S)-HODE (pg/ml)	-0.41	0.077	0.276
2-Hydroxymyristic acid	-0.22	0.077	0.276
3-Hydroxybutyric acid	-0.41	0.077	0.276

Myristoleoylcarnitine	-0.32	0.079	0.276
3-(3-Hydroxyphenyl)-3-hydroxypropanoic acid / 4-Hydroxyphenyllactic acid	-0.13	0.079	0.276
9-Hexadecenoylcarnitine	-0.27	0.079	0.276
LysoPC(0:0/22:6)	-0.22	0.08	0.276
Linoleic acid	-0.28	0.081	0.276
6-trans-LTB4 (pg/ml)	0.62	0.085	0.282
D-Leucic acid	0.13	0.085	0.282
Linoleylcarnitine	-0.2	0.087	0.282
15-HETE (pg/ml)	0.3	0.087	0.282
LysoPC(22:6(4Z,7Z,10Z,13Z,16Z,19Z)/0:0)	-0.13	0.087	0.282
Oleic acid / Elaidic acid	-0.33	0.088	0.282
Pantothenic acid	-0.22	0.088	0.282
4-Hydroxybenzoic acid	0.39	0.091	0.284
LysoPC(0:0/17:0)	-0.2	0.091	0.284
PGD2 (pg/ml)	0.46	0.092	0.284
LysoPE(P-16:0/0:0)	-0.23	0.094	0.288
PGE2 (pg/ml)	0.68	0.096	0.288
3-Hydroxydodecanoic acid	-0.23	0.096	0.288
LysoPC(16:0(OH)/0:0)	-0.21	0.097	0.29
3a,7b,12a-Trihydroxyoxocholanyl-Glycine	-0.24	0.104	0.305
Glycocholic acid	-0.37	0.104	0.305
13-oxo-ODE (pg/ml)	-0.37	0.105	0.306
LysoPC(17:0/0:0)	-0.21	0.108	0.309
L-Sorbose	-0.31	0.115	0.329
LysoPC(0:0/18:1(9Z))	0.09	0.117	0.33
Hippuric acid	-0.36	0.119	0.33
7-Methylguanine	0.09	0.119	0.33
LysoPC(15:0/0:0)	-0.16	0.12	0.33
3-Hydroxyisovaleric acid	-0.11	0.13	0.353
Dodecanedioic acid	-0.14	0.13	0.353
3-Carboxy-4-methyl-5-propyl-2-furanpropionic acid	-0.3	0.134	0.361
Asymmetric dimethylarginine	0.13	0.138	0.365
3-Hydroxyoctadecenoylcarnitine	-0.26	0.138	0.365
4-Hydroxyproline	0.1	0.141	0.367
4-Acetamidobutanoic acid	-0.07	0.141	0.367
Indoxyl sulfate	-0.23	0.144	0.372
LysoPC(O-16:0/0:0)	-0.18	0.15	0.385
Pipecolic acid	-0.15	0.152	0.385
5(s)6(R)-LXA4 (pg/ml)	0.33	0.157	0.395
LysoPE(P-18:0/0:0)	-0.22	0.159	0.396
Valerylcarnitine	-0.17	0.16	0.396
LysoPC(0:0/20:2)	-0.1	0.166	0.404
8-iso-PGE2 (pg/ml)	0.52	0.168	0.404
6-keto-PGF1a (pg/ml)	0.59	0.169	0.404
LysoPC(0:0/15:0)	-0.17	0.169	0.404
3-Hydroxyoctanoic acid	-0.17	0.169	0.404

Kynurenic acid	-0.15	0.175	0.41
Threonic acid	-0.23	0.176	0.41
2-Hydroxylauroylcarnitine	-0.2	0.176	0.41
MG(18:1(9Z)/0:0/0:0)	0.18	0.177	0.41
Isoleucyl-Isoleucine	0.15	0.181	0.417
Theophylline	0.25	0.184	0.42
Capryloylglycine	-0.18	0.187	0.42
3-Methoxytyrosine	-0.09	0.191	0.42
11 12-DiHETrE (pg/ml)	0.14	0.191	0.42
Betaine	-0.05	0.191	0.42
Acetoacetic acid	-0.26	0.192	0.42
12(13)-EpOME (pg/ml)	-0.34	0.192	0.42
gamma-Glutamylmethionine	0.14	0.193	0.42
20-HETE (pg/ml)	-0.17	0.197	0.425
LysoPE(16:0/0:0)	-0.12	0.197	0.425
Deoxycholic acid	-0.31	0.199	0.425
Gamma-linolenyl arnitine	-0.16	0.207	0.437
Propionylcarnitine	-0.11	0.211	0.437
1-Aminocyclopropanecarboxylic acid	-0.07	0.212	0.437
Oleoylcarnitine	-0.16	0.212	0.437
LysoPE(0:0/16:0)	-0.1	0.212	0.437
8(S)-HETE (pg/ml)	0.28	0.212	0.437
Prolylhydroxyproline / Pyroglutamylvaline	0.13	0.215	0.44
3-Methylglutarylcarnitine	-0.16	0.218	0.442
L-Kynurenine	-0.1	0.22	0.444
Methylmalonylcarnitine	-0.09	0.222	0.444
2-Hydroxyhexadecanoic acid	-0.16	0.222	0.444
LysoPE(20:4(5Z,8Z,11Z,14Z)/0:0)	-0.09	0.232	0.461
2',4'-Dihydroxyacetophenone (Resorcinol monoacetate)	-0.4	0.234	0.461
Phenylalanylisoleucine	0.1	0.243	0.475
15(S)-HETrE (pg/ml)	0.26	0.244	0.475
5-HETE (pg/ml)	0.41	0.253	0.489
Uridine/Pseudouridine	-0.1	0.254	0.489
Glycoursodeoxycholic acid	0.25	0.259	0.494
Phenylalanyltryptophan / Tryptophyl-Phenylalanine	0.13	0.268	0.505
N-Acetylserine	-0.1	0.268	0.505
12-HETE (pg/ml)	0.24	0.27	0.505
Phenylalanylphenylalanine	0.13	0.271	0.505
N-Acetyl-L-methionine	0.08	0.276	0.51
(5Z,8Z,11Z,14Z,17Z)-Icosa-5,8,11,14,17-pentaenoylcarnitine	-0.14	0.276	0.51
Arachidonic acid	-0.16	0.285	0.522
Thyroxine	-0.1	0.286	0.522
LysoPE(O-16:0/0:0)	-0.14	0.289	0.524
Deoxycholic acid glycine conjugate	-0.25	0.298	0.538
Adipic acid	-0.07	0.3	0.538

Creatine	-0.14	0.302	0.538
Uracil	-0.1	0.303	0.538
LysoPC(0:0/19:0)	-0.11	0.309	0.546
alpha-Chaconine	-0.34	0.311	0.547
2-Methoxybenzoic acid	-0.08	0.315	0.551
L-Phenylalanine	-0.03	0.324	0.562
L-Methionine	-0.08	0.329	0.57
(2E)-3-(4-hydroxy-3-methoxyphenyl)prop-2-enal	-0.2	0.333	0.573
Cortisol / hydrocortisone	-0.16	0.344	0.588
12(S)-HEPE (pg/ml)	0.21	0.352	0.599
LysoPC(20:2(11Z,14Z)/0:0)	-0.11	0.354	0.599
5-Hydroxyindoleacetic acid	-0.08	0.363	0.612
gamma-Glutamylisoleucine	0.13	0.369	0.617
LysoPC(22:4(7Z,10Z,13Z,16Z)/0:0)	-0.08	0.37	0.617
Guanine	-0.07	0.377	0.623
2-Hydroxy-3-methylbutyric acid	-0.13	0.378	0.623
Phenylalanylproline	-0.11	0.381	0.625
Citric acid	0.02	0.391	0.635
Phenylalanylglutamic acid / gamma-Glutamylphenylalanine	0.06	0.392	0.635
Theobromine	0.17	0.393	0.635
Icosa-8,11,14-trienoylcarnitine	-0.1	0.396	0.638
LysoPC(19:0/0:0)	-0.14	0.4	0.64
LysoPE(0:0/20:4(5Z,8Z,11Z,14Z))	-0.06	0.402	0.64
(+-)9-HETE (pg/ml)	0.25	0.406	0.64
1-Methyladenosine	-0.06	0.406	0.64
LysoPE(O-18:0/0:0)	-0.11	0.408	0.64
Levonorgestrel	-0.12	0.414	0.646
2-Hydroxy-3-methylpentanoic acid	-0.1	0.417	0.646
LysoPC(20:1(11Z)/0:0)	-0.1	0.418	0.646
Leucyl-Aspartate / gamma-Glutamylvaline	0.14	0.422	0.65
N-Formyl-L-methionine	-0.04	0.424	0.65
LTB4 (pg/ml)	0.34	0.431	0.653
Docosapentaenoylcarnitine	-0.09	0.431	0.653
3-Hydroxybutyrylcarnitine	0.28	0.432	0.653
(7Z,10Z,13Z,16Z)-Docosatetraenoylcarnitine	-0.09	0.438	0.659
(11Z)-Eicoseneoylcarnitine	-0.1	0.444	0.665
LysoPC(0:0/20:4)	-0.07	0.452	0.674
LysoPC(P-18:0/0:0)	-0.1	0.464	0.688
LysoPC(16:0/0:0)	-0.02	0.465	0.688
Lenticin	0.29	0.476	0.7
L-Leucine	-0.03	0.484	0.708
Undecanedioic acid	0.05	0.491	0.716
Cortisone	-0.09	0.504	0.727
LysoPC(0:0/20:1)	-0.05	0.506	0.727
LysoPE(18:0/0:0)	-0.07	0.508	0.727
LysoPE(0:0/18:3(6Z,9Z,12Z))	0.08	0.509	0.727

Stearoylcarnitine	-0.09	0.512	0.727
MG(18:2(9Z,12Z)/0:0/0:0)	-0.07	0.512	0.727
Ursodeoxycholic acid	-0.13	0.524	0.738
LysoPC(0:0/20:5)	-0.09	0.527	0.738
4-Hydroxyhippuric acid	-0.1	0.527	0.738
N2,N2-Dimethylguanosine	0.06	0.529	0.738
LysoPC(0:0/16:1)	0.07	0.533	0.741
Palmitic acid	-0.1	0.542	0.749
L-Lysine	-0.04	0.544	0.749
LysoPC(20:5(5Z,8Z,11Z,14Z,17Z)/0:0)	-0.07	0.561	0.768
LysoPC(0:0/18:0(OH))	-0.09	0.563	0.768
Adenosine	0.1	0.572	0.776
LysoPE(22:4(7Z,10Z,13Z,16Z)/0:0)	-0.05	0.573	0.776
LysoPC(18:2(9Z,12Z)/0:0)	-0.03	0.592	0.794
LysoPC(20:4(5Z,8Z,11Z,14Z)/0:0)	-0.03	0.593	0.794
Xanthine	-0.06	0.597	0.794
N-(3-acetamidopropyl)pyrrolidin-2-one	-0.05	0.598	0.794
12-Hydroxystearic acid	0.06	0.599	0.794
L-Tyrosine	-0.03	0.604	0.795
Creatinine	0.03	0.606	0.795
Niacinamide	-0.05	0.606	0.795
LysoPE(0:0/18:0)	-0.04	0.609	0.795
LysoPC(0:0/16:0)	-0.04	0.618	0.801
TxB2 (pg/ml)	0.26	0.619	0.801
1,11-Undecanedicarboxylic acid	0.04	0.625	0.804
Suberic acid	-0.03	0.628	0.804
LysoPC(14:1(9Z)/0:0)	0.07	0.63	0.804
Pyroglutamic acid	-0.03	0.631	0.804
5 6-DiHETrE (pg/ml)	0.07	0.637	0.806
Arachidonoylcarnitine	-0.06	0.639	0.806
LysoPE(18:3(9Z,12Z,15Z)/0:0)	0.07	0.64	0.806
LysoPC(0:0/22:4)	-0.04	0.644	0.807
LysoPC(0:0/18:3)	0.06	0.648	0.807
Methylguanosine	0.02	0.648	0.807
LysoPC(16:1(9Z)/0:0)	0.05	0.663	0.821
LysoPC(18:3(9Z,12Z,15Z)/0:0)	0.06	0.665	0.821
11(12)-EpETrE (pg/ml)	-0.06	0.683	0.841
Guanosine	0.06	0.691	0.845
Bis(2-ethylhexyl)phthalate	-0.02	0.691	0.845
Hexanoylcarnitine	-0.06	0.694	0.845
gamma-Glutamylleucine	0.05	0.701	0.85
12-OxoETE (pg/ml)	-0.08	0.705	0.852
5-oxo-ETE (pg/ml)	0.1	0.713	0.859
LysoPC(20:3(8Z,11Z,14Z)/0:0)	-0.03	0.726	0.871
Isoleucyl-Leucine	0.03	0.739	0.877
Isobutyryl-L-carnitine	-0.05	0.74	0.877
Taurocholic acid	0.06	0.741	0.877
LysoPC(18:4(6Z,9Z,12Z,15Z)/0:0)	0.05	0.742	0.877

Oleamide	-0.04	0.753	0.886
LysoPE(20:5(5Z,8Z,11Z,14Z,17Z)/0:0)	-0.04	0.757	0.889
LysoPC(0:0/18:2(9Z,12Z))	-0.03	0.768	0.898
LysoPE(0:0/20:5(5Z,8Z,11Z,14Z,17Z))	-0.03	0.773	0.9
LysoPC(0:0/20:3)	-0.03	0.778	0.903
17-HDOHE (pg/ml)	0.06	0.781	0.903
LysoPC(14:0/0:0)	-0.04	0.792	0.913
Indolelactic acid	-0.02	0.798	0.913
2-Hydroxystearic acid	-0.06	0.798	0.913
LysoPE(18:2(9Z,12Z)/0:0)	-0.03	0.806	0.913
Sphingosine 1-phosphate	-0.02	0.806	0.913
(R)-3-Hydroxy-hexadecanoic acid	-0.03	0.807	0.913
Butyrylcarnitine	-0.03	0.81	0.913
Stearic acid	0.04	0.812	0.913
Acetaminophen	-0.16	0.821	0.92
Trimethylamine N-oxide	-0.03	0.843	0.941
Chenodeoxycholic acid glycine conjugate	-0.03	0.849	0.945
2,3,4,5,6,7-Hexahydroxyheptanoic acid	-0.01	0.857	0.946
D-Phenyllactic acid	-0.02	0.857	0.946
8(9)-EpETrE (pg/ml)	0.03	0.859	0.946
Glutaric acid	-0.01	0.867	0.952
Inosine	-0.02	0.876	0.958
Indoleacetaldehyde	0.01	0.88	0.958
LysoPC(18:1(9Z)/0:0)	-0.01	0.882	0.958
(±)-2-Hydroxy-4-(methylthio)butanoic acid	0.01	0.889	0.962
8 9-DiHETrE (pg/ml)	0.01	0.895	0.962
LysoPE(22:5(4Z,7Z,10Z,13Z,16Z)/0:0)	-0.01	0.9	0.962
Lithocholic acid glycine conjugate	0.02	0.901	0.962
LysoPC(20:0/0:0)	0.02	0.902	0.962
Homo-L-arginine	-0.01	0.903	0.962
LysoPC(22:5(4Z,7Z,10Z,13Z,16Z)/0:0)	-0.01	0.913	0.97
15-oxo-ETE (pg/ml)	-0.02	0.923	0.971
gamma-Glutamyltyrosine	0.01	0.933	0.971
Taurochenodesoxycholic acid	0.01	0.935	0.971
LysoPE(0:0/18:2(9Z,12Z))	0.01	0.935	0.971
5(6)-EpETrE (pg/ml)	-0.01	0.937	0.971
L-Tryptophan	0	0.937	0.971
LysoPE(18:1(9Z)/0:0)	0.01	0.938	0.971
LysoPE(0:0/18:1(9Z))	-0.01	0.938	0.971
LysoPC(18:0/0:0)	0	0.942	0.972
Indole-3-carboxaldehyde	0.01	0.951	0.978
2-Octenoylcarnitine	0.01	0.957	0.979
Isocitric acid	0	0.958	0.979
LysoPC(18:0(OH)/0:0)	0.01	0.963	0.98
Hypoxanthine	-0.01	0.967	0.98
3beta,7alpha-Dihydroxy-5-cholestenoate	0	0.968	0.98
gamma-Glutamyltryptophan	0	0.975	0.984
LysoPC(0:0/18:0)	0	0.979	0.985

LysoPC(0:0/14:0)	0	0.982	0.985
D-Glucoheptose	0	0.993	0.993

Supplementary Table S6. The differences in relative metabolomic concentrations between one episode of collagenous colitis or refractory collagenous colitis

variable	mean difference	p_value	adj_p_value
Phenylacetylglutamine	-0.67	0.006	0.999
Theobromine	0.65	0.012	0.999
(2E)-3-(4-hydroxy-3-methoxyphenyl)prop-2-enal	0.53	0.02	0.999
4-Acetamidobutanoic acid	-0.12	0.033	0.999
3-Carboxy-4-methyl-5-propyl-2-furanpropionic acid	0.46	0.042	0.999
L-Acetylcarnitine	-0.25	0.046	0.999
N-(3-acetamidopropyl)pyrrolidin-2-one	-0.24	0.052	0.999
3-Hydroxybutyric acid	-0.56	0.055	0.999
PGE2 (pg/ml)	1.16	0.066	0.999
D-Leucic acid	0.17	0.067	0.999
LysoPE(20:4(5Z,8Z,11Z,14Z)/0:0)	-0.17	0.067	0.999
13-HODE (pg/ml)	-0.44	0.069	0.999
N-Acetyl-L-alanine	-0.08	0.077	0.999
Hydroxyoctanoic acid	-0.27	0.086	0.999
Succinic acid	-0.1	0.088	0.999
11 12-DiHETrE (pg/ml)	-0.2	0.088	0.999
(7Z,10Z,13Z,16Z)-Docosatetraenoylcarnitine	-0.25	0.093	0.999
4-Coumaryl alcohol	-0.51	0.1	0.999
4-Pyridoxic acid	0.43	0.102	0.999
Icosa-8,11,14-trienoylcarnitine	-0.25	0.107	0.999
(±)-2-Hydroxy-4-(methylthio)butanoic acid	-0.16	0.11	0.999
LysoPE(0:0/20:4(5Z,8Z,11Z,14Z))	-0.15	0.111	0.999
9(S)-HODE (pg/ml)	-0.4	0.112	0.999
MG(18:1(9Z)/0:0/0:0)	0.26	0.116	0.999
Isovalerylcarnitine	-0.18	0.118	0.999
Acetaminophen	-1.51	0.12	0.999
15(S)-HETrE (pg/ml)	-0.36	0.133	0.999
2,4-Dihydroxybenzoic acid / 2-Pyrocatechuic acid	-0.34	0.133	0.999
alpha-Linolenic acid	-0.39	0.133	0.999
8-iso-PGE2 (pg/ml)	0.71	0.14	0.999
Cinnamoylglycine	-0.45	0.141	0.999
Tetradecanoylcarnitine	-0.28	0.143	0.999
Glyceric acid	0.16	0.144	0.999
LysoPC(22:4(7Z,10Z,13Z,16Z)/0:0)	-0.15	0.146	0.999
Propionylcarnitine	-0.17	0.15	0.999
LysoPC(20:4(5Z,8Z,11Z,14Z)/0:0)	-0.11	0.156	0.999
Methylmalonylcarnitine	-0.12	0.16	0.999
7-Methylguanine	-0.1	0.16	0.999
Deoxycholic acid	-0.5	0.164	0.999
Indoxyl sulfate	-0.26	0.165	0.999
LTB4 (pg/ml)	-0.61	0.174	0.999

Bis(2-ethylhexyl)phthalate	-0.1	0.177	0.999
Indoleacetaldehyde	0.11	0.177	0.999
2-Hydroxydecanoate	-0.19	0.178	0.999
2-Hydroxy-3-methylbutyric acid	-0.24	0.179	0.999
Uric acid	-0.09	0.18	0.999
Arachidonoylcarnitine	-0.21	0.181	0.999
3-Methoxytyrosine	-0.12	0.182	0.999
9-Hexadecenoylcarnitine	-0.26	0.183	0.999
3-Hydroxyoctadecenoylcarnitine	-0.31	0.185	0.999
Gamma-linolenyl arnitine	-0.23	0.185	0.999
L-Palmitoylcarnitine	-0.24	0.189	0.999
12-Hydroxystearic acid	0.19	0.192	0.999
Linoleylcarnitine	-0.22	0.196	0.999
8(S)-HETE (pg/ml)	-0.29	0.197	0.999
LysoPC(0:0/20:3)	-0.17	0.198	0.999
LysoPC(20:3(8Z,11Z,14Z)/0:0)	-0.12	0.207	0.999
Hippuric acid	-0.29	0.216	0.999
LysoPC(0:0/20:4)	-0.13	0.217	0.999
L-Sorbose	-0.25	0.223	0.999
3-(3-Hydroxyphenyl)-3-hydroxypropanoic acid / 4-Hydroxyphenyllactic acid	-0.11	0.227	0.999
trans-2-Dodecenoylcarnitine	-0.28	0.227	0.999
Itaconic acid	-0.18	0.228	0.999
Acetoacetic acid	-0.3	0.234	0.999
Prolylhydroxyproline / Pyroglutamylvaline	0.18	0.236	0.999
Deoxycholic acid glycine conjugate	-0.42	0.237	0.999
Oleoylcarnitine	-0.21	0.237	0.999
5(s)6(R)-LXA4 (pg/ml)	0.3	0.249	0.999
20-HETE (pg/ml)	-0.2	0.25	0.999
Serotonin	1.18	0.254	0.999
Cortisone	-0.22	0.256	0.999
AICAR	-0.13	0.257	0.999
D-Glucoheptose	-0.09	0.259	0.999
3-Hydroxycapric acid	-0.17	0.263	0.999
gamma-Glutamylalanine	0.17	0.265	0.999
2-Octenoylcarnitine	-0.23	0.266	0.999
12(S)-HEPE (pg/ml)	0.31	0.274	0.999
11(12)-EpETrE (pg/ml)	-0.21	0.276	0.999
alpha-Dimorphecolic acid	-0.18	0.281	0.999
LysoPE(22:4(7Z,10Z,13Z,16Z)/0:0)	-0.09	0.283	0.999
2-Hydroxymyristoylcarnitine	-0.22	0.283	0.999
9(10)-EpOME (pg/ml)	-0.3	0.286	0.999
Stearic acid	0.24	0.286	0.999
TxB2 (pg/ml)	0.77	0.287	0.999
Hexose	-0.11	0.299	0.999
Epinephrine	-0.11	0.3	0.999
Levonorgestrel	-0.21	0.302	0.999
9,12-Hexadecadienoylcarnitine	-0.21	0.306	0.999

gamma-Aminobutyric acid	-0.08	0.314	0.999
3, 5-Tetradecadiencarnitine	-0.22	0.323	0.999
Quinic acid	-0.24	0.324	0.999
Oleic acid / Elaidic acid	-0.23	0.327	0.999
2',4'-Dihydroxyacetophenone (Resorcinol monoacetate)	0.41	0.329	0.999
12 13-DiHOME (pg/ml)	-0.24	0.333	0.999
LysoPC(0:0/20:2)	-0.1	0.334	0.999
Kynurenic acid	-0.14	0.335	0.999
LysoPE(0:0/22:6(4Z,7Z,10Z,13Z,16Z,19Z))	-0.1	0.343	0.999
Pantothenic acid	0.12	0.349	0.999
S-Adenosylhomocysteine	-0.16	0.352	0.999
Butyrylcarnitine	-0.16	0.353	0.999
LysoPC(0:0/22:4)	-0.1	0.354	0.999
5'-Methylthioadenosine	-0.09	0.355	0.999
LysoPC(0:0/16:1)	-0.12	0.362	0.999
Tiglylcarnitine	-0.13	0.365	0.999
Tetradecanedioic acid	-0.18	0.366	0.999
Azelaic acid	0.14	0.368	0.999
Salicylic acid	-0.49	0.373	0.999
2-Hydroxystearic acid	0.24	0.373	0.999
(S)-3-Hydroxyisobutyric acid	-0.12	0.374	0.999
Myristoleoylcarnitine	-0.21	0.377	0.999
LysoPC(16:1(9Z)/0:0)	-0.12	0.38	0.999
3-Hydroxyisovalerylcarnitine	-0.12	0.38	0.999
L-Tryptophan	0.06	0.38	0.999
alpha-Ketoisovaleric acid	-0.06	0.383	0.999
14 15-DiHETrE (pg/ml)	-0.09	0.387	0.999
3a,7b,12a-Trihydroxyoxocholanyl-Glycine	0.18	0.387	0.999
Uridine/Pseudouridine	-0.09	0.391	0.999
12-OxoETE (pg/ml)	-0.19	0.391	0.999
gamma-Glutamyltyrosine	-0.09	0.394	0.999
4-Hydroxyproline	0.07	0.394	0.999
Octadecanedioic acid	-0.08	0.396	0.999
Dodecanoylcarnitine	-0.18	0.397	0.999
(11Z)-Eicoseneoylcarnitine	-0.14	0.397	0.999
2-Hydroxy-3-methylpentanoic acid	-0.12	0.399	0.999
Dodecanedioic acid	-0.09	0.404	0.999
Pimelic acid / 3-Methyladipic acid	-0.05	0.412	0.999
L-Tyrosine	-0.07	0.412	0.999
Citraconic acid	-0.18	0.416	0.999
Leucyl-Aspartate / gamma-Glutamylvaline	0.16	0.422	0.999
Glycoursodeoxycholic acid	-0.21	0.422	0.999
1,11-Undecanedicarboxylic acid	0.08	0.427	0.999
Xanthine	0.12	0.431	0.999
Phenylalanylphenylalanine	0.1	0.432	0.999
Taurochenodesoxycholic acid	-0.12	0.434	0.999
2-Hydroxylauroylcarnitine	-0.14	0.434	0.999

Linoleic acid	-0.15	0.445	0.999
(R)-3-Hydroxy-hexadecanoic acid	0.11	0.448	0.999
LysoPC(18:0(OH)/0:0)	0.1	0.455	0.999
3-Methylglutarylcarnitine	-0.13	0.458	0.999
8 9-DiHETrE (pg/ml)	-0.1	0.461	0.999
1-Aminocyclopropanecarboxylic acid	0.05	0.465	0.999
3-Hydroxydodecanoic acid	-0.12	0.467	0.999
Bilirubin	-0.13	0.468	0.999
Indoleacetic acid	-0.1	0.469	0.999
Adenosine	0.14	0.47	0.999
1-Methyladenosine	-0.06	0.471	0.999
(+-)9-HETE (pg/ml)	-0.23	0.472	0.999
cis-4-Decenoylcarnitine	-0.14	0.473	0.999
9 10-DiHOME (pg/ml)	-0.21	0.482	0.999
5(6)-EpETrE (pg/ml)	-0.13	0.484	0.999
LysoPE(22:6(4Z,7Z,10Z,13Z,16Z,19Z)/0:0)	-0.08	0.485	0.999
Ursodeoxycholic acid	-0.19	0.487	0.999
gamma-Glutamylmethionine	0.09	0.488	0.999
gamma-Glutamylleucine	0.11	0.503	0.999
D-Phenyllactic acid	0.1	0.509	0.999
LysoPC(0:0/18:1(9Z))	0.05	0.519	0.999
12(13)-EpOME (pg/ml)	-0.2	0.524	0.999
LysoPE(O-16:0/0:0)	-0.1	0.526	0.999
2-Hydroxyhexadecanoic acid	0.1	0.533	0.999
Isobutyryl-L-carnitine	-0.11	0.535	0.999
Sphingosine 1-phosphate	0.05	0.535	0.999
LysoPC(0:0/15:0)	0.09	0.538	0.999
N-Acetyl-L-methionine	-0.06	0.538	0.999
Phenylalanylglutamic acid / gamma-Glutamylphenylalanine	-0.06	0.541	0.999
2-Hydroxyadipic acid / 3-Hydroxyadipic acid / 3-Hydroxymethylglutaric acid	-0.03	0.543	0.999
Theophylline	0.14	0.545	0.999
LysoPC(0:0/18:0(OH))	0.11	0.548	0.999
Taurocholic acid	-0.14	0.548	0.999
LysoPE(P-16:0/0:0)	-0.1	0.552	0.999
3-Hydroxybutyrylcarnitine	0.26	0.553	0.999
Indole-3-propionic acid	-0.21	0.557	0.999
L-Lysine	0.04	0.56	0.999
Valerylcarnitine	-0.09	0.56	0.999
LysoPE(18:2(9Z,12Z)/0:0)	-0.09	0.56	0.999
N-Formyl-L-methionine	-0.04	0.562	0.999
gamma-Glutamylisoleucine	0.1	0.567	0.999
Phenylalanyltryptophan / Tryptophyl-Phenylalanine	-0.11	0.571	0.999
LysoPE(0:0/20:5(5Z,8Z,11Z,14Z,17Z))	0.08	0.573	0.999
LysoPC(15:0/0:0)	0.07	0.575	0.999
LysoPC(16:0/0:0)	-0.02	0.586	0.999

9 12 13-TriHOME (pg/ml)	-0.19	0.591	0.999
2-Hydroxymyristic acid	-0.08	0.591	0.999
Guanine	-0.05	0.599	0.999
Lithocholic acid glycine conjugate	0.09	0.601	0.999
Paraxanthine	0.13	0.602	0.999
Stearoylcarnitine	-0.09	0.603	0.999
LysoPC(20:2(11Z,14Z)/0:0)	-0.08	0.609	0.999
LysoPE(0:0/18:3(6Z,9Z,12Z))	-0.07	0.609	0.999
Inosine	0.07	0.611	0.999
MG(18:2(9Z,12Z)/0:0/0:0)	0.06	0.613	0.999
LysoPE(0:0/18:2(9Z,12Z))	-0.08	0.613	0.999
5-HETE (pg/ml)	-0.2	0.617	0.999
N2,N2-Dimethylguanosine	0.06	0.618	0.999
LysoPE(0:0/16:0)	0.06	0.618	0.999
LysoPC(0:0/20:5)	0.09	0.62	0.999
L-Methionine	-0.05	0.621	0.999
Pyroglutamic acid	-0.04	0.622	0.999
Hexanoylcarnitine	0.1	0.622	0.999
Chenodeoxycholic acid	0.18	0.622	0.999
Undecanedioic acid	0.04	0.626	0.999
Guanosine	-0.08	0.626	0.999
Lenticin	0.24	0.627	0.999
Docosapentaenoylcarnitine	-0.07	0.629	0.999
Cytosine	-0.07	0.631	0.999
LysoPC(O-16:0/0:0)	-0.08	0.639	0.999
LysoPC(12:0/0:0)	0.1	0.647	0.999
Suberic acid	0.02	0.652	0.999
N-Acetylserine	-0.05	0.657	0.999
9 10 13-TriHOME (pg/ml)	-0.1	0.658	0.999
13-oxo-ODE (pg/ml)	0.12	0.66	0.999
LysoPC(16:0(OH)/0:0)	0.07	0.668	0.999
Isoleucyl-Leucine	0.05	0.673	0.999
Glycocholic acid	0.12	0.675	0.999
(5Z,8Z,11Z,14Z,17Z)-Icosa-5,8,11,14,17-pentaenoylcarnitine	-0.07	0.677	0.999
15-oxo-ETE (pg/ml)	-0.1	0.678	0.999
LysoPC(18:0/0:0)	-0.03	0.679	0.999
PGD2 (pg/ml)	0.15	0.684	0.999
L-Proline	-0.03	0.697	0.999
2-Hydroxycaproic acid	-0.04	0.698	0.999
Creatine	0.07	0.698	0.999
3-Hydroxyoctanoic acid	-0.05	0.699	0.999
L-Phenylalanine	-0.02	0.703	0.999
LysoPE(20:5(5Z,8Z,11Z,14Z,17Z)/0:0)	0.06	0.705	0.999
LysoPC(20:0/0:0)	0.07	0.708	0.999
Isocitric acid	-0.01	0.709	0.999
Citrulline	-0.03	0.715	0.999
Adipic acid	-0.02	0.715	0.999

Pipecolic acid	0.04	0.717	0.999
Palmitic acid	0.07	0.718	0.999
Indole-3-carboxaldehyde	0.05	0.723	0.999
5 6-DiHETrE (pg/ml)	-0.06	0.725	0.999
LysoPC(0:0/16:0)	-0.04	0.725	0.999
LysoPC(20:5(5Z,8Z,11Z,14Z,17Z)/0:0)	0.05	0.726	0.999
4-Hydroxyhippuric acid	-0.06	0.728	0.999
Threonic acid	-0.07	0.732	0.999
Hypoxanthine	0.05	0.734	0.999
3-Hydroxytetradecanoic acid	0.03	0.744	0.999
LysoPC(0:0/19:0)	-0.04	0.744	0.999
LysoPE(18:0/0:0)	-0.04	0.75	0.999
L-Kynurenine	-0.03	0.758	0.999
Hexadecanedioic acid	-0.04	0.758	0.999
Cervonylcarnitine	-0.05	0.764	0.999
Trigonelline	-0.07	0.768	0.999
Docosahexaenoic acid	-0.06	0.769	0.999
LysoPC(0:0/18:0)	-0.05	0.773	0.999
LysoPE(0:0/18:0)	-0.03	0.776	0.999
LysoPC(20:1(11Z)/0:0)	-0.05	0.778	0.999
Ketoleucine	-0.03	0.782	0.999
2,3,4,5,6,7-Hexahydroxyheptanoic acid	-0.01	0.788	0.999
5-Hydroxyindoleacetic acid	-0.03	0.793	0.999
LysoPC(18:3(9Z,12Z,15Z)/0:0)	-0.04	0.794	0.999
Oleamide	-0.04	0.795	0.999
Docosapentaenoic acid (22n-3)	-0.07	0.796	0.999
Phenylalanylproline	-0.04	0.799	0.999
Asymmetric dimethylarginine	-0.03	0.807	0.999
Uracil	-0.03	0.809	0.999
LysoPE(18:3(9Z,12Z,15Z)/0:0)	-0.04	0.81	0.999
2-Methoxybenzoic acid	-0.02	0.817	0.999
3beta,7alpha-Dihydroxy-5-cholestenoate	0.04	0.819	0.999
12-HETE (pg/ml)	0.05	0.826	0.999
LysoPC(14:1(9Z)/0:0)	-0.03	0.826	0.999
Malic acid	-0.01	0.832	0.999
Creatinine	-0.01	0.839	0.999
Niacinamide	0.02	0.841	0.999
L,L-Cyclo(leucylprolyl)	0.05	0.841	0.999
LysoPC(14:0/0:0)	-0.03	0.845	0.999
Betaine	-0.01	0.85	0.999
Methylguanosine	-0.01	0.854	0.999
LysoPC(18:4(6Z,9Z,12Z,15Z)/0:0)	-0.04	0.856	0.999
LysoPC(18:2(9Z,12Z)/0:0)	0.01	0.857	0.999
LysoPC(0:0/17:0)	0.03	0.859	0.999
gamma-Glutamyltryptophan	-0.01	0.863	0.999
LysoPE(22:5(4Z,7Z,10Z,13Z,16Z)/0:0)	-0.02	0.867	0.999
Isoleucyl-Isoleucine	-0.02	0.872	0.999
L-Leucine	-0.01	0.874	0.999

N-gamma-Glutamylglutamine	0.01	0.879	0.999
Trimethylamine N-oxide	-0.03	0.881	0.999
Glutaric acid	0.01	0.883	0.999
Phenylalanylisoleucine	0.01	0.887	0.999
LysoPE(18:1(9Z)/0:0)	-0.02	0.889	0.999
alpha-Chaconine	-0.05	0.893	0.999
6-trans-LTB4 (pg/ml)	-0.05	0.894	0.999
Succinyladenosine	0.01	0.894	0.999
Glutarylcarnitine	0.02	0.9	0.999
Chenodeoxycholic acid glycine conjugate	0.03	0.905	0.999
Cholic acid	-0.04	0.905	0.999
LysoPC(19:0/0:0)	0.02	0.906	0.999
LysoPC(0:0/14:0)	0.02	0.906	0.999
LysoPE(O-18:0/0:0)	-0.02	0.908	0.999
LysoPE(P-18:0/0:0)	-0.02	0.909	0.999
Caffeine	0.02	0.915	0.999
3-Methylcrotonylglycine / Tiglylglycine	0.02	0.918	0.999
LysoPC(18:1(9Z)/0:0)	0.01	0.921	0.999
15-HETE (pg/ml)	-0.02	0.923	0.999
Indolelactic acid	-0.01	0.926	0.999
Sebacic acid	-0.01	0.926	0.999
5-oxo-ETE (pg/ml)	-0.03	0.927	0.999
Thyroxine	-0.01	0.928	0.999
Homo-L-arginine	-0.01	0.932	0.999
Arachidonic acid	-0.02	0.932	0.999
LysoPC(22:5(4Z,7Z,10Z,13Z,16Z)/0:0)	0.01	0.938	0.999
Citric acid	0	0.94	0.999
4-Hydroxybenzoic acid	0.02	0.943	0.999
LysoPC(0:0/22:5)	0.01	0.945	0.999
LysoPC(22:6(4Z,7Z,10Z,13Z,16Z,19Z)/0:0)	0.01	0.95	0.999
Capryloylglycine	-0.01	0.955	0.999
17-HDOHE (pg/ml)	0.01	0.956	0.999
LysoPC(0:0/20:1)	-0.01	0.956	0.999
8(9)-EpETrE (pg/ml)	-0.01	0.957	0.999
LysoPC(17:0/0:0)	-0.01	0.959	0.999
Cortisol / hydrocortisone	0.01	0.962	0.999
LysoPE(0:0/18:1(9Z))	0.01	0.968	0.999
LysoPC(0:0/18:2(9Z,12Z))	0	0.97	0.999
LysoPC(0:0/18:3)	0.01	0.971	0.999
L-Octanoylcarnitine	0.01	0.974	0.999
Decanoylcarnitine	-0.01	0.977	0.999
Eicosapentaenoic acid	-0.01	0.977	0.999
LysoPC(P-18:0/0:0)	0	0.977	0.999
6-keto-PGF1a (pg/ml)	-0.01	0.982	0.999
L-Isoleucine	0	0.987	0.999
3-Hydroxyisovaleric acid	0	0.99	0.999
LysoPE(16:0/0:0)	0	0.991	0.999
LysoPC(0:0/22:6)	0	0.993	0.999

(+-)11-HETE (pg/ml)	0	0.997	0.999
Oxoglutaric acid	0	0.999	0.999

Supplementary Table S7. The differences in relative metabolomic concentrations between patients with IBS-like symptoms or not in collagenous colitis

variable	mean difference	p_value	adj_p_value
Octadecanedioic acid	-0.25	0.004	0.759
LysoPE(O-18:0/0:0)	-0.44	0.008	0.759
Bis(2-ethylhexyl)phthalate	-0.2	0.008	0.759
Tiglylcarnitine	-0.37	0.009	0.759
LysoPE(P-18:0/0:0)	-0.47	0.014	0.765
3-Hydroxyisovaleric acid	-0.22	0.014	0.765
LysoPC(0:0/15:0)	-0.36	0.016	0.765
LysoPC(0:0/17:0)	-0.33	0.021	0.846
1-Aminocyclopropanecarboxylic acid	0.16	0.026	0.876
LysoPC(0:0/19:0)	-0.29	0.029	0.876
LysoPE(O-16:0/0:0)	-0.35	0.03	0.876
LysoPC(17:0/0:0)	-0.34	0.038	0.934
3-Hydroxyisovalerylcarnitine	-0.27	0.041	0.934
LysoPC(19:0/0:0)	-0.41	0.045	0.934
Cinnamoylglycine	-0.6	0.052	0.934
LysoPE(P-16:0/0:0)	-0.31	0.052	0.934
LysoPC(0:0/14:0)	-0.31	0.054	0.934
Phenylalanylphenylalanine	0.25	0.056	0.934
LysoPC(14:0/0:0)	-0.31	0.056	0.934
Bilirubin	-0.32	0.061	0.934
LysoPC(0:0/18:0)	-0.3	0.061	0.934
LysoPC(14:1(9Z)/0:0)	-0.29	0.065	0.934
Isoleucyl-Leucine	0.23	0.07	0.934
LysoPC(15:0/0:0)	-0.23	0.071	0.934
N-Acetyl-L-methionine	0.18	0.073	0.934
4-Pyridoxic acid	0.5	0.077	0.934
LysoPC(20:0/0:0)	-0.31	0.079	0.934
Chenodeoxycholic acid glycine conjugate	-0.34	0.082	0.934
4-Coumaryl alcohol	-0.54	0.085	0.934
LysoPC(18:1(9Z)/0:0)	-0.1	0.086	0.934
12-HETE (pg/ml)	-0.44	0.091	0.95
LysoPC(O-16:0/0:0)	-0.26	0.101	0.982
Deoxycholic acid glycine conjugate	-0.56	0.102	0.982
Indoleacetic acid	-0.23	0.105	0.982
(S)-3-Hydroxyisobutyric acid	-0.21	0.11	0.982
4-Acetamidobutanoic acid	0.09	0.111	0.982
LysoPC(18:0/0:0)	-0.12	0.114	0.982
LysoPC(0:0/20:4)	-0.16	0.125	0.982
3-Methylcrotonylglycine / Tiglylglycine	-0.26	0.143	0.982
20-HETE (pg/ml)	-0.24	0.147	0.982
gamma-Aminobutyric acid	-0.12	0.15	0.982
Methylguanosine	0.1	0.15	0.982
2-Hydroxy-3-methylbutyric acid	-0.25	0.152	0.982
Pantothenic acid	0.18	0.153	0.982

2',4'-Dihydroxyacetophenone (Resorcinol monoacetate)	0.57	0.154	0.982
LysoPC(16:0/0:0)	-0.05	0.154	0.982
7-Methylguanine	0.1	0.156	0.982
LysoPC(0:0/18:1(9Z))	-0.11	0.16	0.982
S-Adenosylhomocysteine	-0.25	0.163	0.982
LysoPC(20:1(11Z)/0:0)	-0.23	0.164	0.982
LysoPC(0:0/16:0)	-0.14	0.171	0.982
LTB4 (pg/ml)	-0.64	0.179	0.982
LysoPC(20:5(5Z,8Z,11Z,14Z,17Z)/0:0)	-0.2	0.19	0.982
MG(18:2(9Z,12Z)/0:0/0:0)	-0.15	0.192	0.982
Cortisol / hydrocortisone	-0.29	0.194	0.982
LysoPC(12:0/0:0)	-0.28	0.194	0.982
LysoPC(0:0/20:5)	-0.22	0.195	0.982
(2E)-3-(4-hydroxy-3-methoxyphenyl)prop-2-enal	-0.3	0.195	0.982
Guanine	0.11	0.204	0.982
3a,7b,12a-Trihydroxyoxocholanyl-Glycine	-0.25	0.206	0.982
4-Hydroxyproline	0.1	0.21	0.982
Indole-3-carboxaldehyde	0.18	0.215	0.982
12(S)-HEPE (pg/ml)	-0.37	0.218	0.982
12(13)-EpOME (pg/ml)	0.36	0.219	0.982
9(10)-EpOME (pg/ml)	0.31	0.22	0.982
LysoPC(P-18:0/0:0)	-0.21	0.225	0.982
3-Hydroxybutyric acid	-0.34	0.227	0.982
alpha-Chaconine	0.47	0.228	0.982
LysoPC(20:2(11Z,14Z)/0:0)	-0.19	0.231	0.982
LysoPE(0:0/18:0)	-0.14	0.238	0.982
N-Acetylserine	0.13	0.239	0.982
Serotonin	1.19	0.243	0.982
Asymmetric dimethylarginine	-0.13	0.246	0.982
L-Methionine	0.11	0.25	0.982
gamma-Glutamylalanine	0.17	0.257	0.982
LysoPC(20:3(8Z,11Z,14Z)/0:0)	-0.11	0.258	0.982
LysoPE(18:0/0:0)	-0.15	0.261	0.982
Glycocholic acid	-0.3	0.265	0.982
LysoPE(20:5(5Z,8Z,11Z,14Z,17Z)/0:0)	-0.18	0.265	0.982
alpha-Linolenic acid	-0.29	0.267	0.982
LysoPC(0:0/20:1)	-0.12	0.273	0.982
Valerylcarnitine	-0.17	0.277	0.982
LysoPE(22:4(7Z,10Z,13Z,16Z)/0:0)	-0.09	0.287	0.982
Eicosapentaenoic acid	-0.22	0.289	0.982
L-Kynurenine	0.11	0.291	0.982
Tetradecanedioic acid	-0.2	0.292	0.982
Phenylacetylglutamine	-0.26	0.293	0.982
D-Leucic acid	0.09	0.293	0.982
N-Formyl-L-methionine	0.06	0.296	0.982
Methylmalonylcarnitine	0.09	0.296	0.982

Docosapentaenoylcarnitine	-0.16	0.299	0.982
LysoPC(18:4(6Z,9Z,12Z,15Z)/0:0)	-0.2	0.301	0.982
gamma-Glutamylmethionine	0.14	0.303	0.982
L-Tyrosine	-0.09	0.303	0.982
Adipic acid	-0.06	0.305	0.982
Guanosine	-0.16	0.306	0.982
(5Z,8Z,11Z,14Z,17Z)-Icosa-5,8,11,14,17-pentaenoylcarnitine	-0.18	0.309	0.982
Cholic acid	-0.36	0.31	0.982
LysoPC(0:0/20:3)	-0.13	0.312	0.982
LysoPC(18:2(9Z,12Z)/0:0)	-0.06	0.314	0.982
LysoPC(0:0/22:4)	-0.1	0.318	0.982
LysoPC(0:0/18:2(9Z,12Z))	-0.12	0.321	0.982
Pimelic acid / 3-Methyladipic acid	-0.06	0.321	0.982
LysoPC(16:1(9Z)/0:0)	-0.13	0.326	0.982
3-(3-Hydroxyphenyl)-3-hydroxypropanoic acid / 4-Hydroxyphenyllactic acid	-0.09	0.328	0.982
LysoPC(16:0(OH)/0:0)	0.15	0.328	0.982
LysoPC(0:0/22:6)	-0.14	0.331	0.982
Phenylalanylisoleucine	0.1	0.332	0.982
Tetradecanoylcarnitine	-0.19	0.333	0.982
TxB2 (pg/ml)	-0.66	0.336	0.982
Acetaminophen	0.9	0.336	0.982
Homo-L-arginine	0.12	0.348	0.986
LysoPC(18:3(9Z,12Z,15Z)/0:0)	-0.14	0.356	0.986
L-Proline	-0.08	0.357	0.986
5-Hydroxyindoleacetic acid	0.1	0.364	0.986
LysoPC(22:5(4Z,7Z,10Z,13Z,16Z)/0:0)	-0.13	0.364	0.986
LysoPE(0:0/20:5(5Z,8Z,11Z,14Z,17Z))	-0.12	0.365	0.986
Ursodeoxycholic acid	0.24	0.365	0.986
gamma-Glutamyltyrosine	-0.1	0.366	0.986
2-Octenoylcarnitine	0.2	0.369	0.986
Oleic acid / Elaidic acid	-0.2	0.374	0.986
Isoleucyl-Isoleucine	0.11	0.375	0.986
LysoPE(0:0/18:1(9Z))	-0.13	0.38	0.986
Epinephrine	-0.09	0.383	0.986
Xanthine	0.14	0.385	0.986
12 13-DiHOME (pg/ml)	0.2	0.395	0.986
Citrulline	-0.07	0.395	0.986
LysoPE(22:6(4Z,7Z,10Z,13Z,16Z,19Z)/0:0)	0.09	0.397	0.986
Inosine	-0.12	0.399	0.986
Cytosine	-0.12	0.4	0.986
L-Lysine	0.06	0.408	0.986
1,11-Undecanedicarboxylic acid	0.08	0.416	0.986
Stearic acid	0.17	0.418	0.986
13-HODE (pg/ml)	0.19	0.418	0.986
11 12-DiHETrE (pg/ml)	-0.1	0.419	0.986
Citric acid	-0.01	0.425	0.986

LysoPC(0:0/20:2)	-0.08	0.429	0.986
N-Acetyl-L-alanine	0.04	0.431	0.986
Isocitric acid	-0.03	0.434	0.986
Trigonelline	0.18	0.437	0.986
Pipecolic acid	0.08	0.438	0.986
9 12 13-TriHOME (pg/ml)	-0.27	0.443	0.986
Glyceric acid	-0.09	0.445	0.986
D-Phenyllactic acid	0.12	0.449	0.986
Glutaric acid	-0.06	0.453	0.986
LysoPC(0:0/22:5)	-0.06	0.466	0.986
Salicylic acid	-0.38	0.466	0.986
Trimethylamine N-oxide	0.15	0.468	0.986
Taurochenodesoxycholic acid	0.11	0.47	0.986
Arachidonoylcarnitine	-0.11	0.471	0.986
3-Hydroxyoctanoic acid	0.1	0.472	0.986
Sphingosine 1-phosphate	-0.05	0.475	0.986
Succinyladenosine	0.07	0.483	0.986
LysoPC(20:4(5Z,8Z,11Z,14Z)/0:0)	-0.05	0.49	0.986
LysoPC(0:0/18:3)	-0.11	0.49	0.986
Icosa-8,11,14-trienoylcarnitine	-0.11	0.495	0.986
L-Acetylcarnitine	-0.09	0.495	0.986
LysoPC(22:6(4Z,7Z,10Z,13Z,16Z,19Z)/0:0)	-0.06	0.497	0.986
Isovalerylcarnitine	-0.08	0.497	0.986
Succinic acid	0.04	0.498	0.986
2-Hydroxymyristic acid	-0.1	0.499	0.986
L-Palmitoylcarnitine	-0.12	0.499	0.986
Stearoylcarnitine	-0.12	0.499	0.986
8-iso-PGE2 (pg/ml)	-0.32	0.5	0.986
AICAR	0.07	0.501	0.986
Phenylalanyltryptophan / Tryptophyl-Phenylalanine	0.12	0.507	0.986
Propionylcarnitine	-0.08	0.515	0.986
Docosahexaenoic acid	-0.12	0.515	0.986
LysoPE(18:1(9Z)/0:0)	-0.1	0.519	0.986
4-Hydroxyhippuric acid	0.11	0.521	0.986
alpha-Ketoisovaleric acid	-0.05	0.526	0.986
Hypoxanthine	-0.1	0.532	0.986
Chenodeoxycholic acid	0.22	0.534	0.986
Hexose	-0.07	0.535	0.986
8 9-DiHETrE (pg/ml)	-0.08	0.538	0.986
3-Hydroxybutyrylcarnitine	0.27	0.539	0.986
Acetoacetic acid	-0.15	0.54	0.986
12-Hydroxystearic acid	0.08	0.541	0.986
9 10-DiHOME (pg/ml)	0.17	0.546	0.986
Cervonylcarnitine	-0.1	0.546	0.986
L-Phenylalanine	0.02	0.55	0.987
3-Methoxytyrosine	0.06	0.553	0.987
3-Hydroxytetradecanoic acid	-0.05	0.556	0.987

9 10 13-TriHOME (pg/ml)	0.13	0.564	0.991
15(S)-HETrE (pg/ml)	0.14	0.564	0.991
2-Methoxybenzoic acid	0.06	0.574	0.996
3-Methylglutarylcarnitine	0.1	0.575	0.996
2-Hydroxystearic acid	0.14	0.585	0.996
Lenticin	0.27	0.59	0.996
N-(3-acetamidopropyl)pyrrolidin-2-one	-0.07	0.591	0.996
3-Hydroxydodecanoic acid	-0.09	0.595	0.996
3-Hydroxycapric acid	-0.08	0.596	0.996
gamma-Glutamylisoleucine	0.1	0.598	0.996
Creatine	0.09	0.605	0.996
LysoPC(0:0/16:1)	-0.07	0.61	0.996
6-keto-PGF1a (pg/ml)	0.28	0.611	0.996
LysoPE(0:0/20:4(5Z,8Z,11Z,14Z))	-0.05	0.619	0.996
Deoxycholic acid	-0.17	0.621	0.996
Sebacic acid	-0.05	0.624	0.996
2,4-Dihydroxybenzoic acid / 2-Pyrocatechuic acid	-0.11	0.625	0.996
6-trans-LTB4 (pg/ml)	-0.18	0.635	0.996
5-oxo-ETE (pg/ml)	0.13	0.64	0.996
12-OxoETE (pg/ml)	-0.11	0.641	0.996
L-Isoleucine	0.04	0.643	0.996
N-gamma-Glutamylglutamine	0.04	0.648	0.996
Phenylalanylglutamic acid / gamma-Glutamylphenylalanine	0.04	0.656	0.996
LysoPE(18:2(9Z,12Z)/0:0)	-0.07	0.657	0.996
1-Methyladenosine	0.04	0.659	0.996
13-oxo-ODE (pg/ml)	0.12	0.66	0.996
Betaine	-0.02	0.662	0.996
Linoleic acid	-0.08	0.669	0.996
11(12)-EpETrE (pg/ml)	-0.08	0.67	0.996
2-Hydroxy-3-methylpentanoic acid	-0.06	0.671	0.996
Taurocholic acid	0.09	0.682	0.996
3-Carboxy-4-methyl-5-propyl-2-furanpropionic acid	-0.09	0.685	0.996
8(9)-EpETrE (pg/ml)	-0.09	0.692	0.996
5'-Methylthioadenosine	0.04	0.702	0.996
4-Hydroxybenzoic acid	0.09	0.708	0.996
Indoxyl sulfate	-0.07	0.708	0.996
Suberic acid	-0.02	0.712	0.996
trans-2-Dodecenoylcarnitine	0.09	0.715	0.996
Docosapentaenoic acid (22n-3)	-0.09	0.717	0.996
Oleamide	-0.06	0.718	0.996
Indole-3-propionic acid	-0.14	0.724	0.996
9,12-Hexadecadienoylcarnitine	0.07	0.738	0.996
14 15-DiHETrE (pg/ml)	-0.03	0.739	0.996
Palmitic acid	0.06	0.739	0.996
LysoPE(0:0/18:2(9Z,12Z))	-0.05	0.74	0.996

Indolelactic acid	-0.03	0.742	0.996
L-Sorbose	0.07	0.744	0.996
2-Hydroxylauroylcarnitine	-0.06	0.747	0.996
alpha-Dimorphecolic acid	-0.05	0.75	0.996
3, 5-Tetradecadiencarnitine	0.07	0.763	0.996
Cortisone	-0.05	0.763	0.996
Oxoglutaric acid	-0.02	0.763	0.996
Hippuric acid	0.07	0.764	0.996
Hydroxyoctanoic acid	-0.05	0.767	0.996
2-Hydroxyadipic acid / 3-Hydroxyadipic acid / 3-Hydroxymethylglutaric acid	0.02	0.767	0.996
Lithocholic acid glycine conjugate	-0.05	0.767	0.996
9(S)-HODE (pg/ml)	0.07	0.767	0.996
LysoPE(16:0/0:0)	-0.04	0.771	0.996
Capryloylglycine	0.05	0.772	0.996
2,3,4,5,6,7-Hexahydroxyheptanoic acid	-0.01	0.772	0.996
Glutarylcarnitine	0.04	0.775	0.996
8(S)-HETE (pg/ml)	0.06	0.779	0.996
Levonorgestrel	-0.06	0.78	0.996
Itaconic acid	-0.04	0.781	0.996
Kynurenic acid	0.04	0.79	0.996
Paraxanthine	0.06	0.792	0.996
cis-4-Decenoylcarnitine	0.05	0.793	0.996
(R)-3-Hydroxy-hexadecanoic acid	0.04	0.794	0.996
Pyroglutamic acid	0.02	0.796	0.996
Glycoursodeoxycholic acid	-0.07	0.796	0.996
2-Hydroxydecanoate	-0.03	0.798	0.996
Uric acid	0.02	0.805	0.996
Quinic acid	0.06	0.811	0.996
(+-)9-HETE (pg/ml)	-0.07	0.812	0.996
3-Hydroxyoctadecenoylcarnitine	-0.05	0.813	0.996
L-Octanoylcarnitine	0.05	0.816	0.996
LysoPE(22:5(4Z,7Z,10Z,13Z,16Z)/0:0)	-0.02	0.825	0.996
Hexanoylcarnitine	0.04	0.826	0.996
LysoPE(0:0/18:3(6Z,9Z,12Z))	0.03	0.827	0.996
Isobutyryl-L-carnitine	-0.04	0.828	0.996
Oleoylcarnitine	-0.04	0.83	0.996
9-Hexadecenoylcarnitine	-0.04	0.833	0.996
2-Hydroxymyristoylcarnitine	0.04	0.836	0.996
5(s)6(R)-LXA4 (pg/ml)	-0.05	0.836	0.996
gamma-Glutamyltryptophan	0.02	0.837	0.996
gamma-Glutamylleucine	0.03	0.843	0.996
15-HETE (pg/ml)	0.04	0.845	0.996
PGE2 (pg/ml)	0.11	0.85	0.996
Gamma-linolenyl arnitine	-0.03	0.852	0.996
Theobromine	0.05	0.858	0.996
2-Hydroxycaproic acid	0.02	0.866	0.996
Uridine/Pseudouridine	-0.02	0.87	0.996

Creatinine	0.01	0.87	0.996
5-HETE (pg/ml)	0.06	0.871	0.996
Malic acid	0.01	0.871	0.996
LysoPE(18:3(9Z,12Z,15Z)/0:0)	-0.03	0.877	0.996
Theophylline	0.03	0.88	0.996
Ketoleucine	-0.02	0.884	0.996
5(6)-EpETrE (pg/ml)	0.03	0.885	0.996
5 6-DiHETrE (pg/ml)	-0.03	0.886	0.996
(7Z,10Z,13Z,16Z)-Docosatetraenoylcarnitine	0.02	0.89	0.996
Threonic acid	-0.03	0.896	0.996
Uracil	0.01	0.9	0.996
Citraconic acid	0.03	0.907	0.996
LysoPE(0:0/22:6(4Z,7Z,10Z,13Z,16Z,19Z))	0.01	0.908	0.996
LysoPC(0:0/18:0(OH))	-0.02	0.913	0.996
D-Glucoheptose	0.01	0.915	0.996
Butyrylcarnitine	-0.02	0.918	0.996
LysoPE(0:0/16:0)	-0.01	0.92	0.996
Niacinamide	0.01	0.921	0.996
L-Leucine	0.01	0.923	0.996
Azelaic acid	0.01	0.925	0.996
Caffeine	-0.02	0.925	0.996
PGD2 (pg/ml)	0.03	0.927	0.996
Dodecanedioic acid	0.01	0.928	0.996
Myristoleoylcarnitine	0.02	0.93	0.996
2-Hydroxyhexadecanoic acid	0.01	0.93	0.996
Phenylalanylproline	-0.01	0.932	0.996
17-HDOHE (pg/ml)	0.02	0.934	0.996
Linoleylcarnitine	-0.01	0.938	0.996
MG(18:1(9Z)/0:0/0:0)	0.01	0.942	0.996
Decanoylcarnitine	0.02	0.946	0.996
Arachidonic acid	-0.01	0.948	0.996
N2,N2-Dimethylguanosine	-0.01	0.95	0.996
(+-)11-HETE (pg/ml)	-0.02	0.95	0.996
Prolylhydroxyproline / Pyroglutamylvaline	0.01	0.957	0.996
Dodecanoylcarnitine	-0.01	0.957	0.996
L-Tryptophan	0	0.959	0.996
Undecanedioic acid	0	0.959	0.996
LysoPC(18:0(OH)/0:0)	-0.01	0.96	0.996
LysoPE(20:4(5Z,8Z,11Z,14Z)/0:0)	0	0.963	0.996
Adenosine	-0.01	0.97	1
Thyroxine	0	0.973	1
Hexadecanedioic acid	0	0.979	1
15-oxo-ETE (pg/ml)	0.01	0.98	1
(11Z)-Eicoseneoylcarnitine	0	0.983	1
Leucyl-Aspartate / gamma-Glutamylvaline	0	0.984	1
LysoPC(22:4(7Z,10Z,13Z,16Z)/0:0)	0	0.989	1
Indoleacetaldehyde	0	0.996	1
(±)-2-Hydroxy-4-(methylthio)butanoic acid	0	0.996	1

3beta,7alpha-Dihydroxy-5-cholestenoate	0	0.999	1
L,L-Cyclo(leucylprolyl)	0	1	1

Supplementary Table S8. The differences in relative metabolomic concentrations between users of corticosteroids or not in collagenous colitis

variable	mean difference	p_value	adj_p_value
Icosa-8,11,14-trienoylcarnitine	-0.38	0.014	0.789
Cortisol / hydrocortisone	-0.62	0.017	0.789
3-Hydroxybutyric acid	-0.58	0.027	0.789
gamma-Glutamylleucine	0.37	0.031	0.789
Adipic acid	-0.12	0.033	0.789
gamma-Glutamylalanine	0.3	0.036	0.789
Chenodeoxycholic acid glycine conjugate	0.42	0.037	0.789
gamma-Glutamylmethionine	0.31	0.038	0.789
Arachidonoylcarnitine	-0.33	0.039	0.789
9 12 13-TriHOME (pg/ml)	0.73	0.039	0.789
11 12-DiHETrE (pg/ml)	-0.25	0.04	0.789
Indoleacetic acid	0.27	0.043	0.789
6-trans-LTB4 (pg/ml)	-0.7	0.044	0.789
Ursodeoxycholic acid	0.53	0.045	0.789
Theophylline	0.4	0.046	0.789
Lithocholic acid glycine conjugate	-0.35	0.047	0.789
(7Z,10Z,13Z,16Z)-Docosatetraenoylcarnitine	-0.3	0.05	0.789
gamma-Glutamylisoleucine	0.38	0.056	0.789
Cortisone	-0.41	0.058	0.789
Glycocholic acid	0.55	0.058	0.789
Sphingosine 1-phosphate	-0.15	0.059	0.789
Paraxanthine	0.39	0.064	0.789
Acetaminophen	1.86	0.064	0.789
3beta,7alpha-Dihydroxy-5-cholestenoate	-0.27	0.069	0.789
Deoxycholic acid glycine conjugate	0.64	0.074	0.789
PGE2 (pg/ml)	1.03	0.077	0.789
Leucyl-Aspartate / gamma-Glutamylvaline	0.39	0.077	0.789
Caffeine	0.35	0.084	0.789
Uric acid	0.11	0.089	0.789
Oleic acid / Elaidic acid	-0.38	0.089	0.789
1,11-Undecanedicarboxylic acid	0.19	0.089	0.789
Phenylalanylglutamic acid / gamma-Glutamylphenylalanine	0.15	0.091	0.789
L-Leucine	0.1	0.092	0.789
9 10-DiHOME (pg/ml)	0.5	0.093	0.789
L-Sorbose	0.35	0.094	0.789
(2E)-3-(4-hydroxy-3-methoxyphenyl)prop-2-enal	0.36	0.095	0.789
2-Hydroxymyristic acid	-0.24	0.1	0.789
Docosapentaenoylcarnitine	-0.24	0.104	0.789
Hippuric acid	0.38	0.105	0.789
3-Hydroxydodecanoic acid	-0.25	0.106	0.789
14 15-DiHETrE (pg/ml)	-0.17	0.107	0.789
gamma-Glutamyltyrosine	0.18	0.108	0.789

12 13-DiHOME (pg/ml)	0.41	0.108	0.789
AICAR	0.17	0.112	0.789
Asymmetric dimethylarginine	-0.18	0.118	0.789
9 10 13-TriHOME (pg/ml)	0.35	0.122	0.789
alpha-Dimorphecolic acid	-0.25	0.126	0.789
3-Hydroxyisovaleric acid	-0.14	0.127	0.789
LTB4 (pg/ml)	-0.8	0.128	0.789
Cinnamoylglycine	0.49	0.128	0.789
Ketoleucine	0.17	0.128	0.789
Adenosine	0.29	0.13	0.789
Inosine	0.22	0.133	0.789
Betaine	0.07	0.134	0.789
Decanoylcarnitine	-0.34	0.134	0.789
trans-2-Dodecenoylcarnitine	-0.36	0.141	0.789
Pipecolic acid	0.16	0.142	0.789
Dodecanoylcarnitine	-0.32	0.145	0.789
Butyrylcarnitine	0.24	0.145	0.789
Cervonylcarnitine	-0.22	0.147	0.789
2-Hydroxylauroylcarnitine	-0.25	0.148	0.789
Phenylalanylproline	0.21	0.158	0.794
Tetradecanoylcarnitine	-0.27	0.16	0.794
Uridine/Pseudouridine	-0.13	0.164	0.794
Linoleylcarnitine	-0.24	0.167	0.794
LysoPC(16:0(OH)/0:0)	0.2	0.168	0.794
L-Isoleucine	0.1	0.172	0.794
Indole-3-propionic acid	0.53	0.172	0.794
cis-4-Decenoylcarnitine	-0.27	0.173	0.794
L-Methionine	0.14	0.173	0.794
Salicylic acid	-0.75	0.18	0.794
Guanine	0.13	0.181	0.794
Homo-L-arginine	0.17	0.182	0.794
Myristoleoylcarnitine	-0.31	0.186	0.794
L-Phenylalanine	0.05	0.187	0.794
Tiglylcarnitine	-0.18	0.191	0.794
3, 5-Tetradecadiencarnitine	-0.29	0.196	0.794
2-Octenoylcarnitine	0.29	0.196	0.794
3-Hydroxytetradecanoic acid	-0.12	0.198	0.794
Linoleic acid	-0.26	0.198	0.794
Oleoylcarnitine	-0.23	0.199	0.794
9,12-Hexadecadienoylcarnitine	-0.28	0.205	0.794
MG(18:2(9Z,12Z)/0:0/0:0)	0.14	0.21	0.794
Lenticin	0.59	0.214	0.794
2-Hydroxyhexadecanoic acid	-0.2	0.214	0.794
Glutaric acid	-0.11	0.215	0.794
TxB2 (pg/ml)	0.84	0.215	0.794
Theobromine	0.31	0.22	0.794
N-Acetylserine	0.14	0.222	0.794
Threonic acid	-0.24	0.225	0.794

Methylmalonylcarnitine	0.12	0.229	0.794
9-Hexadecenoylcarnitine	-0.25	0.23	0.794
Glycoursodeoxycholic acid	0.31	0.232	0.794
Tetradecanedioic acid	0.25	0.232	0.794
Valerylcarnitine	0.19	0.235	0.794
L-Lysine	0.1	0.236	0.794
4-Pyridoxic acid	0.41	0.237	0.794
L-Acetylcarnitine	-0.15	0.241	0.8
3-Hydroxybutyrylcarnitine	0.48	0.246	0.807
N-Acetyl-L-methionine	0.11	0.256	0.823
L-Octanoylcarnitine	-0.25	0.258	0.823
LysoPC(0:0/18:1(9Z))	-0.1	0.26	0.823
Trimethylamine N-oxide	0.23	0.263	0.823
Indoxyl sulfate	0.21	0.263	0.823
8-iso-PGE2 (pg/ml)	0.56	0.273	0.846
2-Hydroxycaproic acid	0.11	0.279	0.853
Indoleacetaldehyde	0.08	0.281	0.853
2-Hydroxy-3-methylbutyric acid	-0.18	0.291	0.871
L-Tyrosine	0.1	0.296	0.871
Suberic acid	-0.06	0.298	0.871
Deoxycholic acid	0.38	0.3	0.871
L-Tryptophan	0.07	0.303	0.871
3a,7b,12a-Trihydroxyoxocholanyl-Glycine	0.21	0.304	0.871
Capryloylglycine	-0.18	0.305	0.871
Indolelactic acid	-0.09	0.308	0.871
LysoPE(0:0/20:5(5Z,8Z,11Z,14Z,17Z))	0.13	0.317	0.874
(11Z)-Eicoseneoylcarnitine	-0.17	0.319	0.874
alpha-Linolenic acid	-0.26	0.335	0.874
Dodecanedioic acid	0.11	0.337	0.874
Citrulline	-0.08	0.337	0.874
Acetoacetic acid	-0.25	0.338	0.874
Creatine	-0.17	0.338	0.874
Gamma-linolenyl arnitine	-0.17	0.339	0.874
S-Adenosylhomocysteine	0.18	0.342	0.874
Phenylacetylglutamine	0.22	0.345	0.874
N-gamma-Glutamylglutamine	0.09	0.347	0.874
alpha-Chaconine	0.36	0.352	0.874
LysoPE(22:5(4Z,7Z,10Z,13Z,16Z)/0:0)	0.1	0.353	0.874
3-Hydroxyoctadecenoylcarnitine	-0.21	0.356	0.874
4-Hydroxyproline	-0.08	0.359	0.874
Undecanedioic acid	0.08	0.359	0.874
1-Aminocyclopropanecarboxylic acid	0.07	0.36	0.874
Succinic acid	-0.05	0.362	0.874
15(S)-HETrE (pg/ml)	-0.21	0.362	0.874
LysoPC(0:0/22:4)	-0.1	0.363	0.874
Propionylcarnitine	0.1	0.374	0.894
LysoPC(14:0/0:0)	0.14	0.38	0.894
Docosahexaenoic acid	-0.17	0.381	0.894

LysoPE(20:5(5Z,8Z,11Z,14Z,17Z)/0:0)	0.14	0.396	0.894
5'-Methylthioadenosine	0.08	0.398	0.894
8 9-DiHETrE (pg/ml)	-0.1	0.4	0.894
LysoPE(22:6(4Z,7Z,10Z,13Z,16Z,19Z)/0:0)	0.1	0.401	0.894
LysoPE(18:0/0:0)	0.11	0.412	0.894
Niacinamide	0.11	0.412	0.894
15-oxo-ETE (pg/ml)	-0.18	0.413	0.894
L-Palmitoylcarnitine	-0.15	0.413	0.894
LysoPE(16:0/0:0)	0.1	0.414	0.894
(5Z,8Z,11Z,14Z,17Z)-Icosa-5,8,11,14,17-pentaenoylcarnitine	-0.14	0.414	0.894
LysoPC(20:1(11Z)/0:0)	-0.14	0.416	0.894
Xanthine	0.16	0.417	0.894
12(S)-HEPE (pg/ml)	0.23	0.418	0.894
gamma-Glutamyltryptophan	0.06	0.421	0.894
3-Hydroxyoctanoic acid	0.12	0.421	0.894
L,L-Cyclo(leucylprolyl)	0.18	0.424	0.895
Docosapentaenoic acid (22n-3)	-0.22	0.431	0.904
2-Hydroxystearic acid	-0.21	0.439	0.904
LysoPC(0:0/16:1)	0.12	0.445	0.904
Hexadecanedioic acid	0.09	0.451	0.904
12-OxoETE (pg/ml)	-0.16	0.453	0.904
20-HETE (pg/ml)	-0.12	0.453	0.904
Prolylhydroxyproline / Pyroglutamylvaline	-0.11	0.455	0.904
Guanosine	0.12	0.455	0.904
Hexose	-0.08	0.46	0.904
Oxoglutaric acid	0.06	0.461	0.904
8(S)-HETE (pg/ml)	-0.15	0.464	0.904
Chenodeoxycholic acid	0.27	0.467	0.904
LysoPC(22:4(7Z,10Z,13Z,16Z)/0:0)	-0.08	0.47	0.904
LysoPC(0:0/14:0)	0.12	0.472	0.904
(+-)11-HETE (pg/ml)	0.17	0.473	0.904
3-Hydroxyisovalerylcarnitine	-0.09	0.475	0.904
N2,N2-Dimethylguanosine	-0.09	0.477	0.904
LysoPC(20:4(5Z,8Z,11Z,14Z)/0:0)	-0.06	0.479	0.904
D-Leucic acid	-0.06	0.488	0.909
Citraconic acid	-0.14	0.488	0.909
Pimelic acid / 3-Methyladipic acid	-0.04	0.495	0.909
4-Hydroxyhippuric acid	0.12	0.496	0.909
LysoPC(18:1(9Z)/0:0)	-0.04	0.505	0.909
LysoPC(0:0/20:1)	-0.08	0.506	0.909
LysoPC(0:0/20:2)	-0.07	0.506	0.909
LysoPE(0:0/16:0)	0.07	0.509	0.909
LysoPE(0:0/18:0)	0.08	0.509	0.909
12(13)-EpOME (pg/ml)	0.19	0.514	0.909
3-Carboxy-4-methyl-5-propyl-2-furanpropionic acid	0.14	0.514	0.909
Quinic acid	0.14	0.515	0.909

PGD2 (pg/ml)	0.25	0.519	0.909
Isobutyryl-L-carnitine	0.11	0.521	0.909
LysoPC(20:5(5Z,8Z,11Z,14Z,17Z)/0:0)	0.1	0.523	0.91
LysoPC(16:1(9Z)/0:0)	0.09	0.529	0.915
Arachidonic acid	-0.12	0.542	0.915
Palmitic acid	-0.12	0.544	0.915
Uracil	-0.07	0.545	0.915
LysoPE(0:0/22:6(4Z,7Z,10Z,13Z,16Z,19Z))	0.07	0.547	0.915
4-Coumaryl alcohol	0.21	0.55	0.915
12-HETE (pg/ml)	-0.16	0.552	0.915
11(12)-EpETrE (pg/ml)	-0.12	0.552	0.915
LysoPC(0:0/20:4)	-0.07	0.553	0.915
MG(18:1(9Z)/0:0/0:0)	0.1	0.557	0.915
8(9)-EpETrE (pg/ml)	-0.13	0.56	0.915
LysoPC(15:0/0:0)	0.07	0.565	0.915
Azelaic acid	0.09	0.57	0.915
(S)-3-Hydroxyisobutyric acid	-0.08	0.573	0.915
3-Hydroxycapric acid	-0.08	0.575	0.915
Hypoxanthine	-0.09	0.576	0.915
4-Hydroxybenzoic acid	-0.14	0.576	0.915
3-Methylcrotonylglycine / Tiglylglycine	0.1	0.577	0.915
Stearoylcarnitine	-0.1	0.585	0.921
Kynurenic acid	0.08	0.587	0.921
N-Acetyl-L-alanine	0.03	0.59	0.921
D-Glucoheptose	-0.04	0.603	0.922
LysoPC(20:2(11Z,14Z)/0:0)	-0.09	0.605	0.922
LysoPE(O-16:0/0:0)	-0.09	0.607	0.922
Succinyladenosine	0.06	0.608	0.922
9(S)-HODE (pg/ml)	0.13	0.609	0.922
Hexanoylcarnitine	-0.11	0.611	0.922
LysoPC(0:0/20:5)	0.09	0.614	0.922
LysoPC(0:0/18:0(OH))	0.09	0.619	0.922
N-Formyl-L-methionine	0.03	0.62	0.922
LysoPC(P-18:0/0:0)	-0.09	0.624	0.922
12-Hydroxystearic acid	-0.07	0.628	0.922
2-Hydroxyadipic acid / 3-Hydroxyadipic acid / 3-Hydroxymethylglutaric acid	-0.03	0.631	0.922
LysoPC(18:0(OH)/0:0)	0.07	0.632	0.922
Citric acid	-0.01	0.632	0.922
(±)-2-Hydroxy-4-(methylthio)butanoic acid	0.05	0.635	0.922
LysoPE(22:4(7Z,10Z,13Z,16Z)/0:0)	-0.04	0.636	0.922
Cytosine	-0.06	0.645	0.929
2,3,4,5,6,7-Hexahydroxyheptanoic acid	0.02	0.646	0.929
Glutarylcarnitine	-0.06	0.664	0.945
(+-)9-HETE (pg/ml)	-0.13	0.664	0.945
Isocitric acid	-0.02	0.674	0.945
5-HETE (pg/ml)	-0.15	0.68	0.945
6-keto-PGF1a (pg/ml)	0.25	0.68	0.945

L-Kynurenine	0.04	0.683	0.945
Levonorgestrel	-0.08	0.685	0.945
Epinephrine	0.05	0.686	0.945
LysoPC(18:4(6Z,9Z,12Z,15Z)/0:0)	0.07	0.686	0.945
LysoPC(0:0/17:0)	-0.06	0.687	0.945
Taurochenodesoxycholic acid	-0.06	0.689	0.945
LysoPE(O-18:0/0:0)	-0.07	0.701	0.957
gamma-Aminobutyric acid	-0.03	0.713	0.957
LysoPC(14:1(9Z)/0:0)	0.06	0.717	0.957
Oleamide	-0.07	0.719	0.957
5-Hydroxyindoleacetic acid	0.04	0.719	0.957
LysoPC(22:6(4Z,7Z,10Z,13Z,16Z,19Z)/0:0)	-0.03	0.722	0.957
Isoleucyl-Leucine	0.05	0.723	0.957
13-HODE (pg/ml)	0.09	0.728	0.957
LysoPC(0:0/22:5)	-0.03	0.735	0.957
LysoPE(0:0/20:4(5Z,8Z,11Z,14Z))	0.03	0.741	0.957
LysoPC(0:0/20:3)	-0.04	0.746	0.957
Methylguanosine	-0.02	0.749	0.957
LysoPC(0:0/19:0)	-0.05	0.752	0.957
Trigonelline	0.07	0.756	0.957
Octadecanedioic acid	0.03	0.761	0.957
17-HDOHE (pg/ml)	-0.07	0.763	0.957
LysoPC(20:3(8Z,11Z,14Z)/0:0)	-0.03	0.765	0.957
LysoPC(18:2(9Z,12Z)/0:0)	-0.02	0.766	0.957
Glyceric acid	-0.03	0.767	0.957
2,4-Dihydroxybenzoic acid / 2-Pyrocatechuic acid	-0.06	0.775	0.957
LysoPE(0:0/18:3(6Z,9Z,12Z))	0.04	0.779	0.957
alpha-Ketoisovaleric acid	0.02	0.78	0.957
Indole-3-carboxaldehyde	-0.04	0.781	0.957
LysoPC(0:0/16:0)	-0.03	0.782	0.957
Malic acid	-0.02	0.791	0.957
LysoPC(0:0/18:2(9Z,12Z))	-0.03	0.793	0.957
LysoPE(20:4(5Z,8Z,11Z,14Z)/0:0)	0.02	0.794	0.957
3-Methoxytyrosine	-0.03	0.795	0.957
15-HETE (pg/ml)	-0.04	0.796	0.957
2',4'-Dihydroxyacetophenone (Resorcinol monoacetate)	-0.1	0.797	0.957
Itaconic acid	-0.03	0.799	0.957
Pyroglutamic acid	0.02	0.8	0.957
2-Hydroxydecanoate	0.03	0.804	0.957
2-Hydroxymyristoylcarnitine	-0.05	0.804	0.957
LysoPC(0:0/22:6)	-0.04	0.807	0.957
Serotonin	-0.26	0.809	0.957
Taurocholic acid	-0.05	0.809	0.957
LysoPE(18:3(9Z,12Z,15Z)/0:0)	0.04	0.81	0.957
Bis(2-ethylhexyl)phthalate	0.02	0.823	0.969
Phenylalanylisoleucine	0.02	0.828	0.971

9(10)-EpOME (pg/ml)	-0.06	0.835	0.973
5(s)6(R)-LXA4 (pg/ml)	-0.05	0.837	0.973
Isoleucyl-Isoleucine	0.03	0.838	0.973
Phenylalanylphenylalanine	-0.02	0.845	0.977
13-oxo-ODE (pg/ml)	0.04	0.86	0.984
(R)-3-Hydroxy-hexadecanoic acid	-0.03	0.862	0.984
7-Methylguanine	-0.01	0.865	0.984
Creatinine	0.01	0.865	0.984
LysoPE(0:0/18:1(9Z))	-0.02	0.873	0.984
LysoPC(12:0/0:0)	0.03	0.877	0.984
Pantothenic acid	0.02	0.879	0.984
LysoPC(20:0/0:0)	0.03	0.882	0.984
1-Methyladenosine	-0.01	0.883	0.984
Hydroxyoctanoic acid	-0.02	0.885	0.984
LysoPE(18:1(9Z)/0:0)	-0.02	0.888	0.984
Phenylalanyltryptophan / Tryptophyl-Phenylalanine	-0.03	0.889	0.984
LysoPE(P-16:0/0:0)	-0.02	0.89	0.984
Thyroxine	-0.01	0.896	0.988
LysoPE(P-18:0/0:0)	0.02	0.906	0.992
LysoPC(O-16:0/0:0)	-0.02	0.908	0.992
Bilirubin	0.02	0.909	0.992
5 6-DiHETrE (pg/ml)	0.02	0.918	0.994
LysoPC(19:0/0:0)	0.02	0.919	0.994
LysoPE(0:0/18:2(9Z,12Z))	0.02	0.922	0.994
4-Acetamidobutanoic acid	-0.01	0.925	0.994
3-(3-Hydroxyphenyl)-3-hydroxypropanoic acid / 4-Hydroxyphenyllactic acid	-0.01	0.927	0.994
5(6)-EpETrE (pg/ml)	-0.01	0.934	0.994
N-(3-acetamidopropyl)pyrrolidin-2-one	0.01	0.936	0.994
LysoPC(18:0/0:0)	0.01	0.943	0.994
2-Methoxybenzoic acid	-0.01	0.943	0.994
3-Methylglutarylcarnitine	0.01	0.945	0.994
Eicosapentaenoic acid	0.01	0.95	0.994
LysoPC(0:0/15:0)	-0.01	0.95	0.994
LysoPC(18:3(9Z,12Z,15Z)/0:0)	-0.01	0.958	0.994
Isovalerylcarnitine	-0.01	0.965	0.994
LysoPC(22:5(4Z,7Z,10Z,13Z,16Z)/0:0)	0.01	0.97	0.994
5-oxo-ETE (pg/ml)	-0.01	0.972	0.994
LysoPC(16:0/0:0)	0	0.975	0.994
2-Hydroxy-3-methylpentanoic acid	0	0.975	0.994
Sebacic acid	0	0.975	0.994
LysoPC(0:0/18:0)	0	0.977	0.994
Stearic acid	0.01	0.979	0.994
D-Phenyllactic acid	0	0.984	0.994
LysoPC(0:0/18:3)	0	0.986	0.994
LysoPE(18:2(9Z,12Z)/0:0)	0	0.987	0.994
Cholic acid	0	0.99	0.994

LysoPC(17:0/0:0)	0	0.991	0.994
L-Proline	0	0.998	0.998

Supplementary Table S9. The differences in relative metabolomic concentrations between smokers and non-smokers in collagenous colitis

variable	mean difference	p_value	adj_p_value
Serotonin	6.97	<0.001	<0.001
Cholic acid	-1.08	0.001	0.129
2,4-Dihydroxybenzoic acid / 2-Pyrocatechuic acid	0.71	0.001	0.152
L-Proline	0.2	0.011	0.539
gamma-Aminobutyric acid	-0.2	0.012	0.539
Succinyladenosine	0.27	0.013	0.539
7-Methylguanine	0.18	0.016	0.539
Malic acid	-0.18	0.016	0.539
6-trans-LTB4 (pg/ml)	0.96	0.016	0.539
Quinic acid	0.54	0.017	0.539
Docosahexaenoic acid	-0.48	0.019	0.539
14 15-DiHETrE (pg/ml)	0.24	0.021	0.539
4-Hydroxybenzoic acid	0.62	0.027	0.539
Glyceric acid	-0.25	0.028	0.539
gamma-Glutamylalanine	0.32	0.028	0.539
L-Acetylcarnitine	-0.3	0.029	0.539
N-Acetyl-L-methionine	0.2	0.031	0.539
LysoPC(0:0/22:5)	-0.2	0.033	0.539
4-Pyridoxic acid	-0.55	0.036	0.539
LysoPC(22:6(4Z,7Z,10Z,13Z,16Z,19Z)/0:0)	-0.2	0.037	0.539
Cinnamoylglycine	-0.65	0.038	0.539
Phenylalanylphenylalanine	0.3	0.042	0.539
3a,7b,12a-Trihydroxyoxocholanyl-Glycine	-0.4	0.042	0.539
AICAR	-0.23	0.043	0.539
Uric acid	-0.15	0.046	0.539
LysoPC(0:0/22:6)	-0.31	0.046	0.539
S-Adenosylhomocysteine	-0.36	0.048	0.539
Hexose	0.21	0.05	0.539
LysoPE(P-16:0/0:0)	-0.34	0.051	0.539
LysoPC(0:0/20:2)	-0.18	0.053	0.539
alpha-Linolenic acid	-0.55	0.053	0.539
gamma-Glutamylmethionine	0.27	0.054	0.539
Indoleacetic acid	-0.29	0.055	0.539
Trigonelline	0.43	0.057	0.549
Isoleucyl-Isoleucine	0.25	0.061	0.551
Citraconic acid	0.39	0.062	0.551
Threonic acid	-0.38	0.065	0.551
PGD2 (pg/ml)	0.67	0.067	0.551
(S)-3-Hydroxyisobutyric acid	-0.26	0.069	0.551
Phenylalanylglutamic acid / gamma-Glutamylphenylalanine	0.17	0.072	0.551
3-Methylcrotonylglycine / Tiglylglycine	-0.33	0.072	0.551
LysoPC(17:0/0:0)	-0.31	0.072	0.551

Tetradecanedioic acid	-0.36	0.073	0.551
L,L-Cyclo(leucylprolyl)	0.43	0.079	0.577
Chenodeoxycholic acid	-0.61	0.08	0.577
Sebacic acid	-0.17	0.082	0.577
LysoPC(20:1(11Z)/0:0)	-0.29	0.085	0.583
Paraxanthine	0.36	0.096	0.583
LysoPC(0:0/20:3)	-0.22	0.099	0.583
N-gamma-Glutamylglutamine	0.17	0.102	0.583
LysoPE(0:0/22:6(4Z,7Z,10Z,13Z,16Z,19Z))	-0.17	0.102	0.583
LysoPC(0:0/20:1)	-0.18	0.105	0.583
LysoPC(22:4(7Z,10Z,13Z,16Z)/0:0)	-0.17	0.108	0.583
LysoPC(0:0/20:4)	-0.17	0.108	0.583
LysoPC(15:0/0:0)	-0.21	0.11	0.583
LysoPC(O-16:0/0:0)	-0.26	0.11	0.583
9(10)-EpOME (pg/ml)	-0.39	0.11	0.583
15-HETE (pg/ml)	0.34	0.113	0.583
LysoPC(0:0/17:0)	-0.23	0.113	0.583
3-Hydroxytetradecanoic acid	-0.15	0.113	0.583
Cervonylcarnitine	-0.26	0.116	0.583
Salicylic acid	0.87	0.118	0.583
Epinephrine	0.17	0.119	0.583
gamma-Glutamylisoleucine	0.3	0.119	0.583
LysoPC(20:3(8Z,11Z,14Z)/0:0)	-0.16	0.12	0.583
N-Formyl-L-methionine	0.09	0.121	0.583
LysoPC(20:2(11Z,14Z)/0:0)	-0.25	0.123	0.583
Hypoxanthine	-0.24	0.127	0.583
alpha-Dimorphecolic acid	-0.25	0.13	0.583
LysoPE(P-18:0/0:0)	-0.3	0.131	0.583
Leucyl-Aspartate / gamma-Glutamylvaline	0.33	0.132	0.583
Creatinine	0.11	0.134	0.583
Pimelic acid / 3-Methyladipic acid	-0.08	0.134	0.583
Citrulline	0.14	0.134	0.583
Linoleic acid	-0.31	0.135	0.583
LysoPE(O-16:0/0:0)	-0.25	0.138	0.591
Creatine	-0.26	0.149	0.609
Thyroxine	-0.18	0.153	0.609
Azelaic acid	-0.23	0.157	0.609
Phenylalanylisoleucine	0.15	0.158	0.609
D-Leucic acid	0.13	0.159	0.609
9 10-DiHOME (pg/ml)	-0.39	0.16	0.609
Oleic acid / Elaidic acid	-0.35	0.161	0.609
LysoPC(19:0/0:0)	-0.29	0.161	0.609
4-Hydroxyproline	0.11	0.162	0.609
Tiglylcarnitine	-0.2	0.162	0.609
Acetoacetic acid	-0.36	0.163	0.609
Docosapentaenoic acid (22n-3)	-0.39	0.169	0.621
Pantothenic acid	-0.19	0.171	0.621
Eicosapentaenoic acid	-0.3	0.172	0.621

3-Hydroxycapric acid	-0.22	0.174	0.621
Hydroxyoctanoic acid	-0.21	0.176	0.622
Guanine	-0.14	0.18	0.622
LysoPC(0:0/22:4)	-0.15	0.18	0.622
Isoleucyl-Leucine	0.17	0.186	0.632
LysoPC(16:0(OH)/0:0)	-0.22	0.189	0.632
Tetradecanoylcarnitine	-0.26	0.19	0.632
Cytosine	-0.19	0.191	0.632
2-Hydroxymyristic acid	-0.21	0.196	0.643
L-Methionine	-0.14	0.2	0.643
15(S)-HETrE (pg/ml)	0.36	0.202	0.643
11 12-DiHETrE (pg/ml)	0.16	0.203	0.643
LysoPE(16:0/0:0)	-0.15	0.204	0.643
Cortisol / hydrocortisone	-0.28	0.209	0.654
5-HETE (pg/ml)	0.57	0.219	0.656
LysoPC(0:0/19:0)	-0.17	0.219	0.656
L-Tyrosine	-0.11	0.22	0.656
5(s)6(R)-LXA4 (pg/ml)	0.33	0.221	0.656
(+-)11-HETE (pg/ml)	0.35	0.223	0.656
Asymmetric dimethylarginine	0.14	0.228	0.656
3, 5-Tetradecadiencarnitine	-0.27	0.233	0.656
Betaine	-0.06	0.234	0.656
LysoPC(16:0/0:0)	-0.04	0.234	0.656
Bilirubin	-0.22	0.234	0.656
Phenylalanyltryptophan / Tryptophyl-Phenylalanine	0.19	0.236	0.656
2-Hydroxyhexadecanoic acid	-0.19	0.237	0.656
8(S)-HETE (pg/ml)	0.32	0.24	0.656
Dodecanoylcarnitine	-0.26	0.24	0.656
Taurochenodesoxycholic acid	0.18	0.24	0.656
Niacinamide	-0.14	0.244	0.658
gamma-Glutamylleucine	0.2	0.25	0.658
Ketoleucine	-0.14	0.252	0.658
Caffeine	0.24	0.252	0.658
1-Methyladenosine	0.1	0.254	0.658
LysoPC(0:0/15:0)	-0.18	0.255	0.658
Inosine	-0.16	0.255	0.658
trans-2-Dodecenoylcarnitine	-0.28	0.261	0.669
LysoPC(20:4(5Z,8Z,11Z,14Z)/0:0)	-0.09	0.268	0.68
12 13-DiHOME (pg/ml)	-0.27	0.27	0.681
LysoPC(P-18:0/0:0)	-0.2	0.274	0.685
Icosa-8,11,14-trienoylcarnitine	-0.17	0.28	0.694
D-Phenyllactic acid	0.18	0.288	0.699
Octadecanedioic acid	-0.1	0.288	0.699
L-Palmitoylcarnitine	-0.19	0.292	0.699
4-Coumaryl alcohol	-0.35	0.292	0.699
LysoPE(22:6(4Z,7Z,10Z,13Z,16Z,19Z)/0:0)	-0.12	0.293	0.699
5(6)-EpETrE (pg/ml)	-0.19	0.295	0.701

N2,N2-Dimethylguanosine	0.12	0.302	0.707
Oxoglutaric acid	-0.08	0.303	0.707
LysoPC(14:0/0:0)	-0.17	0.306	0.707
Linoleylcarnitine	-0.17	0.307	0.707
13-HODE (pg/ml)	-0.25	0.311	0.712
9,12-Hexadecadienoylcarnitine	-0.21	0.316	0.718
Arachidonic acid	-0.18	0.335	0.75
Glycocholic acid	-0.28	0.335	0.75
Pyroglutamic acid	-0.07	0.345	0.769
Isocitric acid	-0.04	0.349	0.773
cis-4-Decenoylcarnitine	-0.17	0.354	0.777
3-Methoxytyrosine	-0.09	0.359	0.784
Capryloylglycine	-0.16	0.372	0.795
Hexadecanedioic acid	-0.11	0.373	0.795
Itaconic acid	0.12	0.378	0.795
2-Hydroxydecanoate	-0.12	0.381	0.795
LysoPE(22:5(4Z,7Z,10Z,13Z,16Z)/0:0)	0.09	0.384	0.795
L-Leucine	-0.06	0.389	0.795
LysoPC(0:0/18:1(9Z))	0.07	0.389	0.795
1-Aminocyclopropanecarboxylic acid	-0.06	0.39	0.795
Xanthine	-0.13	0.396	0.795
17-HDOHE (pg/ml)	0.24	0.402	0.795
LysoPE(O-18:0/0:0)	-0.14	0.406	0.795
alpha-Ketoisovaleric acid	-0.07	0.406	0.795
Indolelactic acid	0.08	0.406	0.795
20-HETE (pg/ml)	-0.14	0.409	0.795
Valerylcarnitine	-0.13	0.411	0.795
LysoPC(20:5(5Z,8Z,11Z,14Z,17Z)/0:0)	-0.13	0.413	0.795
PGE2 (pg/ml)	0.47	0.415	0.795
LysoPC(0:0/20:5)	-0.14	0.416	0.795
2,3,4,5,6,7-Hexahydroxyheptanoic acid	0.03	0.417	0.795
Myristoleoylcarnitine	-0.2	0.421	0.795
3-Hydroxydodecanoic acid	-0.14	0.425	0.795
MG(18:1(9Z)/0:0/0:0)	0.13	0.429	0.795
3beta,7alpha-Dihydroxy-5-cholestenoate	0.11	0.431	0.795
Decanoylcarnitine	-0.17	0.434	0.795
Gamma-linolenyl arnitine	-0.13	0.434	0.795
Dodecanedioic acid	-0.09	0.437	0.795
3-Hydroxyoctanoic acid	-0.11	0.438	0.795
Methylguanosine	0.05	0.438	0.795
Homo-L-arginine	0.11	0.44	0.795
3-Carboxy-4-methyl-5-propyl-2-furanpropionic acid	-0.19	0.444	0.795
Suberic acid	0.05	0.445	0.795
LysoPC(18:2(9Z,12Z)/0:0)	-0.05	0.446	0.795
LysoPC(0:0/14:0)	-0.13	0.447	0.795
LysoPE(18:0/0:0)	-0.09	0.447	0.795
2-Hydroxycaproic acid	-0.08	0.453	0.795

9-Hexadecenoylcarnitine	-0.15	0.454	0.795
Palmitic acid	-0.15	0.455	0.795
Glutarylcarnitine	-0.09	0.469	0.815
Phenylacetylglutamine	-0.19	0.471	0.815
(±)-2-Hydroxy-4-(methylthio)butanoic acid	0.08	0.474	0.815
LysoPE(0:0/16:0)	-0.07	0.481	0.819
Oleamide	-0.12	0.481	0.819
LysoPC(12:0/0:0)	0.16	0.485	0.819
Isobutyryl-L-carnitine	0.14	0.486	0.819
Lithocholic acid glycine conjugate	0.11	0.493	0.826
L-Kynurenine	-0.07	0.497	0.828
3-Hydroxyisovalerylcarnitine	-0.09	0.504	0.828
LysoPC(18:1(9Z)/0:0)	-0.04	0.506	0.828
5'-Methylthioadenosine	-0.06	0.507	0.828
6-keto-PGF1a (pg/ml)	0.4	0.507	0.828
gamma-Glutamyltryptophan	0.05	0.511	0.83
2-Hydroxyadipic acid / 3-Hydroxyadipic acid / 3-Hydroxymethylglutaric acid	-0.03	0.518	0.831
LysoPC(0:0/16:0)	-0.06	0.518	0.831
(5Z,8Z,11Z,14Z,17Z)-Icosa-5,8,11,14,17-pentaenoylcarnitine	-0.11	0.519	0.831
2-Hydroxymyristoylcarnitine	-0.13	0.526	0.838
Ursodeoxycholic acid	0.16	0.535	0.839
(R)-3-Hydroxy-hexadecanoic acid	-0.09	0.541	0.839
LysoPE(0:0/18:3(6Z,9Z,12Z))	0.1	0.543	0.839
13-oxo-ODE (pg/ml)	-0.17	0.544	0.839
Arachidonoylcarnitine	-0.1	0.545	0.839
Bis(2-ethylhexyl)phthalate	-0.05	0.556	0.839
L-Lysine	-0.04	0.558	0.839
LysoPC(22:5(4Z,7Z,10Z,13Z,16Z)/0:0)	-0.08	0.558	0.839
4-Acetamidobutanoic acid	-0.03	0.561	0.839
Oleoylcarnitine	-0.1	0.562	0.839
LysoPC(0:0/18:0(OH))	-0.13	0.564	0.839
Guanosine	-0.09	0.566	0.839
LysoPC(18:0/0:0)	-0.04	0.568	0.839
2-Octenoylcarnitine	0.12	0.572	0.839
LysoPE(18:3(9Z,12Z,15Z)/0:0)	0.1	0.572	0.839
Indole-3-propionic acid	-0.26	0.572	0.839
Cortisone	-0.1	0.573	0.839
MG(18:2(9Z,12Z)/0:0/0:0)	-0.07	0.574	0.839
(7Z,10Z,13Z,16Z)-Docosatetraenoylcarnitine	-0.09	0.575	0.839
L-Octanoylcarnitine	-0.11	0.587	0.852
LysoPE(0:0/18:2(9Z,12Z))	0.08	0.598	0.86
Trimethylamine N-oxide	0.12	0.6	0.86
LysoPC(0:0/18:2(9Z,12Z))	-0.07	0.606	0.86
12(S)-HEPE (pg/ml)	0.16	0.608	0.86
(2E)-3-(4-hydroxy-3-methoxyphenyl)prop-2-enal	-0.13	0.609	0.86

2-Hydroxy-3-methylpentanoic acid	0.08	0.61	0.86
Succinic acid	-0.03	0.613	0.86
Theobromine	0.13	0.614	0.86
2-Hydroxystearic acid	-0.13	0.621	0.86
Prolylhydroxyproline / Pyroglutamylvaline	0.07	0.622	0.86
8(9)-EpETrE (pg/ml)	-0.12	0.623	0.86
L-Isoleucine	-0.04	0.629	0.86
11(12)-EpETrE (pg/ml)	-0.1	0.63	0.86
Acetaminophen	0.46	0.631	0.86
Undecanedioic acid	0.04	0.633	0.86
Indole-3-carboxaldehyde	0.07	0.644	0.871
Adipic acid	0.03	0.65	0.874
Theophylline	0.09	0.651	0.874
12(13)-EpOME (pg/ml)	-0.14	0.653	0.874
LysoPC(0:0/18:0)	-0.07	0.656	0.874
3-Hydroxybutyrylcarnitine	0.2	0.659	0.874
LysoPC(20:0/0:0)	-0.08	0.671	0.886
1,11-Undecanedicarboxylic acid	0.04	0.676	0.887
8-iso-PGE2 (pg/ml)	0.21	0.679	0.887
3-Hydroxyoctadecenoylcarnitine	-0.1	0.68	0.887
2-Methoxybenzoic acid	0.04	0.69	0.894
12-HETE (pg/ml)	0.1	0.692	0.894
D-Glucoheptose	0.03	0.693	0.894
Chenodeoxycholic acid glycine conjugate	-0.08	0.702	0.898
3-(3-Hydroxyphenyl)-3-hydroxypropanoic acid / 4-Hydroxyphenyllactic acid	-0.03	0.702	0.898
LysoPE(0:0/18:0)	-0.04	0.714	0.909
3-Hydroxybutyric acid	-0.1	0.723	0.916
5-Hydroxyindoleacetic acid	-0.04	0.724	0.916
2-Hydroxy-3-methylbutyric acid	0.07	0.732	0.922
Hippuric acid	0.08	0.746	0.936
2',4'-Dihydroxyacetophenone (Resorcinol monoacetate)	-0.13	0.75	0.937
LysoPE(20:4(5Z,8Z,11Z,14Z)/0:0)	-0.03	0.759	0.943
12-Hydroxystearic acid	0.04	0.76	0.943
Pipecolic acid	-0.03	0.782	0.964
Stearoylcarnitine	0.05	0.785	0.964
Lenticin	0.14	0.786	0.964
L-Sorbose	0.06	0.794	0.964
LysoPE(18:2(9Z,12Z)/0:0)	0.04	0.798	0.964
LTB4 (pg/ml)	0.14	0.799	0.964
Sphingosine 1-phosphate	-0.02	0.8	0.964
alpha-Chaconine	-0.09	0.803	0.964
(+-)9-HETE (pg/ml)	0.09	0.805	0.964
Propionylcarnitine	-0.03	0.807	0.964
TxB2 (pg/ml)	-0.17	0.812	0.967
Deoxycholic acid	-0.08	0.818	0.969
4-Hydroxyhippuric acid	-0.04	0.824	0.969

Indoleacetaldehyde	0.02	0.828	0.969
Glycoursodeoxycholic acid	-0.06	0.829	0.969
LysoPC(14:1(9Z)/0:0)	0.04	0.831	0.969
5-oxo-ETE (pg/ml)	0.07	0.838	0.969
LysoPE(0:0/20:4(5Z,8Z,11Z,14Z))	-0.02	0.84	0.969
LysoPC(18:0(OH)/0:0)	0.03	0.843	0.969
LysoPC(18:3(9Z,12Z,15Z)/0:0)	-0.03	0.844	0.969
3-Hydroxyisovaleric acid	-0.02	0.858	0.969
Docosapentaenoylcarnitine	-0.03	0.861	0.969
Deoxycholic acid glycine conjugate	-0.06	0.865	0.969
2-Hydroxylauroylcarnitine	0.03	0.868	0.969
Citric acid	0	0.871	0.969
9 10 13-TriHOME (pg/ml)	-0.03	0.874	0.969
LysoPE(20:5(5Z,8Z,11Z,14Z,17Z)/0:0)	0.03	0.876	0.969
(11Z)-Eicoseneoylcarnitine	-0.03	0.878	0.969
3-Methylglutarylcarnitine	0.03	0.881	0.969
N-Acetyl-L-alanine	0.01	0.883	0.969
N-Acetylserine	0.02	0.883	0.969
Kynurenic acid	-0.02	0.884	0.969
LysoPE(18:1(9Z)/0:0)	0.02	0.886	0.969
Stearic acid	0.03	0.888	0.969
9(S)-HODE (pg/ml)	-0.03	0.889	0.969
gamma-Glutamyltyrosine	0.02	0.891	0.969
Isovalerylcarnitine	-0.02	0.893	0.969
LysoPE(0:0/20:5(5Z,8Z,11Z,14Z,17Z))	0.02	0.896	0.969
Uridine/Pseudouridine	-0.01	0.897	0.969
5 6-DiHETrE (pg/ml)	0.02	0.905	0.974
Uracil	-0.01	0.91	0.976
LysoPC(18:4(6Z,9Z,12Z,15Z)/0:0)	-0.02	0.913	0.976
Butyrylcarnitine	0.02	0.918	0.978
8 9-DiHETrE (pg/ml)	0.01	0.928	0.979
Hexanoylcarnitine	-0.02	0.93	0.979
LysoPE(22:4(7Z,10Z,13Z,16Z)/0:0)	-0.01	0.936	0.979
L-Tryptophan	-0.01	0.936	0.979
Methylmalonylcarnitine	-0.01	0.937	0.979
Indoxyl sulfate	-0.01	0.938	0.979
LysoPE(0:0/18:1(9Z))	-0.01	0.94	0.979
12-OxoETE (pg/ml)	0.02	0.95	0.981
LysoPC(0:0/16:1)	0.01	0.95	0.981
N-(3-acetamidopropyl)pyrrolidin-2-one	-0.01	0.951	0.981
LysoPC(0:0/18:3)	-0.01	0.956	0.982
9 12 13-TriHOME (pg/ml)	-0.02	0.958	0.982
Taurocholic acid	0.01	0.966	0.987
15-oxo-ETE (pg/ml)	0.01	0.973	0.988
Phenylalanylproline	0	0.975	0.988
Glutaric acid	0	0.975	0.988
L-Phenylalanine	0	0.98	0.989
LysoPC(16:1(9Z)/0:0)	0	0.989	0.995

Levonorgestrel	0	0.994	0.995
Adenosine	0	0.995	0.995

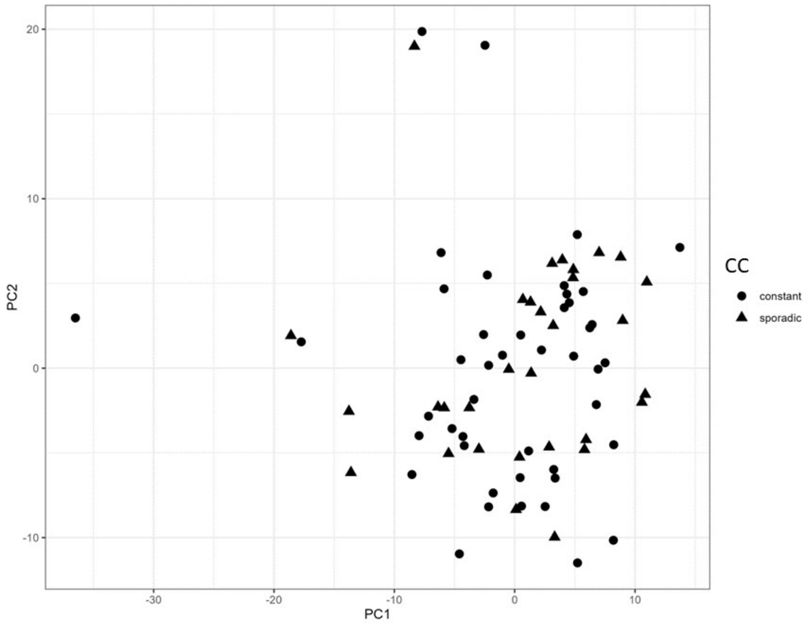

Supplementary Figure S1: Principal Component Analysis divided by one episode of or refractory collagenous colitis (CC)

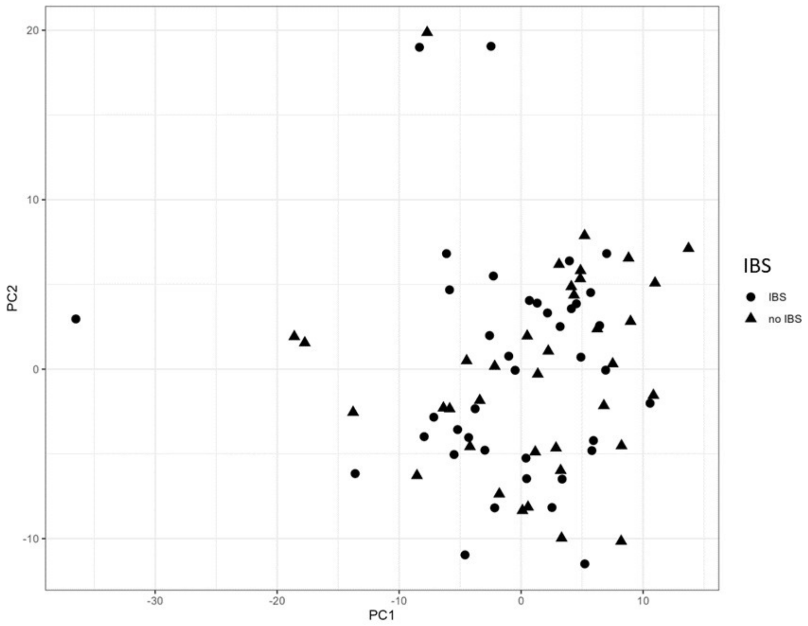

Supplementary Figure S2: Principal Component Analysis divided by presence of irritable bowel syndrome (IBS)-like symptoms or not

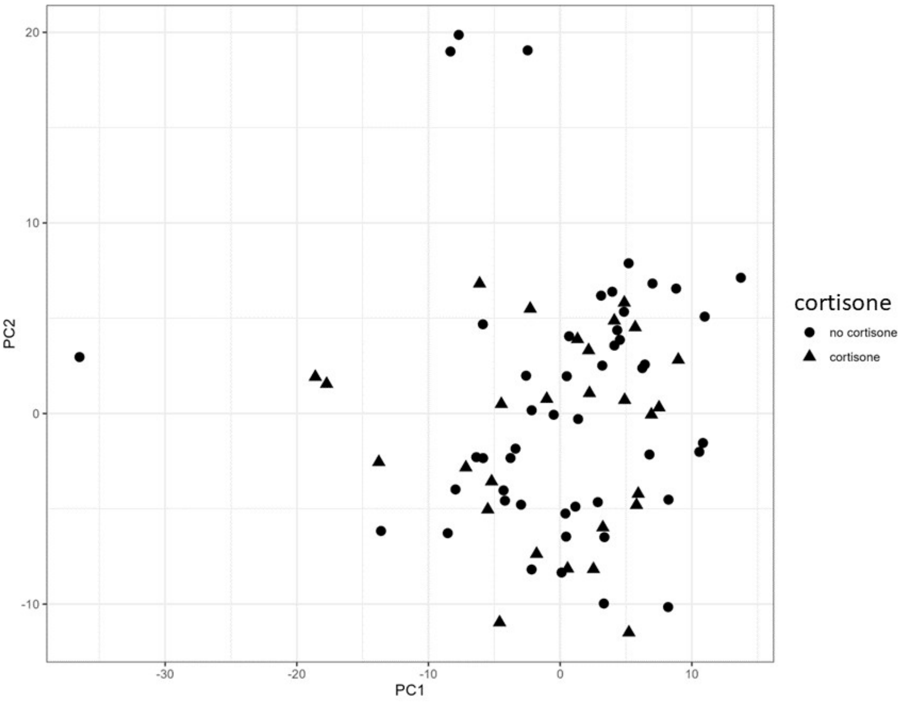

Supplementary Figure S3: Principal Component Analysis divided by use of corticosteroids or not

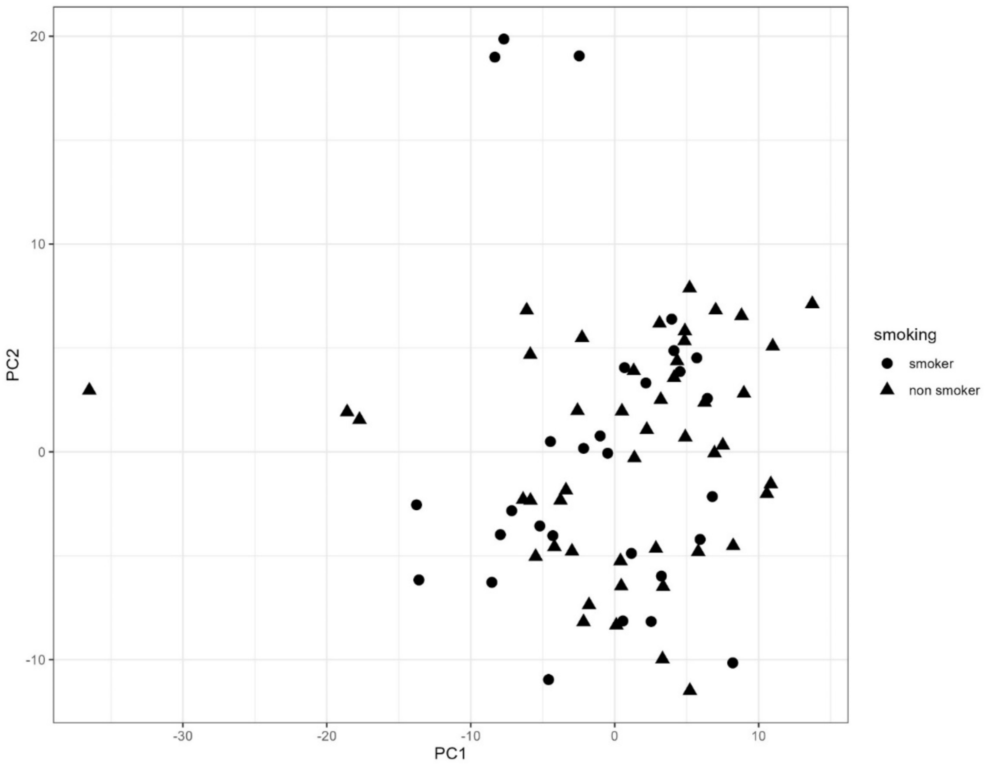

Supplementary Figure S4: Principal Component Analysis divided by smoking or non-smoking
